# The Synthesis and Evaluation of Amidoximes as Cytotoxic Agents on Model Bacterial *E. coli* Strains

**DOI:** 10.3390/ma14247577

**Published:** 2021-12-09

**Authors:** Jan Samsonowicz-Górski, Paweł Kowalczyk, Dominik Koszelewski, Anna Brodzka, Mateusz Szymczak, Karol Kramkowski, Ryszard Ostaszewski

**Affiliations:** 1 Institute of Organic Chemistry PAS, Kasprzaka 44/52, 01-224 Warsaw, Poland; jan.samsonowicz-gorski.stud@pw.edu.pl (J.S.-G.); anna.brodzka@icho.edu.pl (A.B.); ryszard.ostaszewski@icho.edu.pl (R.O.); 2 Department of Animal Nutrition, The Kielanowski Institute of Animal Physiology and Nutrition, Polish Academy of Sciences, Instytucka 3, 05-110 Jabłonna, Poland; 3 Department of Molecular Virology, Institute of Microbiology, Faculty of Biology, University of Warsaw, Miecznikowa 1, 02-096 Warsaw, Poland; mszymczak@biol.uw.edu.pl; 4 Department of Physical Chemistry, Medical University of Bialystok, Kilińskiego 1 Str., 15-089 Białystok, Poland; kkramk@wp.pl

**Keywords:** amidoximes, Boc-protected amidoximes, Boc-derived amidines, DNA-N-glycosylase, Fpg protein-formamidopyrimidine, lipopolysaccharide (LPS)

## Abstract

The biological research on newly synthesized amidoximes, Boc-protected amidoximes and Boc-derived amidines, obtained by a reduction of the parent amidoximes is reported, herein. Due to the presence of a free amino group in both amidines and amidoximes, these compounds can undergo various chemical reactions such as *N*-alkylation and *N*-acylation. One such reaction is Boc-protection, often used in organic synthesis to protect the amino and imino groups. Until now, Boc-protected amidoximes have not been tested for biological activity. Amidoxime derivatives were tested on bacterial *E. coli* strains. Initial cellular studies tests and digestion with Fpg after the modification of bacterial DNA, suggest that these compounds may have greater potential as antibacterial agents compared to antibiotics such as ciprofloxacin (ci), bleomycin (b) and cloxacillin (cl). The described compounds are highly specific for pathogenic *E. coli* strains on the basis of the model strains used and may be used in the future as new substitutes for commonly used antibiotics in clinical and hospital infections in the pandemic era.

## 1. Introduction

**Figure 1:**
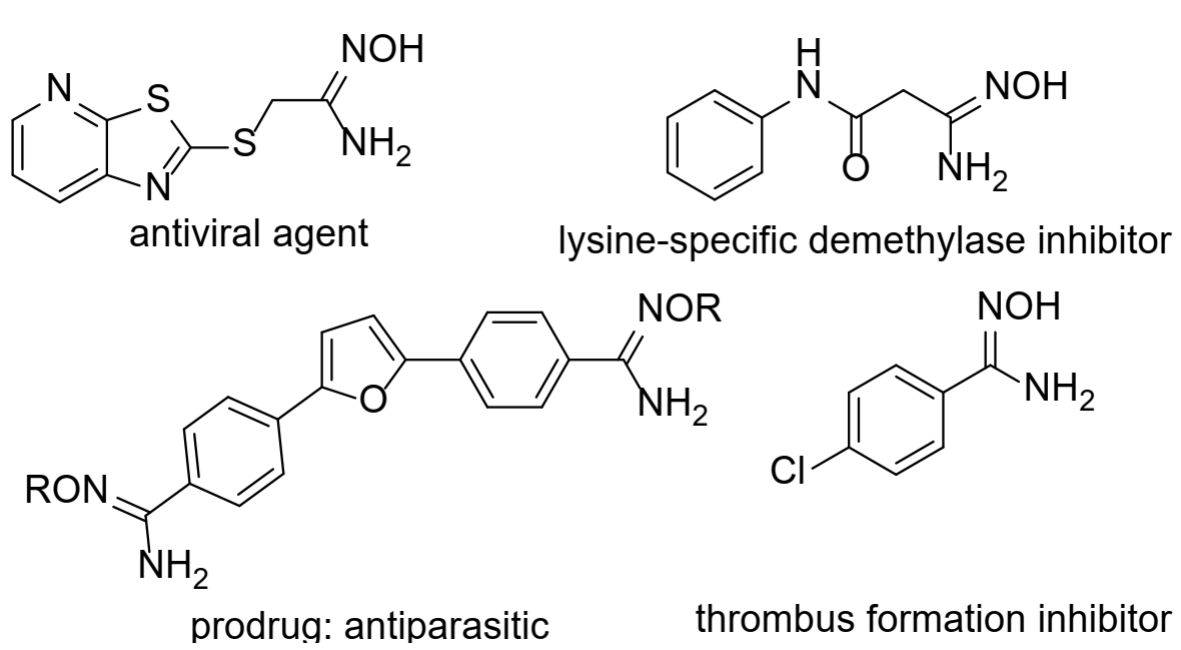
Pharmaceutically relevant amidoximes.

Amidoximes perform many activities on various microorganisms and complex eukaryotic organisms. Thus, they are intensively studied as potential drugs, prodrugs or fungicidal or bactericidal substances (Figure 1).

In natural metabolism oximes (derived from arginine) are donors of nitric oxide (NO) [1], which is known to be an important neurotransmitter and neuromodulator and agent targeted in medical treatment [2]. Thus, synthetic amidoximes have impact on natural metabolic pathways on mammalian organisms. Additionally, the cardiotonic activities of amidoximes have been reported [3]. Similar to keto- and aldoximes, amidoximes possess an ability to reactivate aceltylcholinesterase after poisoning with organophosphorus compounds [4]. Furthermore, inhibitory effect of amidoximes on lysine-specific demethylases, important in processes of gene expression, was investigated [5]. Additionally, amidoximes possess documented anti-microbial activity as inhibitors of SHC (squalene-hopene cyclase), which plays a crucial role in the process of stabilization of cell membrane (for instance in Alicyclobacillus acidocaldarius cells) [6]. Thus, amidoximes were examined for use in surface functionalization of nanofibers [7] or material utilized for adsorption of uranium from seawater [8]. Following anti-microorganism activity, anti-virial activity on influenza B-Mass virus [9] of pyridine amidoxime derivative was also proven. Amidoximes are reduced in vivo to corresponding amidines by organisms possessing active ARC (amidoxime reducing component). Such process may result in release of substances exhibiting anti-microbial activity. Drugs against pneumocystis, protozoan [1] or trypanosoma may be introduced this way [10]. Furthermore, reports have been made on amidine-containing inhibitors of serine proteases, which also enlarge their utility in medicine [11].

Analyzed K12 and R2-R4 strains of *Escherichia coli* (*E. coli*) are not only the dominant species of the human aerobic bacterial flora and are flora in various habitats in which people live, e.g., bathrooms, clinics and hospitals, but they can temporarily colonize the oropharynx and skin in healthy people. However, apart from saprophytic strains, harmless to humans, there are also strains of *Escherichia coli* that are pathogenic for humans and cause various forms of acute diarrhea. Infection usually occurs through contaminated food and water, as is the case with other bacterial diarrhea, and less frequently through indirect contact. In industrialized countries, pathogenic enteric strains of *Escherichia coli* are rarely a component of the intestinal flora of healthy people, therefore they are considered strictly pathogenic bacteria. When an appropriate volume of bacteria is ingested by a person susceptible to pathogenic infection, strains of *Escherichia coli* have the ability to cause inflammation of the small intestine and/or large intestine. Adequate gastric acidity has a disinfecting effect and protects to a certain extent against infection, therefore people with low gastric acidity are particularly susceptible to infections with pathogenic *Escherichia coli* strains. The source of the infection is a sick person or a vector (except for STEC/EHEC strains—the source is cattle). Intestinal diseases caused by pathogenic strains of *Escherichia coli* occur in the form of epidemics or as sporadic cases, with an increase in the incidence in the summer months, which is the rule for bacterial diarrhea. Therefore, we study these strains with the use of the analyzed amidoximes to investigate their etiology and the mechanism causing their resistance to many known and commonly used antibiotics [11,12,13,14,15,16,17,18,19,20,21,22,23,24,25,26,27,28,29,30,31,32,33,34,35,36,37,38,39,40,41,42,43,44,45,46,47,48].

Despite numbers of reports in the literature on amidoxime’s pharmacological and biological properties, their anti-bacterial activities are still being rediscovered, therefore there is still a need for additional research on their cytotoxic effect on selected hospital bacterial strains causing diseases associated with blood infections such as sepsis. Therefore, this article is an attempt to address this question. Until now, such compounds have not been tested for biological activity against pathogenic *E. coli* strains, so there is a need to clarify their role.

## 2. Materials and Methods

All compounds for the research were obtained from Sigma-Aldrich (Merck Life Science LTD division in Poznań, Poland)

## 3. Experimental Section

### 3.1. 2-(4-Methoxybenzylamino)-2-oxo-1-(4-cyanophenyl)ethyl phenylacetate

*p*-Methoxybenzylisocyanide (147.2 mg, 1 mmol) was added to a suspension of 4-cyanobenzaldehyde (131.1, 1 mmol) and phenylacetic acid (136.2 mg, 1 mmol) at room temperature. The reaction mixture was stirred at ambient temperature for 18 h. The crude product was purified by column chromatography with silica gel (hexanes/EtOAc) to afford the corresponding product as a colorless semi-solid in 72% yield (298 mg, 0.72 mmol). R_f_ 0.28 (hexanes/EtOAc, 7:3). ^1^H NMR (400 MHz, CDCl_3_) δ 7.63–7.53 (m, 2H), 7.44 (d, *J* = 8.1 Hz, 2H), 7.31–7.17 (m, 5H), 7.10–6.97 (m, 2H), 6.89–6.77 (m, 2H), 6.32 (s, 1H), 6.10 (s, 1H), 4.22 (d, *J* = 5.7 Hz, 2H), 3.79 (s, 3H), 3.72 (s, 2H); ^13^C NMR (100 MHz, CDCl_3_) δ 169.3, 167.1, 159.1, 140.5, 133.0, 132.3, 129.4, 129.1, 129.1, 128.9, 128.95 128.8, 128.8, 127.7, 127.5, 127.5, 118.3, 114.1, 112.5, 60.3, 42.8, 41.1, 20.9. Elemental analysis calculated for C_25_H_22_N_2_O_4_: C, 72.45%; H, 5.35%; N, 6.76%; found: C, 72.29%; H, 5.31%; N, 6.68%

### 3.2. 4-Cyanostilbene

The PdCl_2_ catalyst (0.02 g, 1 mol%) was added to a mixture of 4-bromobenzenecarbonitrile (182 mg, 1 mmol), *trans*-2-phenylvinylboronic acid (177 mg, 1.2 mmol), and K_2_CO_3_ (2 mmol) in 10 mL of EtOH with 1 mL of H_2_O, and stirred at room temperature. The reaction was followed by TLC. After the completion of the reaction, the mixture was washed with diethyl ether (3 × 10 mL) followed by deionized water (3 × 10 mL). The organic phase after separation of the catalyst was evaporated to obtain the crude product. The product was purified by column chromatography with silica gel (hexanes/EtOAc) to afford the corresponding product as a colorless solid in 82% yield (168 mg, 0.82 mmol); m.p. 117 °C, (Lit. 116–118 °C) [12]; ^1^H NMR (400 MHz, CDCl_3_) δ 7.66–7.59 (m, 2H), 7.59–7.50 (m, 4H), 7.43–7.36 (m, 2H), 7.36–7.28 (m, 1H), 7.21 (d, *J* = 16.3 Hz, 1H), 7.08 (d, *J* = 16.4 Hz, 1H); ^13^C NMR (100 MHz, CDCl_3_) δ 141.8, 136.3, 132.4, 128.8, 128.6, 126.9, 119.0, 110.6. ^1^H- and ^13^CNMR data were in accordance with those reported in the literature [13].

### 3.3. 4-Methoxybiphenyl-4-carbonitrile

A mixture of 4-bromobenzenecarbonitrile (182 mg, 1 mmol), 4-methoxyphenylboronic acid (152 mg, 1 mmol), PdCl_2_ (0.0025 mmol, 0.44 mg), K_2_CO_3_ (1 mmol) was stirred in distilled water (3 mL) and ethanol (3 mL) at room temperature. The reaction was followed by TLC. After the completion of the reaction, the mixture was quenched by brine (15 mL), extracted with diethyl ether (4 × 10 mL), dried by anhydrous MgSO_4_, concentrated under vacuum. The crude product was purified by column chromatography with silica gel (hexanes/EtOAc) to afford the corresponding product as a colorless solid in 95% yield (198 mg, 0.95 mmol); m.p. 102 °C (Lit. m.p. 101–103 °C) [14]; ^1^H NMR (400 MHz, CDCl_3_) δ 7.61 (q, *J* = 8.7 Hz, 4H), 7.51 (d, *J* = 8.8 Hz, 2H), 6.98 (d, *J* = 8.8 Hz, 2H), 3.83 (s, 3H). ^1^HNMR data were in accordance with those reported in the literature [15].

### 3.4. General Procedure for Synthesis of Amidoximes ***1**–**19***

**Scheme 1:**
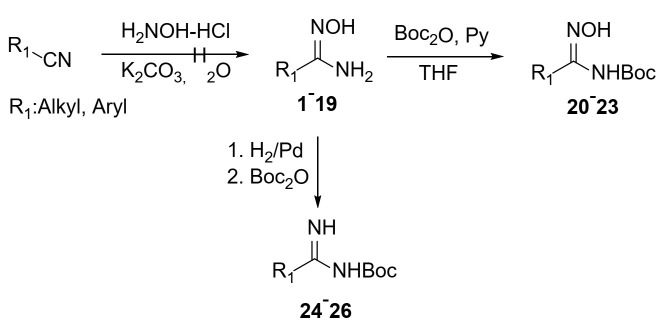
Synthesis of the studied compounds **1**–**26**.

**Figure 2:**
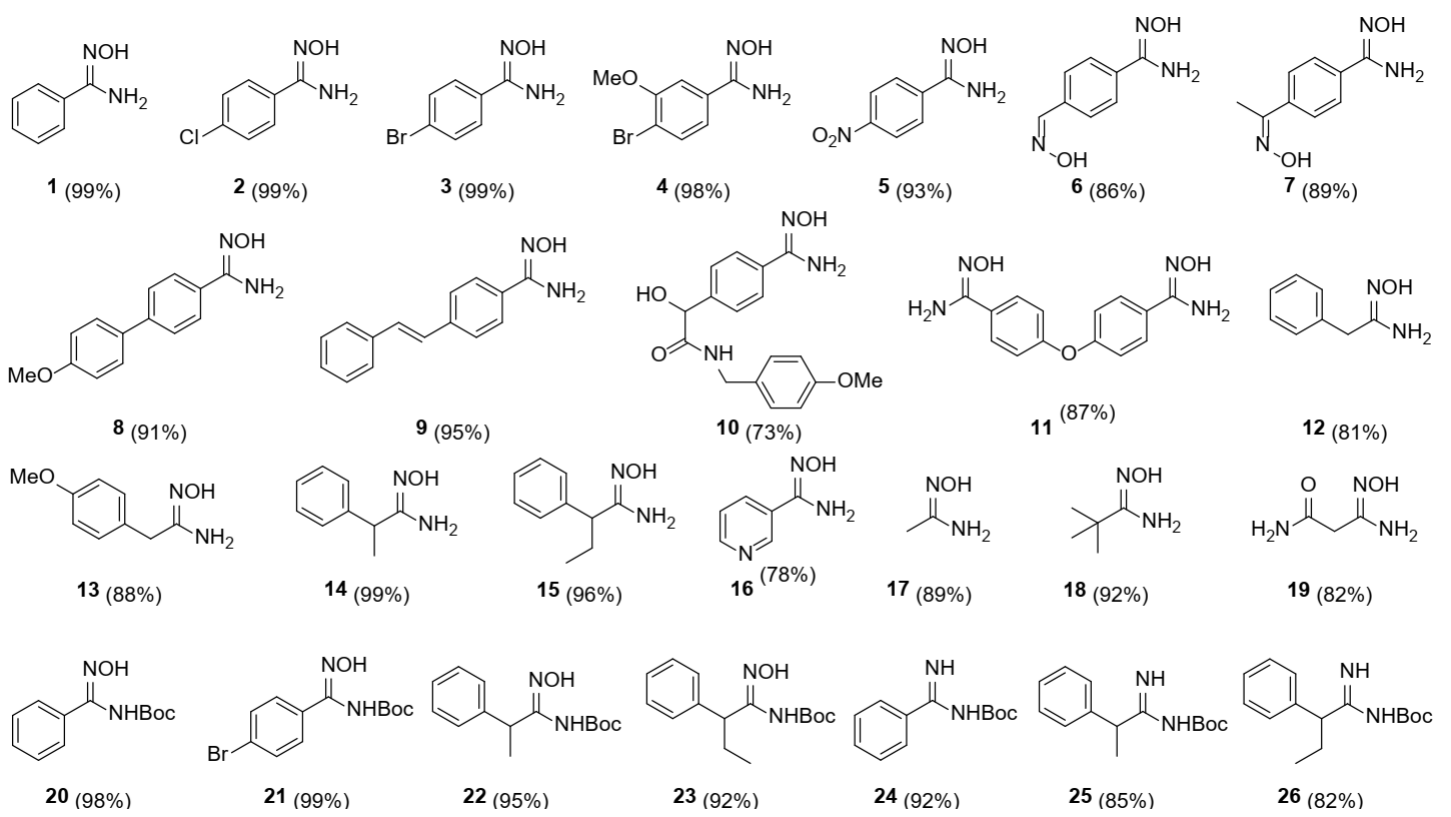
The chemical structures of the obtained amidine derivatives **1**–**26**. Yields are provided in brackets.

A solution of nitrile (2.5 mmol), hydroxylamine hydrochloride (695 mg, 10.0 mol), sodium carbonate (530 mg, 5.0 mol), water (6 mL) and ethanol (9 mL) was refluxed for 3 h. The reaction was allowed to cool, and the ethanol was removed under reduced pressure. The aqueous layer was extracted with ethyl acetate (3 × 10 mL); the combined organic fractions were dried over anhydrous Na_2_SO_4_ and the solvent removed under reduced pressure to afford the desired amidoximes **1**–**19**, which were sufficiently pure for use without further purification (Scheme 1, Figure 2).

#### 3.4.1. N’-Hydroxybenzimidamide (**1**)

Compound **1** was obtained according to General method with 99% yield (340 mg, 2.5 mmol) as white crystals; m.p. 70–71 °C (Lit. m.p. 67–69 °C) [16] ^1^H NMR (400 MHz, DMSO-d_6_) δ 9.59 (s, 1H), 7.73–7.58 (m, 2H), 7.41–7.22 (m, 3H), 5.75 (s, 2H); ^13^C NMR (100 MHz, DMSO-d_6_) δ 151.3, 133.8, 129.3, 128.5, 125.8. ^1^H and ^13^C NMR data were in accordance with those reported in the literature [17].

#### 3.4.2. 4-Chlorobenzamidoxime (**2**)

Compound **2** was obtained according to General method with 99% yield (422 mg, 2.5 mmol) as white crystals m.p. 130–131 °C (Lit. m.p. 128–130 °C) [18]; ^1^H NMR (400 MHz, DMSO-d_6_) δ 9.70 (s, 1H), 7.68 (d, *J* = 8.7 Hz, 2H), 7.41 (d, *J* = 8.6 Hz, 2H), 5.83 (s, 2H); ^13^C NMR (100 MHz, DMSO-d_6_) δ 150.4, 133.9, 128.5, 127.5. ^1^H and ^13^C NMR data were in accordance with those reported in the literature [19].

#### 3.4.3. p-Bromobenzamidoxime (**3**)

Compound **3** was obtained according to General method with 99% yield (372 mg, 2.5 mmol) as white crystals; m.p. 142–143 °C; (Lit. m.p. 140–141 °C) [20]; ^1^H NMR (400 MHz, DMSO-d_6_) δ 9.72 (s, 1H), 7.86–7.31 (m, 4H), 5.82 (s, 2H); ^13^C NMR (100 MHz, DMSO-d_6_) δ 150.5, 133.0, 131.5, 127.8, 122.5. ^1^H and ^13^C NMR data were in accordance with those reported in the literature [21].

#### 3.4.4. 4-Bromo-N’-hydroxy-3-methoxybenzimidamide (**4**)

Compound **4** was obtained according to General method with 98% yield (600 mg, 2.4 mmol) as white crystals, m.p. 128–129 °C; ^1^H NMR (400 MHz, DMSO-d_6_) δ 9.69 (s, 1H), 7.53 (d, *J* = 8.3 Hz, 1H), 7.34 (d, *J* = 1.9 Hz, 1H), 7.19 (dd, *J* = 8.3, 1.9 Hz, 1H), 5.86 (s, 2H), 3.85 (s, 3H); ^13^C NMR (100 MHz, DMSO-d_6_) δ 155.5, 150.5, 134.7, 132.9, 119.3, 111.5, 109.9, 56.6. Elemental analysis calculated for C_8_H_9_BrN_2_O_2_: C, 39.21%; H, 3.70%; N, 11.43%; found: C, 39.18%; H, 3.65%; N, 11.37%.

#### 3.4.5. N’-Hydroxy-4-nitrobenzimidamide (**5**)

Compound **5** was obtained according to General method with 93% yield (421 mg, 2.3 mmol) as white crystals; m.p. 185 °C (Lit. m.p. 188–190 °C) [21]; ^1^H NMR (400 MHz, DMSO-d_6_) δ 8.98 (s, 1H), 8.13 (d, *J* = 8.7 Hz, 2H), 7.52 (d, *J* = 8.7 Hz, 2H), 5.50 (s, 2H); ^13^C NMR (100 MHz, DMSO-d_6_) δ 151.5, 146.7, 130.4, 123.6. ^1^H and ^13^C NMR data were in accordance with those reported in the literature [22]

#### 3.4.6. N’-Hydroxy-4-((hydroxyimino)methyl)benzimidamide (**6**)

Compound **6** was obtained according to General method with 86% yield (385 mg, 2.1 mmol) as white crystals; m.p. 211–213 °C; ^1^H NMR (400 MHz, DMSO-d_6_) δ 11.25 (s, 1H), 9.68 (s, 1H), 8.12 (s, 1H), 7.68 (d, *J* = 8.4 Hz, 2H), 7.56 (d, *J* = 8.4 Hz, 2H), 5.79 (s, 2H); ^13^C NMR (100 MHz, DMSO-d_6_) δ 150.8, 148.2, 134.4, 133.9, 126.5, 126.0. ^1^H and ^13^C NMR data were in accordance with those reported in the literature [23].

#### 3.4.7. N’-Hydroxy-4- [N-hydroxyethanimidoyl]benzene-1-carboximidamide (**7**)

Compound **7** was obtained according to General method with 89% yield (430 mg, 2.2 mmol) as white crystals; m.p. 192–193 °C; ^1^H NMR (400 MHz, DMSO-d_6_) δ 11.20 (s, 1H), 9.65 (s, 1H), 7.71–7.59 (m, 4H), 5.78 (s, 2H), 2.14 (s, 3H); ^13^C NMR (100 MHz, DMSO-d_6_) δ 153.0, 150.9, 137.7, 125.7, 125.6, 11.8. ^1^H and ^13^C NMR data were in accordance with those reported in the literature [24].

#### 3.4.8. 4-(4-Methoxyphenyl)phenyl]-nitrosomethanamine (**8**)

Compound **8** was obtained according to General method with 91% yield (551 mg, 2.3 mmol) as white crystals; m.p. 143–145 °C; ^1^H NMR (400 MHz, DMSO-_d6_) δ 9.65 (s, 1H), 7.73–7.58 (m, 7H), 7.01–6.98 (m, 2H), 5.78 (s, 2H), 3.76 (s, 3H); ^13^C NMR (100 MHz, DMSO-_d6_) δ 159.5, 151.1, 132.2, 132.0, 128.6, 128.4, 128.1, 126.3, 126.2, 126.2, 114.9, 114.8, 55.6. HR-MS (ESI) (M + H)^+^ m/z calculated for C_14_H_15_N_2_O_2_: 243.1134; found: 243.1132. Elemental analysis calculated for C_14_H_14_N_2_O2: C, 69.41%; H, 5.82%; N, 11.56%; found: C, 69.24%; H, 5.91%; N, 11.38%.

#### 3.4.9. N’-Hydroxy-4- [(E)-2-phenylethenyl]benzene-1-carboximidamide (**9**)

Compound **9** was obtained according to General method with 95% yield (566 mg, 2.3 mmol) as white crystals, m.p. 133–134 °C; ^1^H NMR (400 MHz, DMSO-d_6_) δ 9.63 (s, 1H), 7.77–7.53 (m, 6H), 7.36 (t, *J* = 7.6 Hz, 2H), 7.32–7.14 (m, 3H), 5.77 (s, 2H); ^13^C NMR (100 MHz, DMSO-d_6_) δ 151.0, 138.0, 137.4, 132.8, 129.1, 128.3, 126.9, 126.6, 126.0, 117.4, 109.0. Elemental analysis calculated for C_15_H_14_N_2_O: C, 75.61%; H, 5.92%; N, 11.76%; found: C, 75.55%; H, 5.82%; N, 11.64%.

#### 3.4.10. 2-{4-[1-Amino-2-hydroxyethenyl]phenyl}-2-hydroxy-N-[(4-methoxyphenyl)methyl]acetamide (**10**)

Compound **10** was obtained according to General method with 73% yield (601 mg, 1.8 mmol) as white crystals m.p. 151–152 °C; ^1^H NMR (400 MHz, DMSO-d_6_) δ 8.49 (s, 1H), 7.78 (d, *J* = 8.0 Hz, 2H), 7.60 (d, *J* = 8.2 Hz, 2H), 7.10 (d, *J* = 8.0 Hz, 2H), 6.82 (d, *J* = 8.1 Hz, 2H), 6.42 (s, 1H), 5.05 (s, 1H), 4.17 (s, 2H), 3.69 (s, 3H). HR-MS (ESI) (M + H)^+^ m/z calculated for C_17_H_20_N_3_O_4_: 329.1495; found: 329.1491. Elemental analysis calculated for C_17_H_19_N_3_O_4_: C, 62.00%; H, 5.81%; N, 12.76%; found: C, 61.89%; H, 5.97%; N, 12.54%.

#### 3.4.11. 4,4’-Oxy-bis-benzamide oxime (**11**)

Compound **11** was obtained according to General method with 87% yield (623 mg, 2.2 mmol) as white crystals; m.p. 194–195 °C (Lit. m.p. 193–194 °C) [25]; ^1^H NMR (400 MHz, DMSO-d_6_) δ 7.66 (d, *J* = 8.2 Hz, 4H), 6.99 (d, *J* = 8.2 Hz, 4H), 5.74 (s, 4H); ^13^C NMR (100 MHz, DMSO-d_6_) δ 157.4, 151.0, 130.2, 129.1, 127.8, 127.7, 119.3, 118.8, 118.7, 118.1. Elemental analysis calculated for C_14_H_14_N_4_O_3_: C, 58.74%; H, 4.93%; N, 19.57%; found: C, 58.66%; H, 4.88%; N, 19.45%.

#### 3.4.12. Benzylamidoxime (**12**)

Compound **12** was obtained according to General method with 81% yield (304 mg, 2.0 mmol) as white crystals; m.p. 65–66 °C (Lit. 67–68 °C) [26]; ^1^H NMR (400 MHz, DMSO-d_6_) δ 8.85 (s, 1H), 7.30–7.11 (m, 5H), 5.33 (s, 2H), 3.25 (s, 2H); ^13^C NMR (100 MHz, DMSO-d_6_) δ 152.3, 138.4, 129.1, 128.5, 126.6, 37.6. ^1^H and ^13^C NMR data were in accordance with those reported in the literature [27].

#### 3.4.13. N’-Hydroxy-2-(4-methoxyphenyl)ethanimidamide (**13**)

Compound **13** was obtained according to General method with 88% yield (396 mg, 2.2 mmol) as white crystals; m.p. 111–112 °C (Lit. m.p. 110–111 °C) [28]; ^1^H NMR (400 MHz, DMSO-d_6_) δ 8.80 (s, 1H), 7.16 (d, *J* = 8.8 Hz, 2H), 6.82 (d, *J* = 8.6 Hz, 2H), 5.27 (s, 2H), 3.70 (s, 3H), 3.16 (s, 2H); ^13^C NMR (100 MHz, DMSO-d_6_) δ 158.2, 152.7, 130.3, 130.1, 114.0, 55.4, 36.7. ^1^H and ^13^C NMR data were in accordance with those reported in the literature [29].

#### 3.4.14. N’-Hydroxy-2-phenylpropanimidamide (**14**)

Compound **14** was obtained according to General method with 99% yield (406 mg, 2.5 mmol) as white crystals; m.p. 138–139 °C; ^1^H NMR (400 MHz, CDCl_3_) δ 7.40–7.24 (m, 5H), 6.32 (s, 1H), 4.52 (s, 2H), 3.67 (q, *J* = 7.2 Hz, 1H), 1.51 (d, *J* = 7.2 Hz, 3H); ^13^C NMR (100 MHz, CDCl_3_) δ 141.3, 128.8, 127.3, 41.6, 18.0. Elemental analysis calculated for C_9_H_12_N_2_O: C, 65.83%; H, 7.37%; N, 17.06%; found: C, 65.77%; H, 7.32%; N, 16.98%.

#### 3.4.15. N-Hydroxy-2-phenyl-butyrimidamide (**15**)

Compound **15** obtained according to General method with 96% yield (428 mg, 2.3 mmol) as white crystals 67–68 °C; ^1^H NMR (400 MHz, CDCl_3_) δ 7.35–7.24 (m, 5H), 4.42 (s, 2H), 3.32 (t, *J* = 7.7 Hz, 1H), 2.07–1.80 (m, 2H), 0.91 (t, *J* = 7.3 Hz, 3H); ^13^C NMR (100 MHz, CDCl_3_) δ 155.2, 140.4, 128.7, 127.8, 127.1, 49.5, 24.8, 12.1. Elemental analysis calculated for C_10_H_14_N_2_O: C, 67.39%; H, 7.92%; N, 15.72%; found: C, 67.28%; H, 7.84%; N, 15.55%.

#### 3.4.16. Nicotinamide oxime (**16**)

Compound **16** was obtained according to General method with 78% yield (267 mg, 1.9 mmol) as white crystals; m.p. 108–110 °C (Lit. 107–109 °C) [30]; ^1^H NMR (400 MHz, DMSO-d_6_) δ 9.80 (s, 1H), 8.84 (dd, *J* = 2.3, 1.0 Hz, 1H), 8.54 (dd, *J* = 4.8, 1.7 Hz, 1H), 7.99 (d, *J* = 8.0 Hz, 1H), 7.55–7.19 (m, 1H), 5.94 (s, 2H); ^13^C NMR (100 MHz, DMSO-d_6_) δ 150.2, 149.4, 147.0, 133.2, 129.5, 123.6. ^1^H and ^13^C NMR data were in accordance with those reported in the literature [31].

#### 3.4.17. Acetamide oxime (**17**)

Compound **17** was obtained according to General method with 89% yield (165 mg, 2.2 mmol) as white crystals; m.p. 135–136 °C (Lit. m.p. 136–137 °C) [32]; ^1^H NMR (400 MHz, DMSO-d_6_) δ 8.64 (s, 1H), 5.37 (s, 2H), 1.61 (s, 3H); ^13^C NMR (100 MHz, DMSO-d_6_) δ 150.3, 17.0. ^1^H and ^13^C NMR data were in accordance with those reported in the literature [33].

#### 3.4.18. N’-Hydroxypivalimidamide (**18**)

Compound **18** was obtained according to General method with 92% yield (267 mg, 2.3 mmol) as white crystals, m.p. 112–113 °C (Lit. m.p. 115–116 °C) [34]; ^1^H NMR (400 MHz, CDCl_3_) δ 8.47 (s, 1H), 4.57 (s, 2H), 1.17 (s, 9H); ^13^C NMR (100 MHz, CDCl_3_) δ 159.6, 34.5, 27.8. ^1^H and ^13^C NMR data were in accordance with those reported in the literature [35].

#### 3.4.19. β-Amino-β-oximinopropioamide (**19**)

Compound **19** was obtained according to General method with 82% yield (240 mg, 2.0 mmol) as white crystal; m.p. 154–155 °C (Lit. m.p. 159 °C) [36]; ^1^H NMR (400 MHz, DMSO-d_6_) δ 8.94 (s, 1H), 7.32 (s, 1H), 6.93 (s, 1H), 5.36 (s, 2H), 2.78 (s, 2H); ^13^C NMR (100 MHz, DMSO-d_6_) δ 149.4, 38.3. ^1^H and ^13^C NMR data were in accordance with those reported in the literature [37].

### 3.5. General Procedure for Synthesis of Compounds ***20**–**23***

Amidoxime (1 mmol) was dissolved in a mixture of deionized water (20 mL), 2 mL NaOH (2N) and THF (20 mL). The solution was cooled to 0 °C and di-*tert*-butyl dicarbonate (Boc-anhydride, Boc_2_O) (250 mg, 1.1 mmol) was added dropwise. The resulted mixture was stirred for 4 h at room temperature. The reaction mixture was then concentrated in vacuo, extracted with EtOAc (3 × 15 mL) and washed with H_2_O (3 × 20 mL). The organic phase was dried with sodium sulfate and concentrated in vacuo. The crude product was purified by column chromatography with silica gel (hexanes/EtOAc) to afford corresponding products **20**–**23** (Scheme 1, Figure 2)

#### 3.5.1. Tert-Butyl [(Z)-(hydroxyimino)(phenyl)methyl]carbamate (**20**)

Compound **20** was obtained according to General method with 98% yield (231 mg, 0.98 mmol) as white crystals; m.p. 110–111 °C; ^1^H NMR (400 MHz, CDCl_3_) δ 7.74–7.63 (m, 2H), 7.50–7.33 (m, 3H), 5.08 (s, 2H), 1.54 (s, 9H); ^13^C NMR (100 MHz, CDCl_3_) δ 155.7, 131.1, 130.8, 128.6, 83.0, 27.8. Elemental analysis calculated for C_12_H_16_N_2_O_3_: C, 61.00%; H, 6.83%; N, 11.86%; found: C, 60.95%; H, 6.87%; N, 11.75%.

#### 3.5.2. Tert-Butyl [(4-bromophenyl)(imino)methyl]carbamate (**21**)

Compound **21** was obtained according to General method with 99% yield (248 mg, 0.99 mmol) as white crystals, m.p. 118–119 °C; ^1^H NMR (400 MHz, CDCl_3_) δ 7.62–7.45 (m, 4H), 5.08 (s, 2H), 1.54 (s, 9H); ^13^C NMR (100 MHz, CDCl_3_) δ 154.8, 151.9, 131.8, 130.0, 128.1, 125.3, 83.3, 27.7. Elemental analysis calculated for C_12_H_15_BrN_2_O_3_: C, 45.73%; H, 4.80%; N, 8.89%; found: C, 45.83%; H, 4.77%; N, 8.83%.

#### 3.5.3. Tert-Butyl [N-hydroxy-2-phenylpropanimidoyl]carbamate (**22**)

Compound **22** was obtained according to General method with 95% yield (251 mg, 0.95 mmol) as white crystals; m.p. 141–142 °C; ^1^H NMR (400 MHz, CDCl_3_) δ 7.34–7.21 (m, 5H), 4.55 (s, 2H), 3.73 (q, *J* = 7.3 Hz, 1H), 1.53 (d, *J* = 7.3 Hz, 3H), 1.49 (s, 9H); ^13^C NMR (100 MHz, CDCl_3_) δ 159.9, 152.1, 140.5, 128.8, 127.3, 41.3, 27.8, 17.7. HR-MS (ESI) (M + H)^+^ m/z calculated for C_14_H_21_N_2_O_3_: 265.1546; found: 265.1545. Elemental analysis calculated for C_14_H_20_N_2_O_3_: C, 63.62%; H, 7.63%; N, 10.60%; found: C, 63.49%; H, 7.72%; N, 10.45%.

#### 3.5.4. Tert-Butyl [N-hydroxy-2-phenylbutanimidoyl]carbamate (**23**)

Compound **23** was obtained according to General method with 92% yield (256 mg, 0.92 mmol) as white crystals m.p. 149–151 °C; ^1^H NMR (400 MHz, CDCl_3_) δ 7.37–7.24 (m, 5H), 4.55 (s, 1H), 3.44 (td, *J* = 7.9, 0.9 Hz, 1H), 2.06–1.87 (m, 2H), 1.49 (s, 9H), 0.92 (td, *J* = 7.3, 0.8 Hz, 3H); ^13^C NMR (100 MHz, CDCl_3_) δ 159.0, 146.7, 139.4, 128.7, 128.7, 127.7, 127.2, 85.0, 82.4, 48.9, 27.3, 24.7, 11.9. HR-MS (ESI) (M + H)^+^ m/z calculated for C_15_H_23_N_2_O_3_: 279.1703; found: 279.1701. Elemental analysis calculated for C_15_H_22_N_2_O_3_: C, 64.73%; H, 7.97%; N, 10.06%; found: C, 64.61%; H, 7.85%; N, 9.92%.

### 3.6. General Procedure for Synthesis of Amidines ***24**–**26***

This reaction was carried out according to the procedure described by Judkins et al., [38] to afford corresponding products **24**–**26** (Scheme 1, Figure 2)

#### 3.6.1. Tert-Butyl [imino(phenyl)methyl]carbamate (**24**)

Compound **24** was obtained according to General method with 92% yield (203 mg, 0.92 mmol) as white crystals; m.p. 101–102 °C (Lit. 102–103 °C) [39]; ^1^H NMR (400 MHz, CDCl_3_) δ 7.83 (ddt, *J* = 7.2, 3.4, 1.3 Hz, 2H), 7.55–7.46 (m, 1H), 7.46–7.30 (m, 2H), 1.53 (s, 9H); ^13^C NMR (100 MHz, CDCl_3_) δ 128.5, 127.1, 28.1. ^1^H and ^13^C NMR data were in accordance with those reported in the literature [40].

#### 3.6.2. Tert-Butyl (2-phenylpropanimidoyl)carbamate (**25**)

Compound **25** was obtained according to General method with 85% yield (211 mg, 0.85 mmol) as white crystals; m.p. 174–176 °C; ^1^H NMR (400 MHz, CDCl_3_) δ 7.40–7.15 (m, 5H), 3.72 (q, *J* = 7.3 Hz, 1H), 1.55 (d, *J* = 7.3 Hz, 3H), 1.46 (s, 9H); ^13^C NMR (100 MHz, CDCl_3_) δ 140.1, 128.9, 127.8, 127.6, 79.5, 47.0, 28.1, 18.6. HR-MS (ESI) (M + H)^+^ m/z calculated for C_14_H_21_N_2_O_2_: 249.1597; found: 249.1595. Elemental analysis calculated for C_14_H_20_N_2_O_2_: C, 67.72%; H, 8.12%; N, 11.28%; found: C, 67.69%; H, 8.02%; N, 11.08%.

#### 3.6.3. Tert-Butyl (2-phenylbutanimidoyl)carbamate (**26**)

Compound **26** was obtained according to General method with 82% yield (215 mg, 0.82 mmol) as white crystals; m.p. 164–165 °C; ^1^H NMR (400 MHz, CDCl_3_) δ 7.41–7.22 (m, 5H), 3.45 (dd, *J* = 9.5, 6.0 Hz, 1H), 2.21 (ddd, *J* = 13.6, 7.4, 6.0 Hz, 1H), 1.90 (ddd, *J* = 13.8, 9.6, 7.2 Hz, 1H), 1.48 (s, 9H), 0.87 (t, *J* = 7.3 Hz, 3H); ^13^C NMR (100 MHz, CDCl_3_) δ 138.6, 129.0, 128.3, 127.6, 79.6, 54.9, 28.1, 26.0, 12.2. HR-MS (ESI) (M + H)^+^ m/z calculated for C_15_H_23_N_2_O_2_: 263.1754; found: 263.1752. Elemental analysis calculated for C_15_H_22_N_2_O_2_: C, 68.67%; H, 8.45%; N, 10.68%; found: C, 68.48%; H, 8.39%; N, 10.55%.

## 4. Microorganisms and Media

The microbiological analysis procedures have been carefully described in the literature citations [41,42,43,44,45,46,47,48,49].

## 5. Results and Discussion

### 5.1. Chemistry

The amidoximes **1**–**19** (Figure 2) were chosen for another studies. For comparison, we prepared Boc-protected amidoximes **20**–**23** and amidines **24**–**26**, which were obtained via reduction of parental amidoximes (Scheme 1). Amidoximes were synthesized in high yields according to general procedure consisting of addition of hydroxylamine to nitrile group in alcohol solution under reflux and sodium carbonate added as inorganic base. Furthermore, the obtained amidoximes were efficiently reduced to amidines under hydrogen atmosphere in the presence of palladium catalyst in environment of acetic acid, acetic acid anhydride and ethanol [50]. Due to the presence of free amine group in both amidines and amidoximes, they can undergo various chemical reactions of *N*-alkylation and *N*-acylation. One of them is Boc-protection, often used in organic synthesis to protect amino and imino group. To date, Boc-protected amidoximes were not studied for biological activity thus it still remains unknown.

In our studies not only amidoximes and Boc-protected amidoximes were tested, but also Boc-derived amidines. Studied compounds are shown in Figure 2. The structures of all compounds were confirmed using NMR and mass spectroscopy. Melting points and spectral data of **1**–**26** remained in agreement with the literature data. The characterization data of the synthetized compounds **1**–**26** are presented in the experimental part.

### 5.2. Cytotoxic Studies of the Library of Amidoxime Derivatives ***1**–**26***

**Figure 3:**
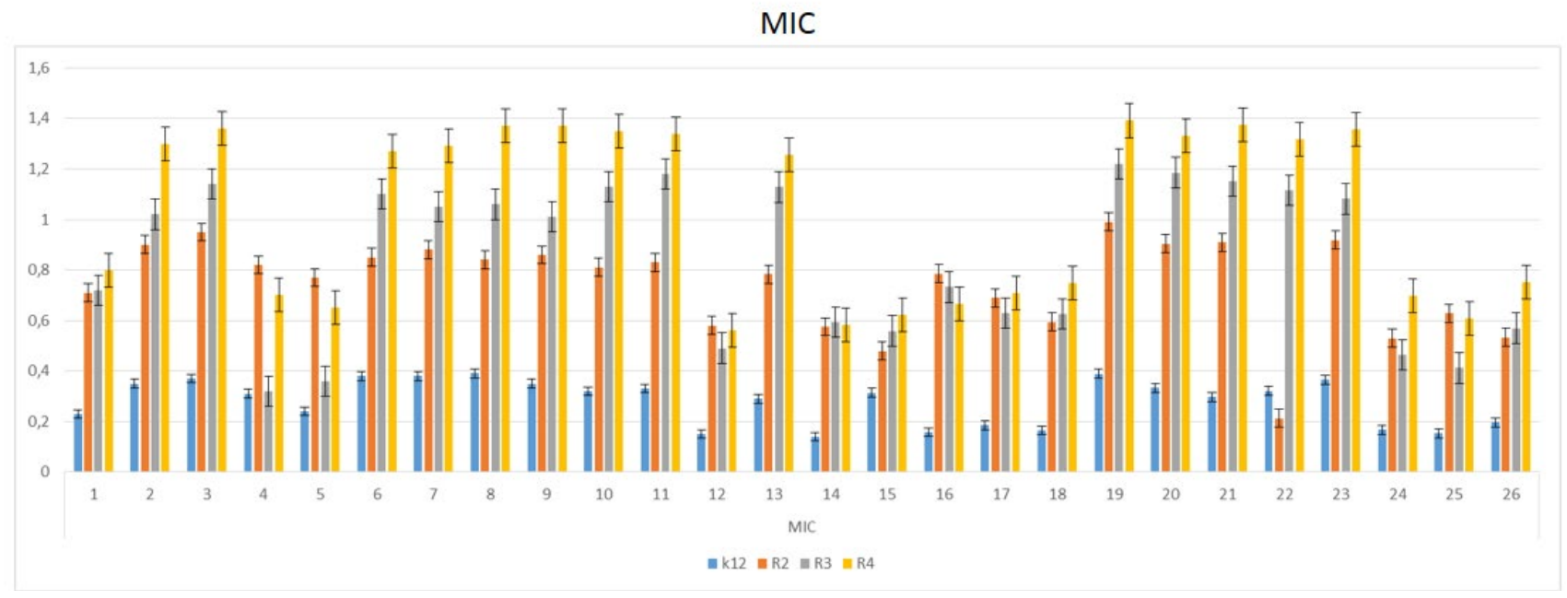
Minimum inhibitory concentration (MIC) of the amidoximes in model bacterial strains. The *x*-axis features amidoxime derivatives **1**–**26** used sequentially. The *y*-axis shows the MIC value in µg/mL^−1^. Investigated strains of *E. coli* K12 as control (blue), R2 strains (orange), R3 strain (grey), and R4 strain (yellow). The *y*-axis shows the MBC value in µg/mL^−1^. The order in which the compounds were applied to the plate are shown in Supplementary Materials Figure S1. Each experiment was performed independently in 3 replications (*n* = 3).

**Figure 4:**
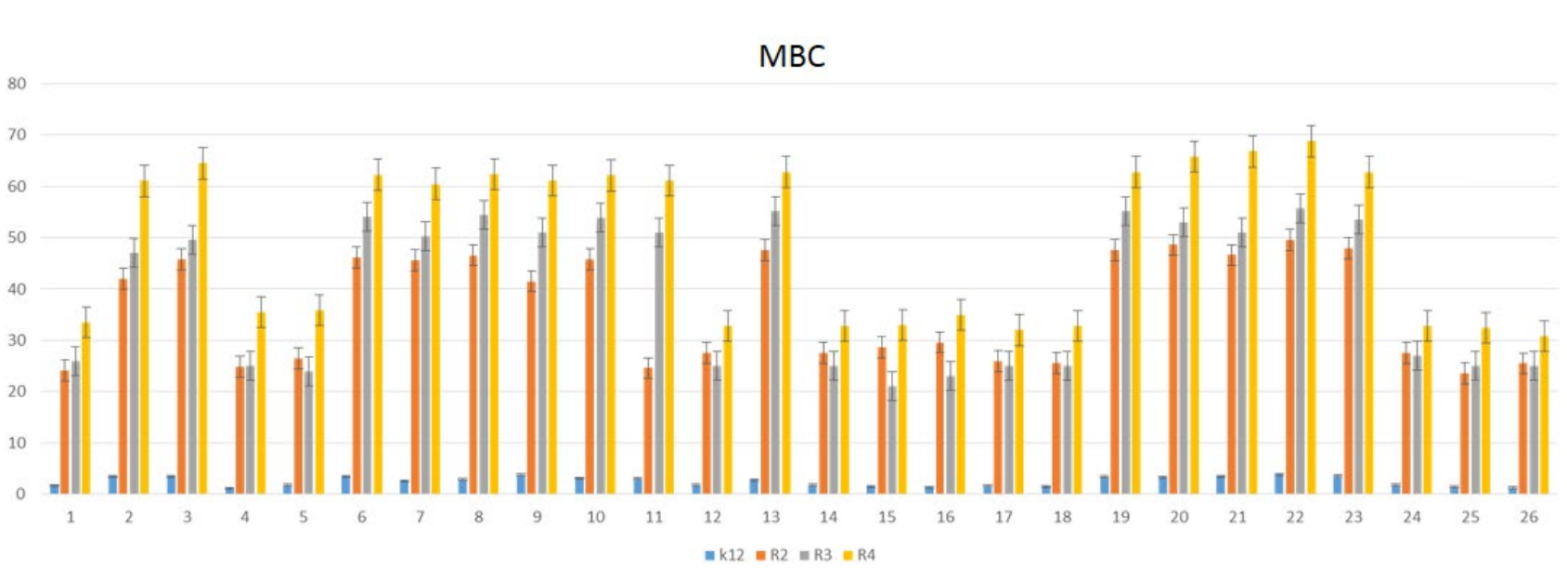
MBC of the amidoxime derivatives **1**–**26** in model bacterial strains. On the *x*-axis, **26** compounds were used sequentially. The *y*-axis shows the MBC value in µg/mL^−1^ (see Supplementary Materials). Each experiment was performed independently in 3 replications (*n* = 3).

**Figure 5:**
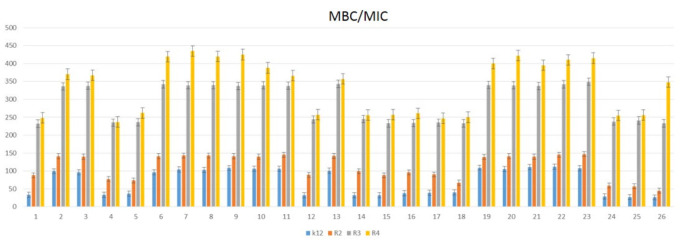
MBC/MIC of the amidoxime derivatives **1**–**26** in model bacterial strains. On the *x*-axis, compounds **1**–**26** are used sequentially. The *y*-axis shows the MBC/MIC value in µg/mL^−1^ (see Supplementary Materials). Each experiment was performed independently in 3 replications (*n* = 3).

In general, the obtained results depict that the all studied amidoximes have an inhibitory effect on each bacterial model studied. Varied inhibitory activity was noted depending on the nature of the substituent in the aromatic ring of the tested compounds **1**–**11**. Interestingly, the benzylamidoxime derivatives **12**–**15** exhibit lower minimal inhibitory concentration compared to the benzamidoximes **1**–**11**. It should be noted that in the case of benzamidoximes **4** and **5**, we observed the selectivity towards the R3 strain, while benzylamide derivative **12** shows selectivity and increased inhibitory activity towards the strain R2. The metoxy group introduced into aromatic ring has an unfavorable impact on the activity of benzylamidoxime **13**, while it increases the activity of benzamidoxime **4**. The introduction of the Boc-group into the studied compound **20**–**23** reduces their inhibiting activity toward all tested strains. We noted that the removal of the hydroxyl group by reduction of the corresponding oximes is associated with a decrease in inhibitory activity of compounds **24**–**26**. Interestingly, we have shown a great impact of the methyl group on the activity of the studied compounds. Oxime **22** exhibited higher inhibiting activity than its analogue **23**, which is clearly visible on the basis of the data obtained for the analyzed strains (Figure 3, Figure 4 and Figure 5 and Table 1).

### 5.3. The Analysis of Bacterial DNA Isolated from E. coli R2–R4 Strains Modified with Amidoxim

**Figure 6:**
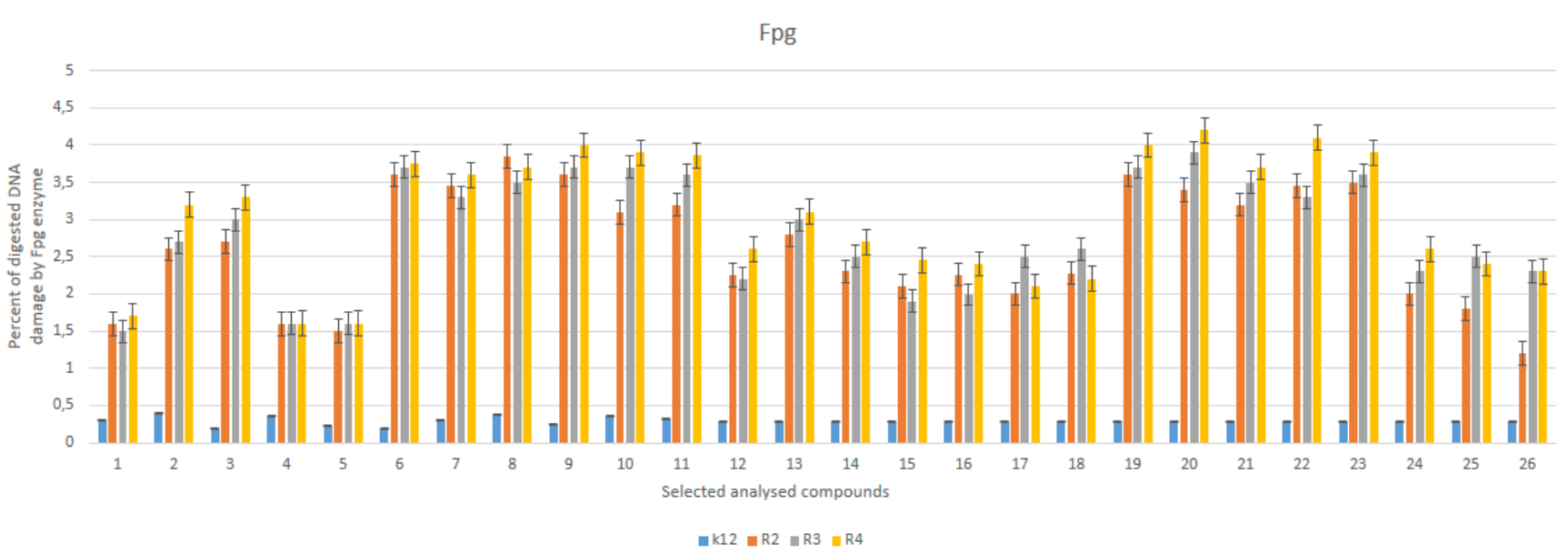
Percentage of plasmid DNA recognized by Fpg enzyme (*y*-axis) with model bacterial, K12, and R2–R4 strains (*x*-axis). The compounds numbered **2**, **3**, **6**, **7**, **8**, **9**, **10**, **11**, **13**, **19**, **20**, **21**, **22**, **23** were statistically significant at <0.05 * (see Table 1). Each experiment was performed independently in 3 replications (*n* = 3).

Among all analyzed compounds, the highest toxicity was observed for compounds marked as **2**, **3**, **6**, **7**, **8**, **9**, **10**, **11**, **13**, **19**, **20**, **21**, **22**, **23**. The selected compounds used were chosen to modify model *E. coli* strains and, additionally, were digested with Fpg protein from the group of repair glycosylases, which is a marker of oxidative stress [41,42,43,44,45,46,47]. As in previous studies on various types of compounds, we also wanted to observe the effect of modification on the magnitude of oxidative damage, which should be seen as extending strands compared to unmodified forms having the three forms ccc, oc and linear. What has been observed in previous studies [41,42,43,44,45,46,47,48,49]. The results of bacterial DNA modified with amidoxime is shown in Figure 6 (with the action of Fpg), and in Supplementary Materials Figures S1 and S2.

**Figure 7:**
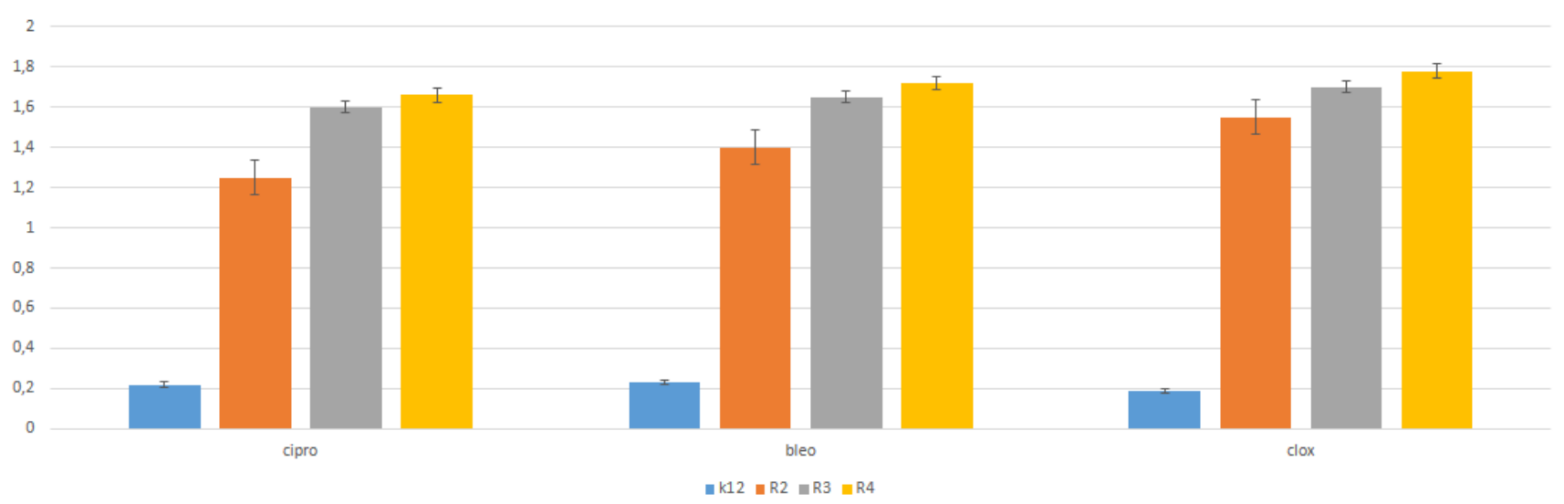
Examples of MIC with model bacterial strains K12, R2, R3, and R4 for studying antibiotics ciprofloxacin, bleomycin, and mechanism. The *x*-axis features antibiotics used sequentially. The *y*-axis features the MIC value in µg/mL^−1^. Each experiment was performed independently in 3 replications (*n* = 3).

**Figure 8:**
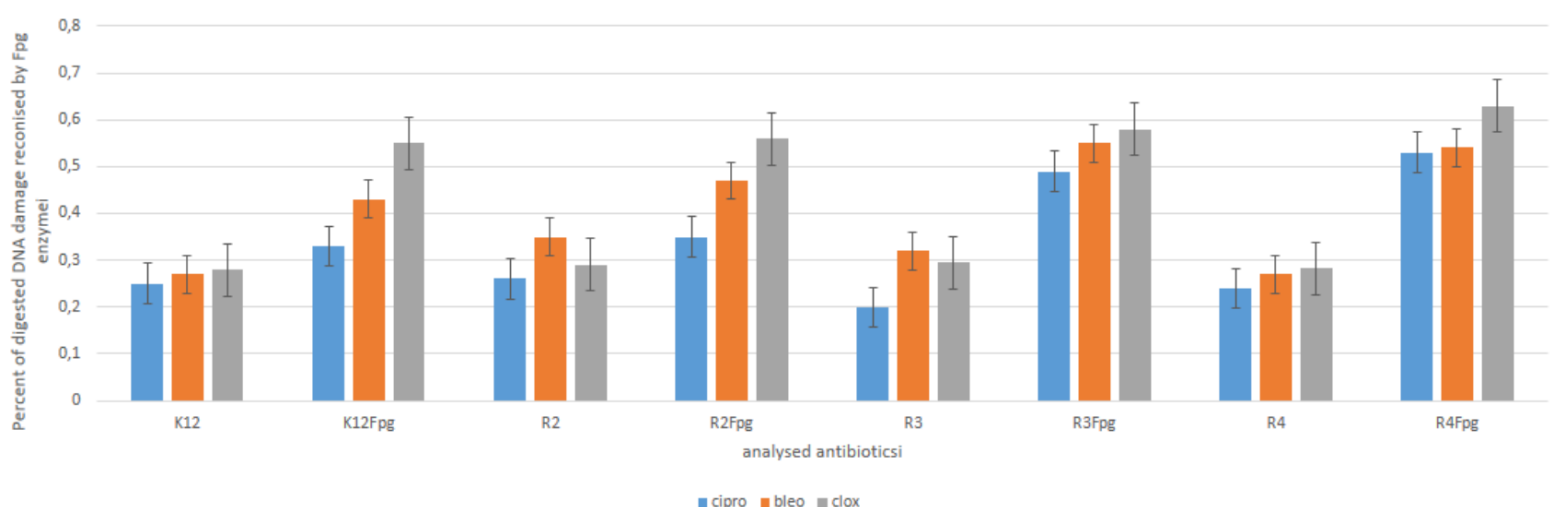
Percentage of bacterial DNA recognized by Fpg enzyme in model bacterial strains after ciprofloxacin, bleomycin, and cloxacillin treatment. The compounds were statistically significant at *p* < 0.05. Each experiment was performed independently in 3 replications (*n* = 3).

The changes in the main topological forms of the plasmid: ccc, oc and linear were observed in DNA isolated from model strains and digested with Fpg protein. About 4% of oxidative damage was identified after digestion with the Fpg protein, which indicates that the analyzed compounds damage bacterial DNA very strongly due to the oxidative stress induced by them in the cell [41,42,43,44,45,46,47,48,49]. Our observations indicate that the length of the alkyl chain of peptidomimetics may determine the toxicity to some *E. coli* R4 strains, as evidenced by the MIC and MBC values [50]. The obtained results were also statistically significant at the level of *p* < 0.05. (Figure 6). The obtained results indicate that the studied compounds can potentially be used as “substitutes for” commonly applied antibiotics—Figure 7 and Figure 8.

In the analyzed bacterial strains, after DNA isolation from them and after a modification with antibiotics and digestion with Fpg protein, no significant changes in topological forms were observed (Supplementary Materials, Figure S3). This suggests that modifications with antibiotics are less recognizable by the Fpg protein than the modifications introduced into the bacterial DNA by the analyzed compounds (Figure 8). Probably the modifications of the antibiotics used in the bacterial DNA do not cause significant changes in the topological principles caused by the specific bacterial glycosylase to which the Fpg protein belongs.

Large modifications of plasmid DNA were observed for compounds **2**, **3**, **6**, **7**, **8**, **9**, **10**, **11**, **13**, **19**, **20**, **21**, **22**, **23**. Modifications with antibiotics were smaller and not as clear as in the case of the analyzed amidoximes. The sensitivity of *E. coli* strains to the cytotoxic effect of the compounds used and after Fpg protein digestion was as follows: R4 > R2 > R3 > K12 and this effect was very similar to our previous studies [41,42,43,44,45,46,47,48,49]. This indicates a very high cytotoxicity of the analyzed amidoxime derivatives towards bacterial DNA, probably resulting from the modification of the components of the bacterial membrane and the LPS contained in it, which may induce specific enzymes from the group of topoisomerases and helicases, destabilizing the structure of the exposed DNA bases. A stabilization of the complex that regulates these enzymes is perhaps necessary for cell survival. Blocking these enzymes inhibits DNA replication and rewriting, which can affect its total amount.

## 6. Conclusions

Obtained results revealed that carefully designed amidoxime derivatives may constitute a new potential source of innovative, cheap, substitutes for antibiotics against various types of bacterial microorganisms (LPS). We focused on the structure-activity relationship of compounds with an amidoximes scaffold. The obtained results showed a strong influence of the activity of all 26 compounds analyzed on the values of MIC and MBC as well as MBC/MIC for various strains of *E. coli* R2–R4 and K12. Based on the analysis of the above tests, 14 compounds (2, 3, 6, 7, 8, 9, 10, 11, 13, 19, 20, 21, 22, 23) were selected for further research. For selected compounds repair activity was compared using the Fpg-glycosylase protein of the BER pathway (base excision repair), which is consistent with our research hypothesis. The above results are very important for research on the mechanism of cytotoxic action of new compounds as innovative and safe drugs based on amidoxime derivatives, which may lead to the destruction of the bacterial cell membrane by changing its surface charge and may play a significant role in changing its electrokinetic potential expressing with the reversal of loads. A special effect was observed for compounds 2, 3, 6, 7, 8, 9, 10, 11, 13, 19, 20, 21, 22, 23, which showed certain MIC values and MBC/MIC ratios. Compounds no. 7 and 21 showed super-selectivity in all analyzed bacterial strains, even differentiating the cytotoxicity in the K12 strain. It should be noted that in the case of benzamidoximes 4 and 5, the selectivity towards the R3 strain was observed, while benzylamide derivative 12 shows selectivity and increased inhibitory activity towards the strain R2. The reported compounds may be highly specific for pathogenic *E. coli* strains on the basis of the model strains used. In the future, cytotoxicity studies will also be conducted using various cell lines and cultures to assess the biocompatibility of test compounds for active peptidomimetics

## Figures and Tables

**Table 1:** Statistical analysis of all analyzed compounds by MIC, MBC, and MBC/MIC; <0.05 *, <0.01 **, <0.001 ***.

No. of Samples	2	3	6	7, 8, 9	10, 11	13, 19	20, 21	22, 23	Type of Test
K12	*	*	*	**	*	*	***	***	MIC
R2	*	*	*	**	*	*	***	***	MIC
R3	*	*	*	**	*	*	***	***	MIC
R4	*	*	*	**	*	*	***	***	MIC
K12	*	*	**	*	**	*	**	**	MBC
R2	**	*	**	*	**	*	**	**	MBC
R3	**	*	**	*	**	*	**	**	MBC
R4	**	*	**	*	**	*	**	**	MBC
K12	*	*	*	*	*	*	***	***	MBC/MIC
R2	*	*	*	*	*	*	***	***	MBC/MIC
R3	*	*	*	*	*	*	***	***	MBC/MIC
R4	*	*	*	*	*	*	***	***	MBC/MIC

## Data Availability

Upon request of those interested.

## Supplementary Materials

The following are available online at https://www.mdpi.com/article/10.3390/ma14247577/s1. Figure S1: Examples of MIC and MBC on microplates with different concentration of studied compounds (mg L^−1^). Figure S2: Example of an agarose gel electrophoresis separation of isolated plasmids DNA from R4 strains modified with selected coumarin derivatives. Figure S3: Example of an agarose gel electrophoresis separation of isolated plasmids DNA from R4 strains modified with antibiotics: cloxacillin, ciprofloxacin, and bleomycin digested (or not) with repair enzymes Fpg. ^1^H and ^13^C NMR spectra of compounds **1**–**26**.

## Abbreviations

MIC	minimum inhibitory concentration
MBC	minimum bactericidal concentration
Oc	open circle
Ccc	covalently closed circle
BER	base excision repair
Fpg	DNA-formamidopyrimidine glycosylase
