# Supplementary material for: The Synthesis and Evaluation of Amidoximes as Cytotoxic Agents on Model Bacterial E. coli Strains
Source: Materials (Basel). 2021 Dec 9;14(24):7577. doi: 10.3390/ma14247577 (PMC8708467; doi:10.3390/ma14247577)
Supplement: Supplementary file 1 [file materials-14-07577-s001.zip › materials-1492699-supplementary.pdf]

materials

MDPI

Supplementary Materials

The Synthesis and Evaluation of Amidoximes as Cytotoxic Agents on Model Bacterial *E. coli* Strains

Jan Samsonowicz-Górski [1,†], Paweł Kowalczyk [2,*,†], Dominik Koszelewski [1,*], Anna Brodzka [1], Mateusz Szymczak [3], Karol Kramkowski [4] and Ryszard Ostaszewski [1]

1 Institute of Organic Chemistry PAS, Kasprzaka 44/52, 01-224 Warsaw, Poland; jan.samsonowicz-gorski.stud@pw.edu.pl (J.S.-G.); anna.brodzka@icho.edu.pl (A.B.); ryszard.ostaszewski@icho.edu.pl (R.O.)
2 Department of Animal Nutrition, The Kielanowski Institute of Animal Physiology and Nutrition, Polish Academy of Sciences, Instytucka 3, 05-110 Jabłonna, Poland
3 Department of Molecular Virology, Institute of Microbiology, Faculty of Biology, University of Warsaw, Miecznikowa 1, 02-096 Warsaw, Poland; mszymczak@biol.uw.edu.pl
4 Department of Physical Chemistry, Medical University of Bialystok, Kilińskiego 1 Str., 15-089 Białystok, Poland; kkramk@wp.pl
* Correspondence: p.kowalczyk@ifzz.pl (P.K.); dominik.koszelewski@icho.edu.pl (D.K.)
† These authos have equally contributed equally to the paper.

Citation: Samsonowicz-Górski, J.; Kowalczyk, P.; Koszelewski, D.; Brodzka, A.; Szymczak, M.; Kramkowski, K.; Ostaszewski, R. The Synthesis and Evaluation of Amidoximes as Cytotoxic Agents on Model Bacterial *E. coli* Strains. *Materials* **2021**, *14*, 7577. https://doi.org/10.3390/ma14247577

Academic Editor: Claudio Canale

Received: 18 November 2021
Accepted: 07 December 2021
Published: 9 December 2021

Publisher's Note: MDPI stays neutral with regard to jurisdictional claims in published maps and institutional affiliations.

Copyright: © 2021 by the authors. Licensee MDPI, Basel, Switzerland. This article is an open access article distributed under the terms and conditions of the Creative Commons Attribution (CC BY) license (http://creativecommons.org/licenses/by/4.0/).

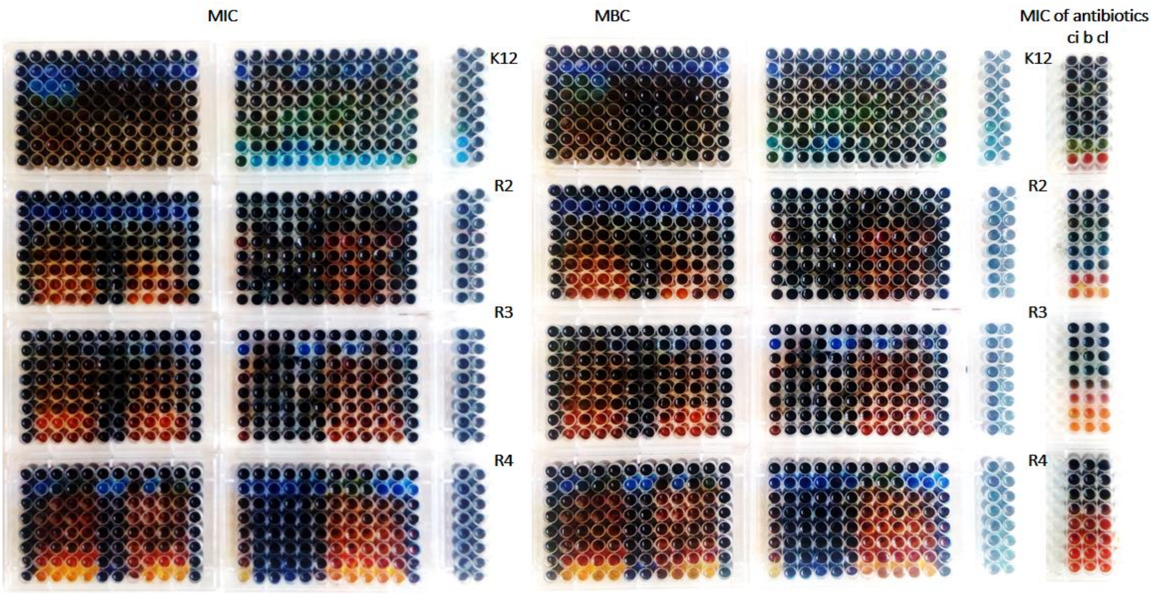

Figure S1. Examples of MIC and MBC on microplates with different concentration of studied compounds (μg/mL^{-1}). Resazurin was added as an indicator of microbial growth with K12, R2, R3, and R4 strains with tested 26 compounds, as described in Table 2. Additionally, examples of MIC with different strains K12, R2, R3, and R4 of studied antibiotics with ciprofloxacin (ci), bleomycin (b), and cloxacillin (cl) in (μg/mL^{-1}).

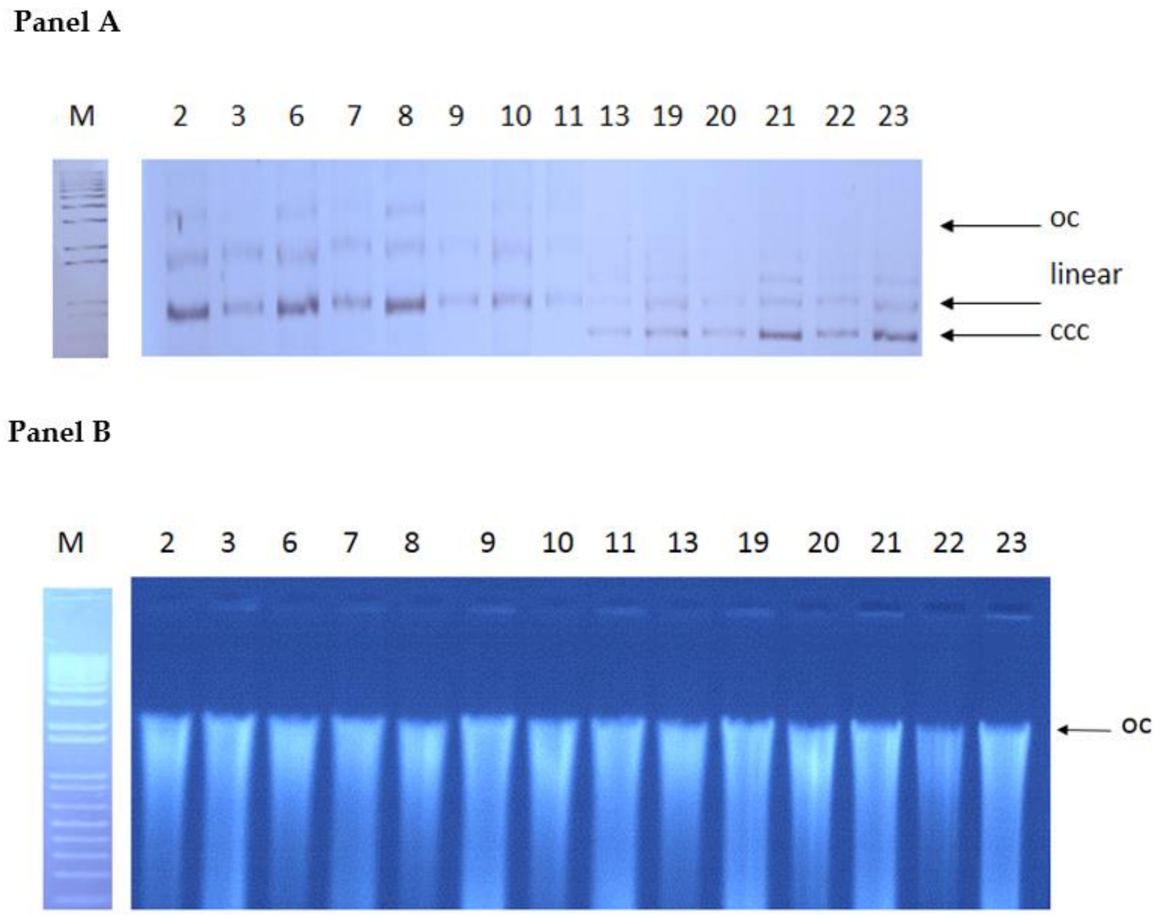

Figure S2. An example of an agarose gel electrophoresis separation of isolated plasmids DNA on R4 strains modified with selected coumarin derivatives (Panel A) from 8 selected compounds, as shown in Figure 3, and digested with repair Fpg protein (Panel B). M = marker.

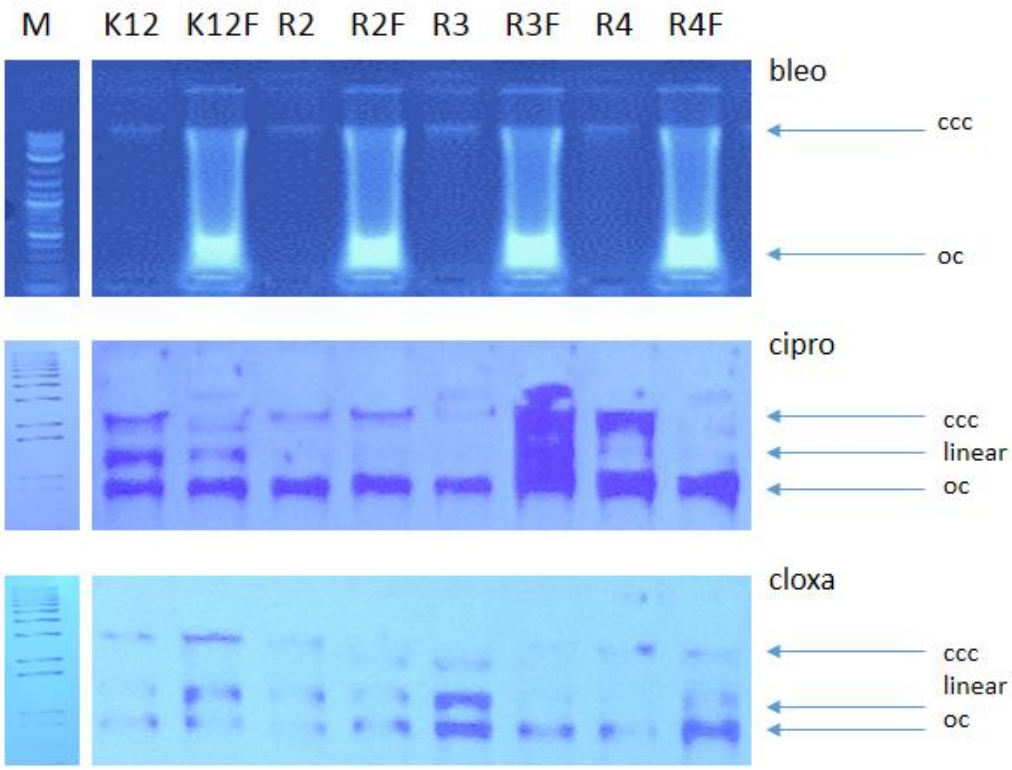

Figure S3. Example of an agarose gel electrophoresis separation of isolated plasmids DNA from K12 and R4 strains modified with antibiotics: bleomycin, ciprofloxacin, and cloxacillin digested (or not) with repair enzymes Fpg. M = marker.

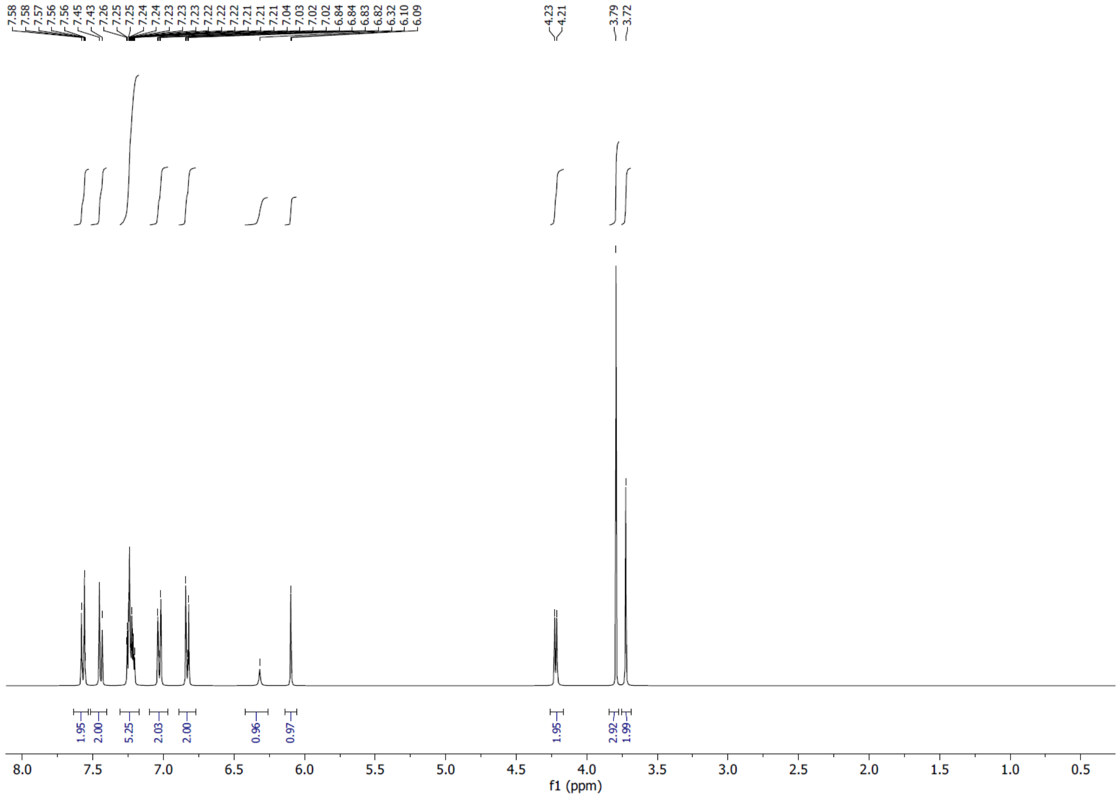

Figure S4. ^{1}H NMR (400 MHz, $CDCl_3$) of 2-(4-methoxybenzylamino)-2-oxo-1-(4-cyanophenyl)ethyl phenylacetate.

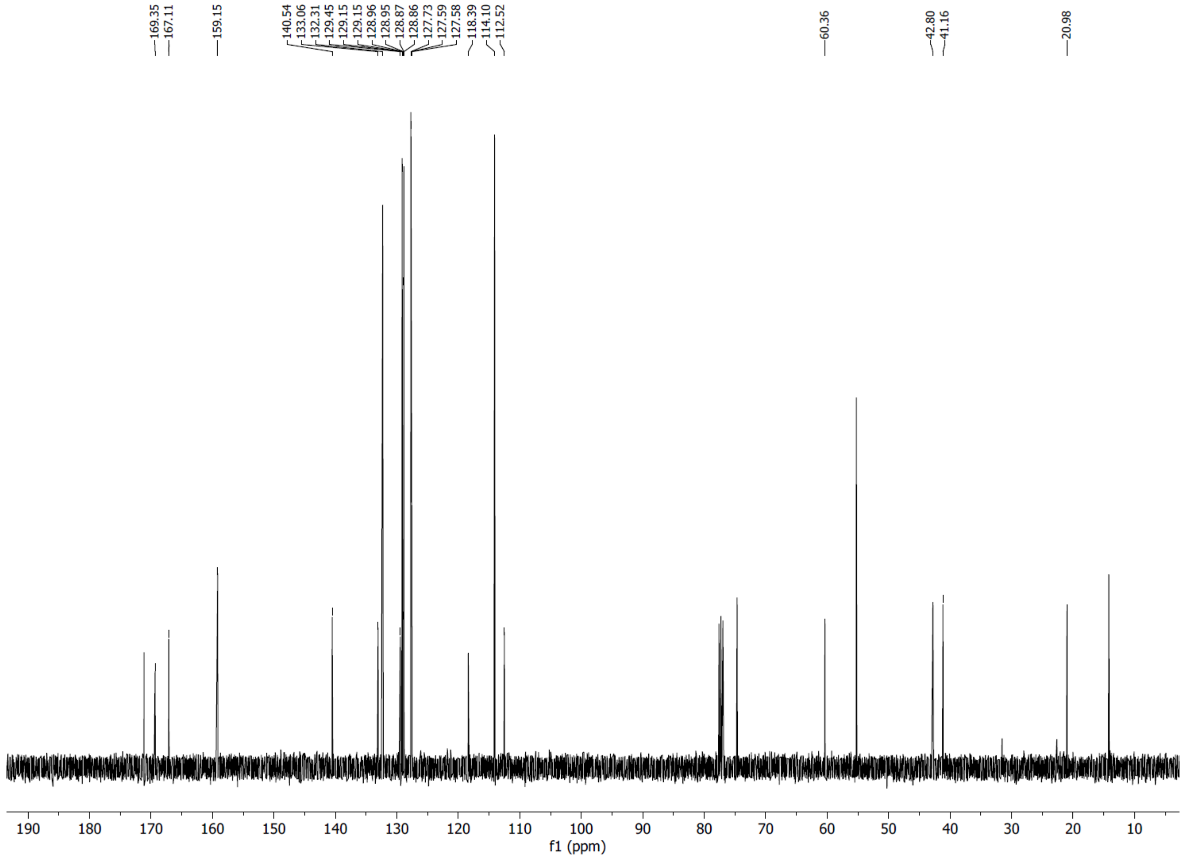

Figure S5. ^{13}C NMR (100 MHz, $CDCl_3$) of 2-(4-methoxybenzylamino)-2-oxo-1-(4-cyanophenyl)ethyl phenylacetate.

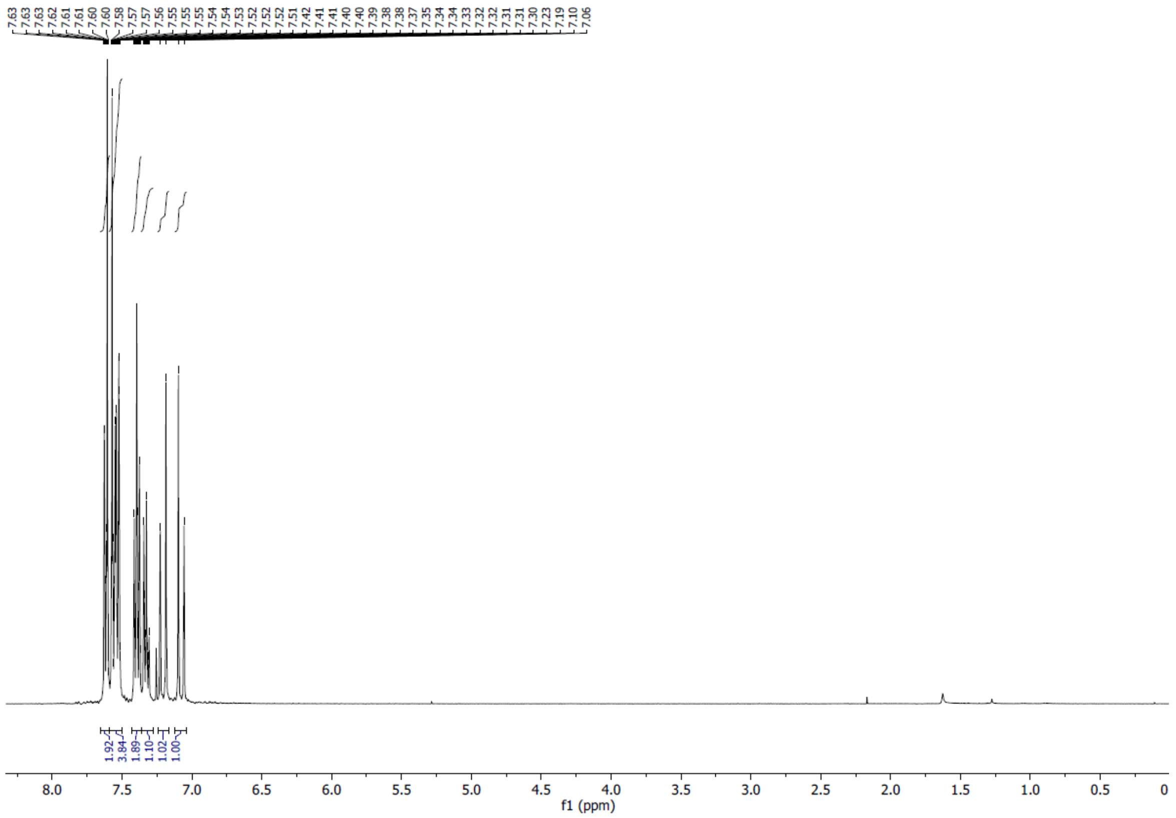

Figure S6. ^{1}H NMR (400 MHz, $CDCl_3$) of 4-cyanostilbene.

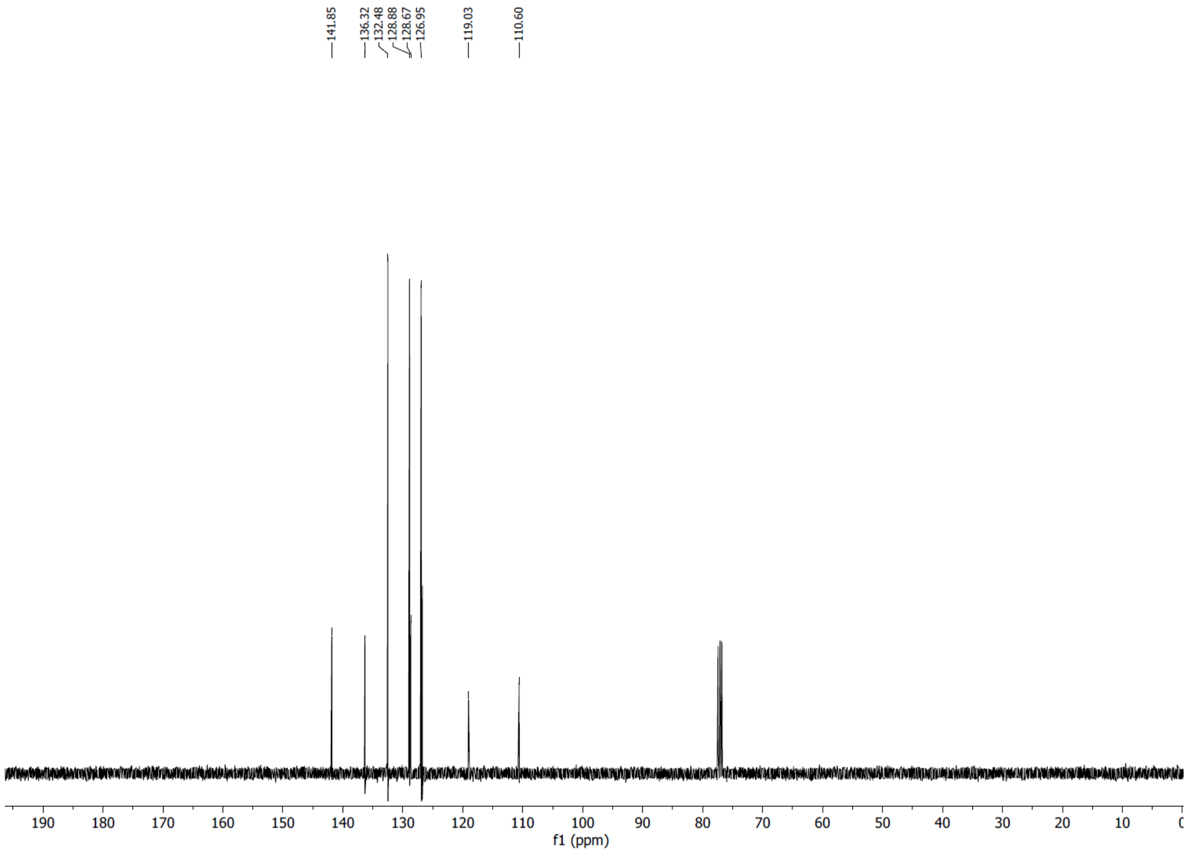

Figure S7. ^{13}C NMR (100 MHz, $CDCl_3$) of 4-cyanostilbene.

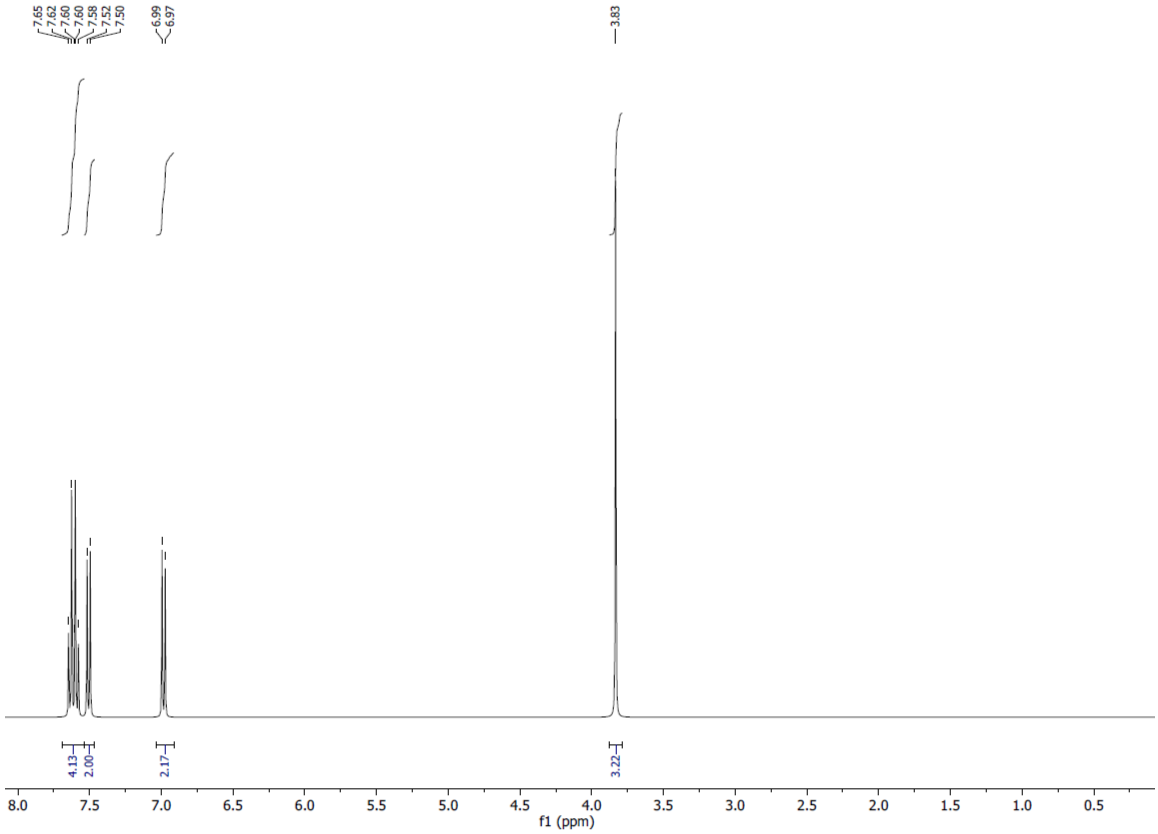

Figure S8. ^{1}H NMR (400 MHz, $CDCl_3$) of 4'-methoxybiphenyl-4-carbonitrile.

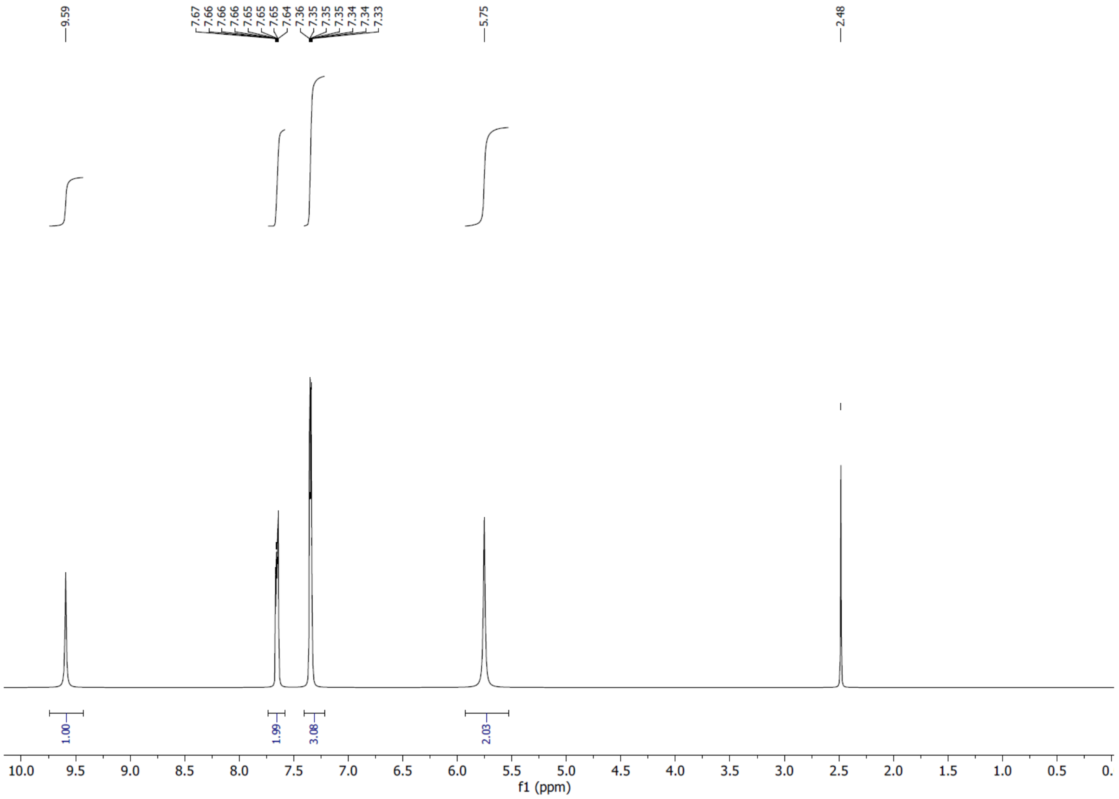

Figure S9. ^{1}H NMR (400 MHz, DMSO-d_6) spectra of *N*'-hydroxybenzimidamide (**1**).

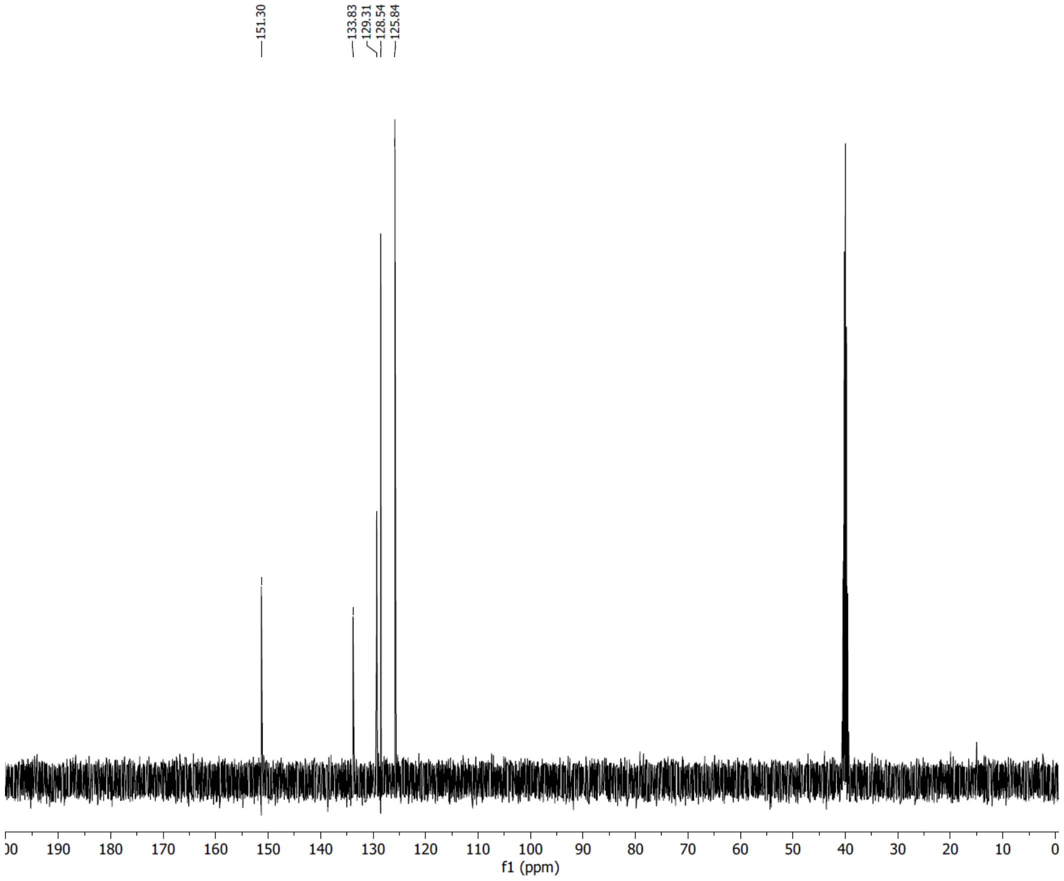

Figure S10. ^{13}C NMR (100 MHz, DMSO-d_6) spectra of *N*'-hydroxybenzimidamide (**1**).

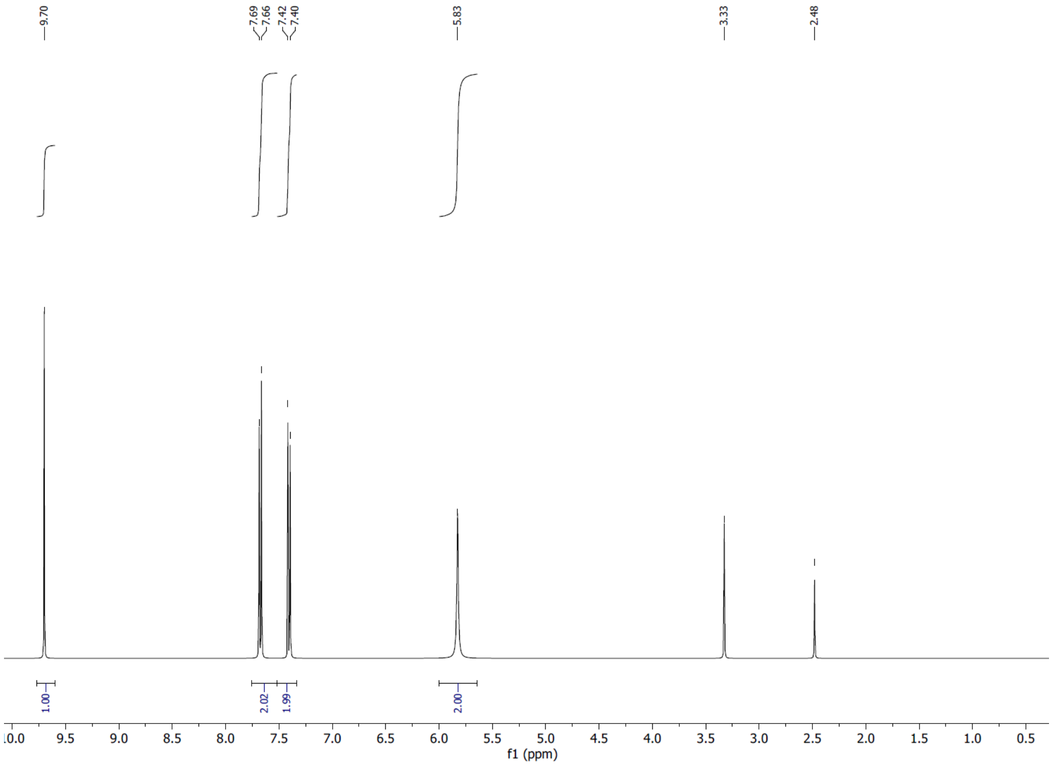

Figure S11. ^{1}H NMR (400 MHz, DMSO-d_6) spectra of 4-chlorobenzamidoxime (**2**).

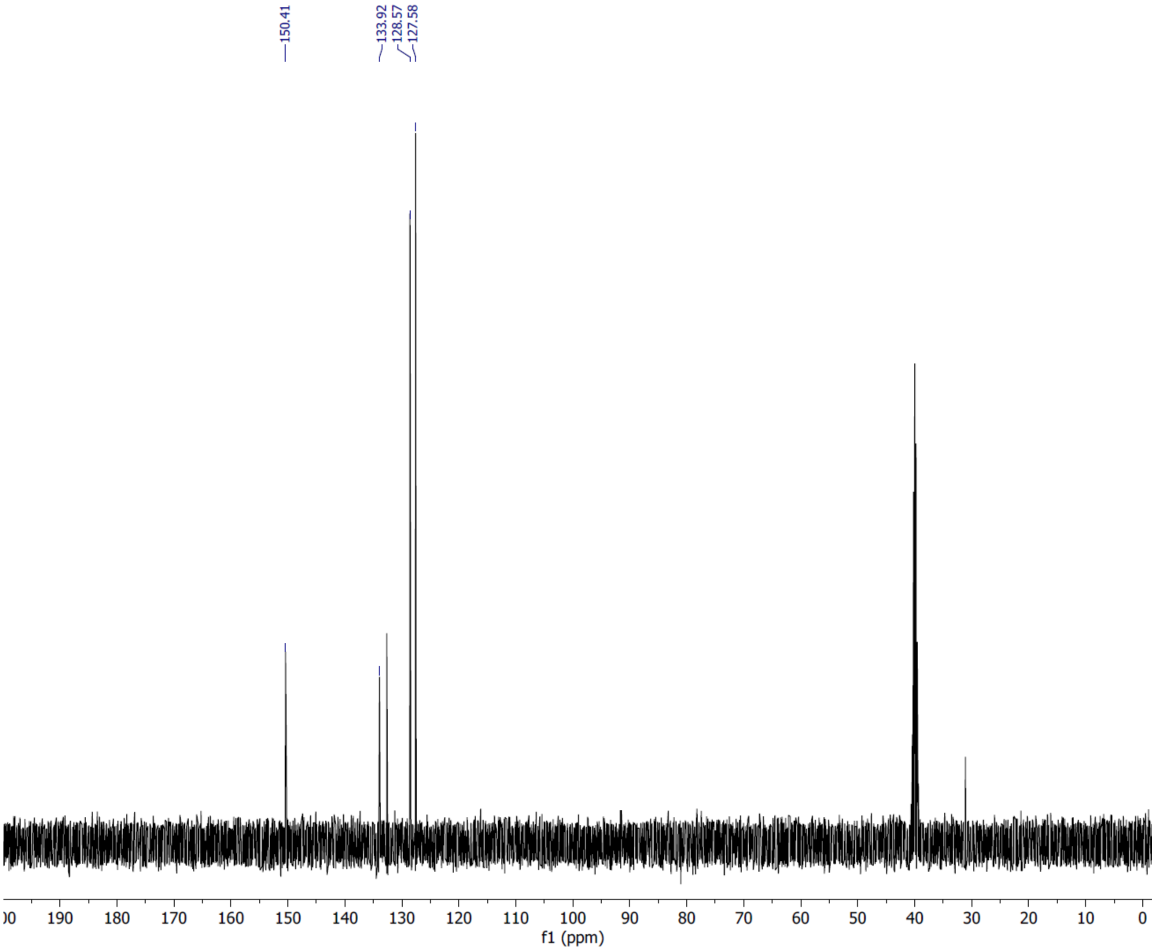

Figure S12. ^{13}C NMR (100 MHz, DMSO-d_6) spectra of 4-chlorobenzamidoxime (**2**).

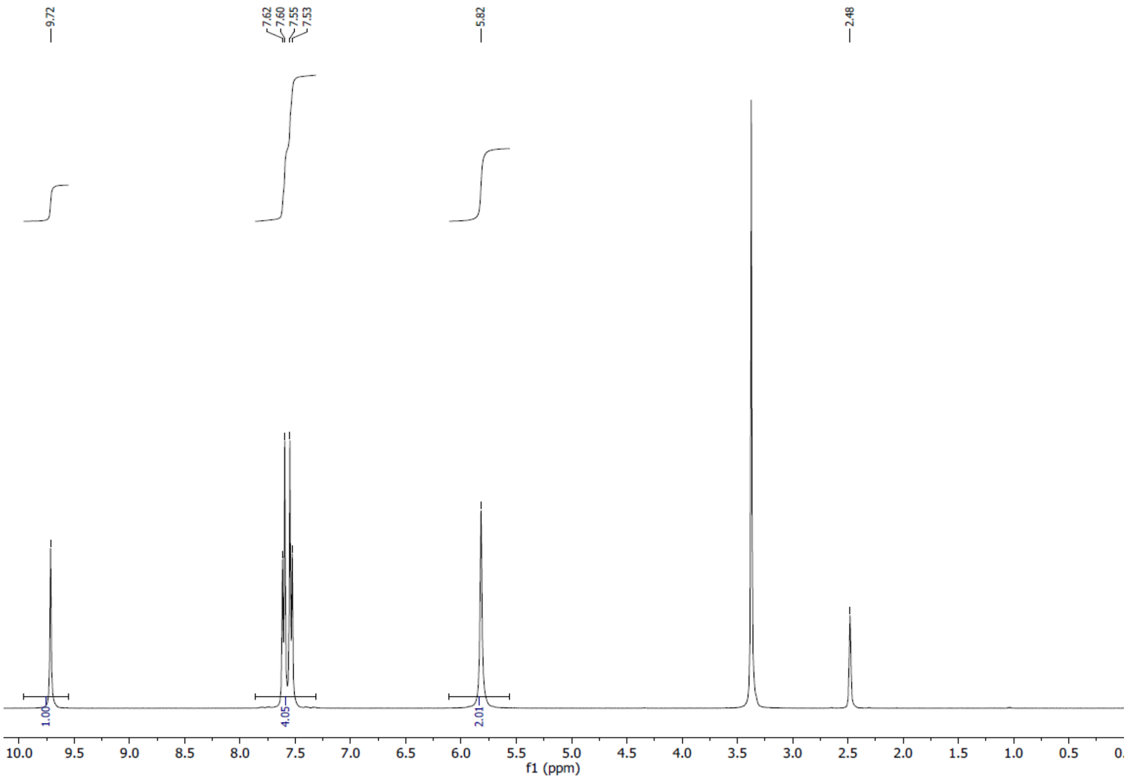

Figure S13. ^{1}H NMR (400 MHz, DMSO-d_6) spectra of *p*-bromobenzamidoxime (**3**).

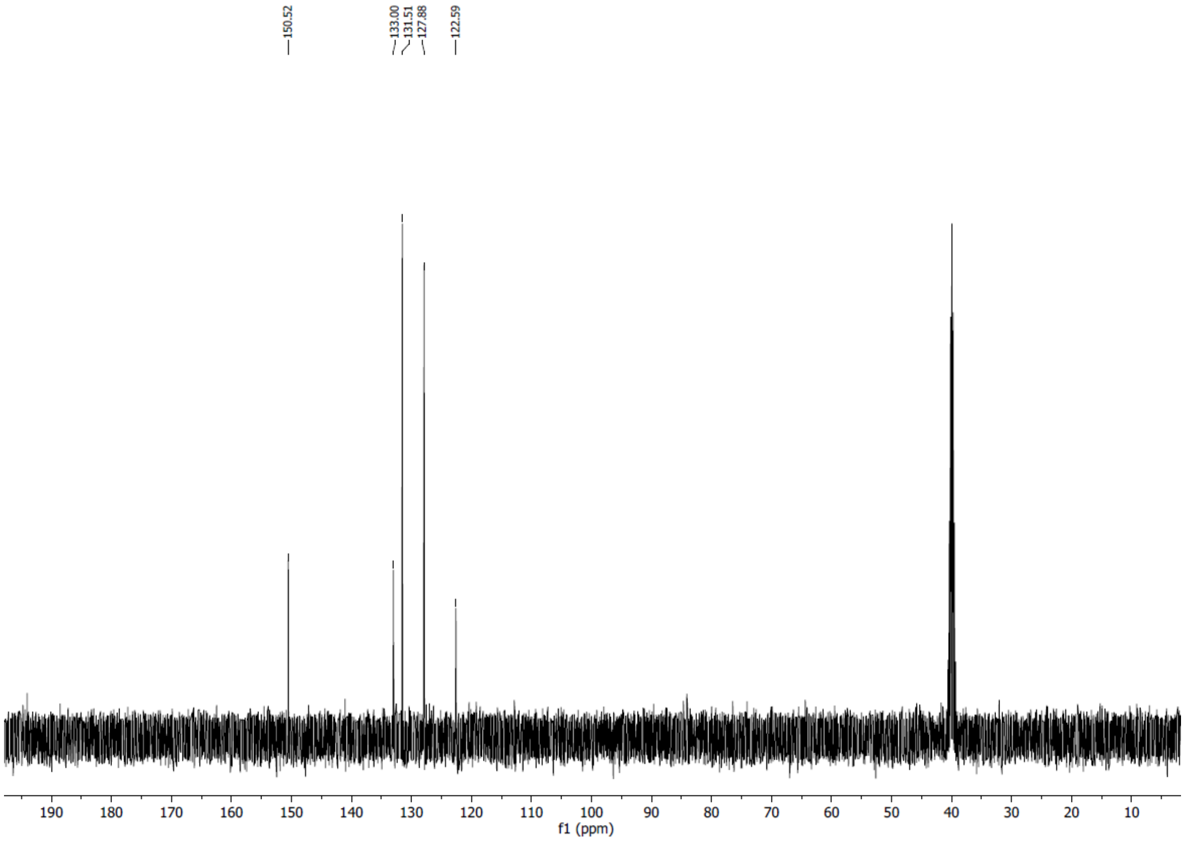

Figure S14. ^{13}C NMR (100 MHz, DMSO-d_6) spectra of *p*-bromobenzamidoxime (**3**).

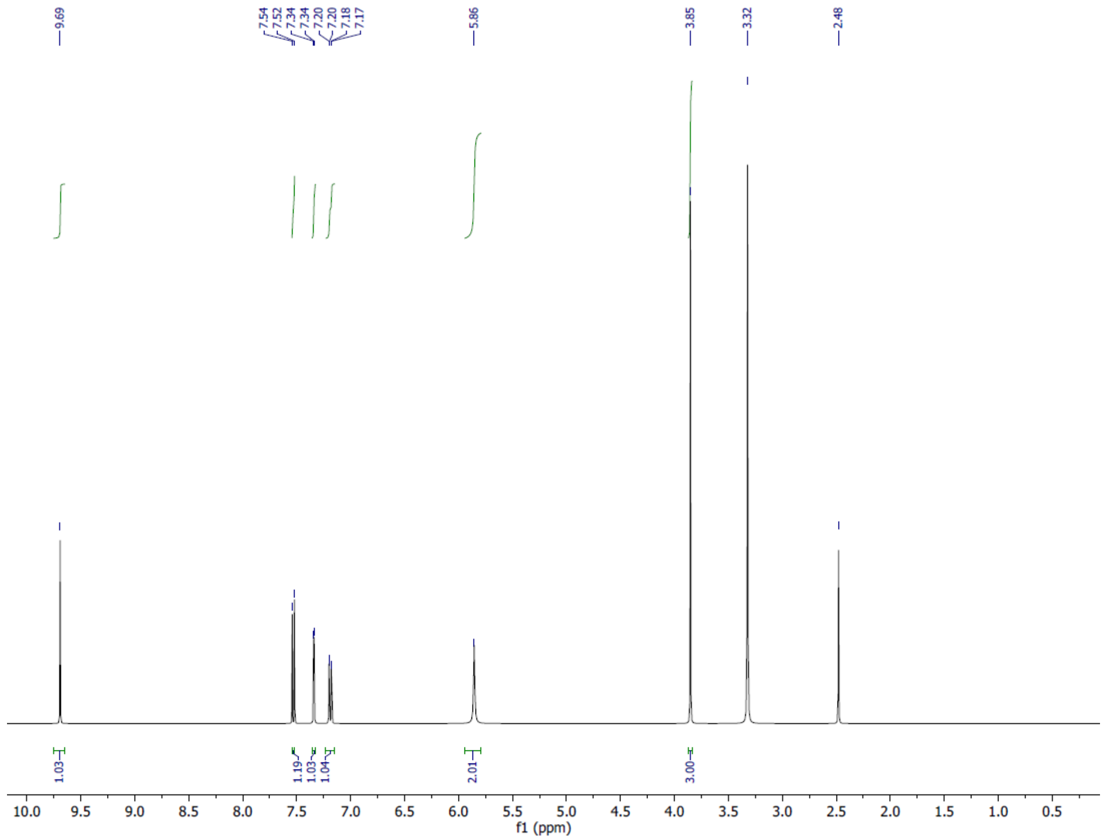

Figure S15. ^{1}H NMR (400 MHz, DMSO-d$_6$) spectra of 4-bromo-*N*′-hydroxy-3-methoxybenzimidamide (**4**).

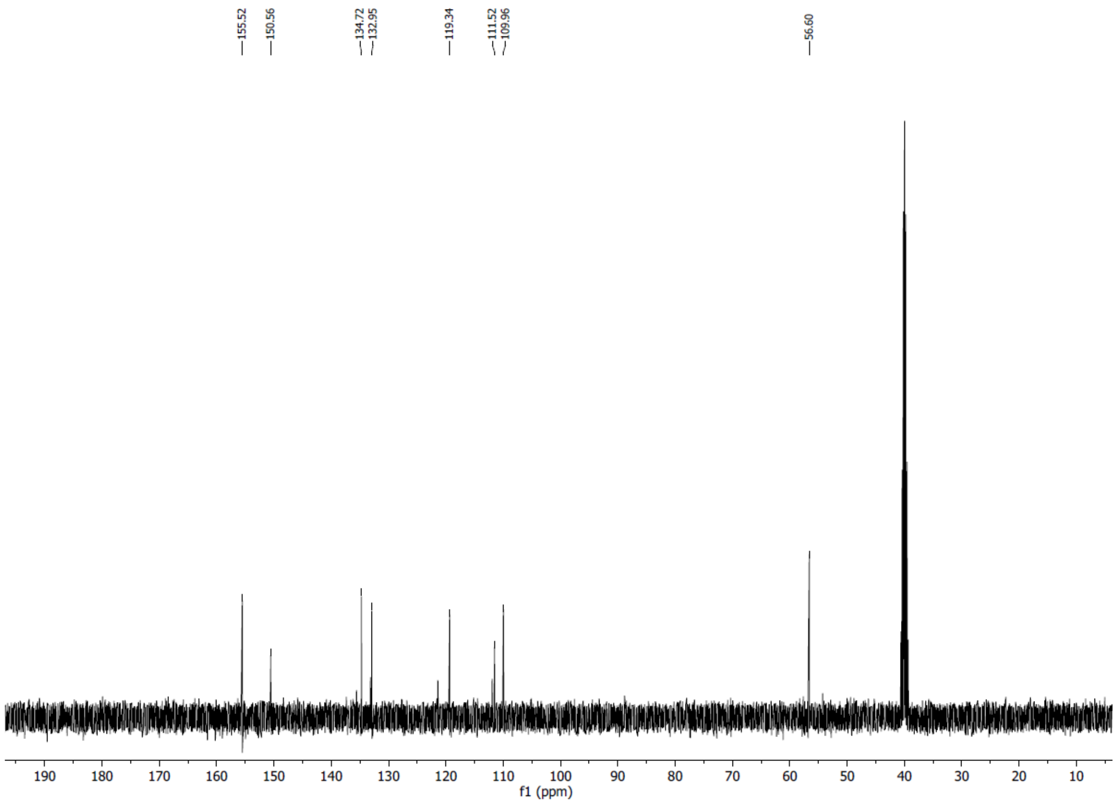

Figure S16. ^{13}C NMR (100 MHz, DMSO-d$_6$) spectra of 4-bromo-*N*′-hydroxy-3-methoxybenzimidamide (**4**)

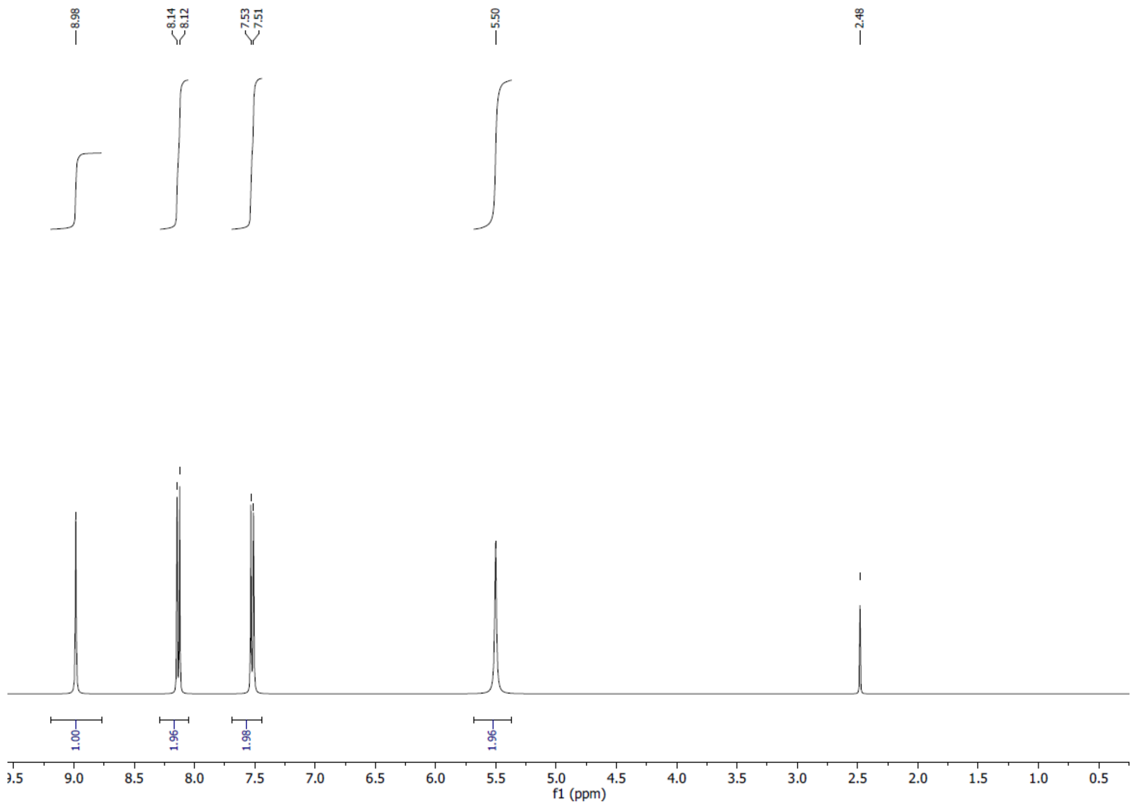

Figure S17. ^{1}H NMR (400 MHz, DMSO-d_6) spectra of *N*'-hydroxy-4-nitrobenzimidamide (**5**).

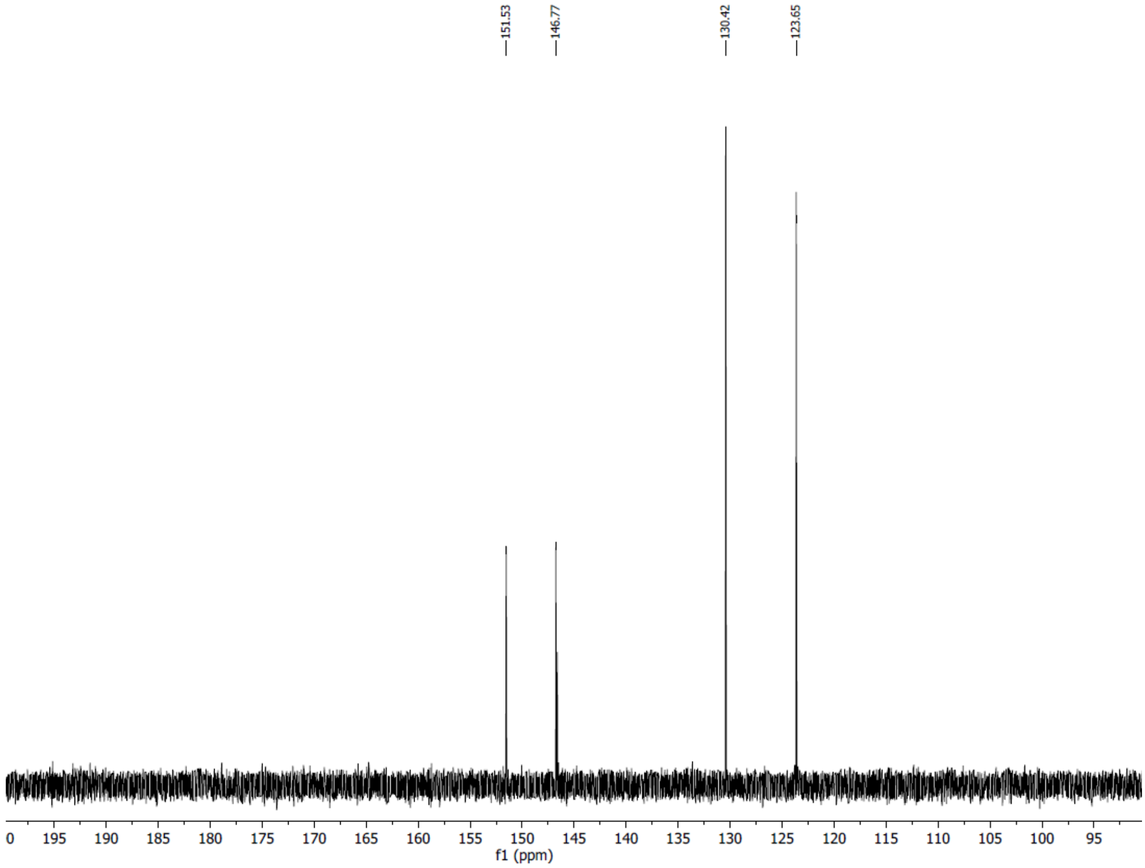

Figure S18. ^{13}C NMR (100 MHz, DMSO-d_6) spectra of *N*'-hydroxy-4-nitrobenzimidamide (**5**).

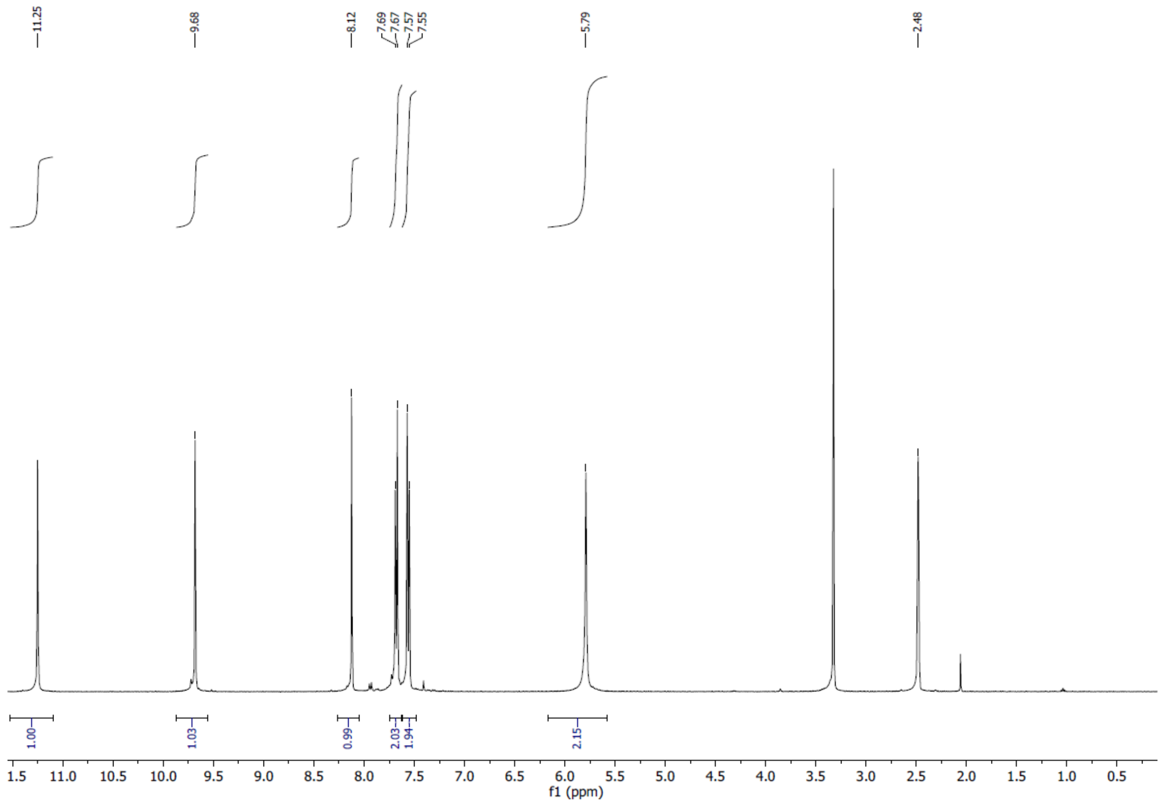

Figure S19. ^{1}H NMR (400 MHz, DMSO-d_6) spectra of *N*'-Hydroxy-4-((hydroxyimino)methyl)benzimidamide (**6**).

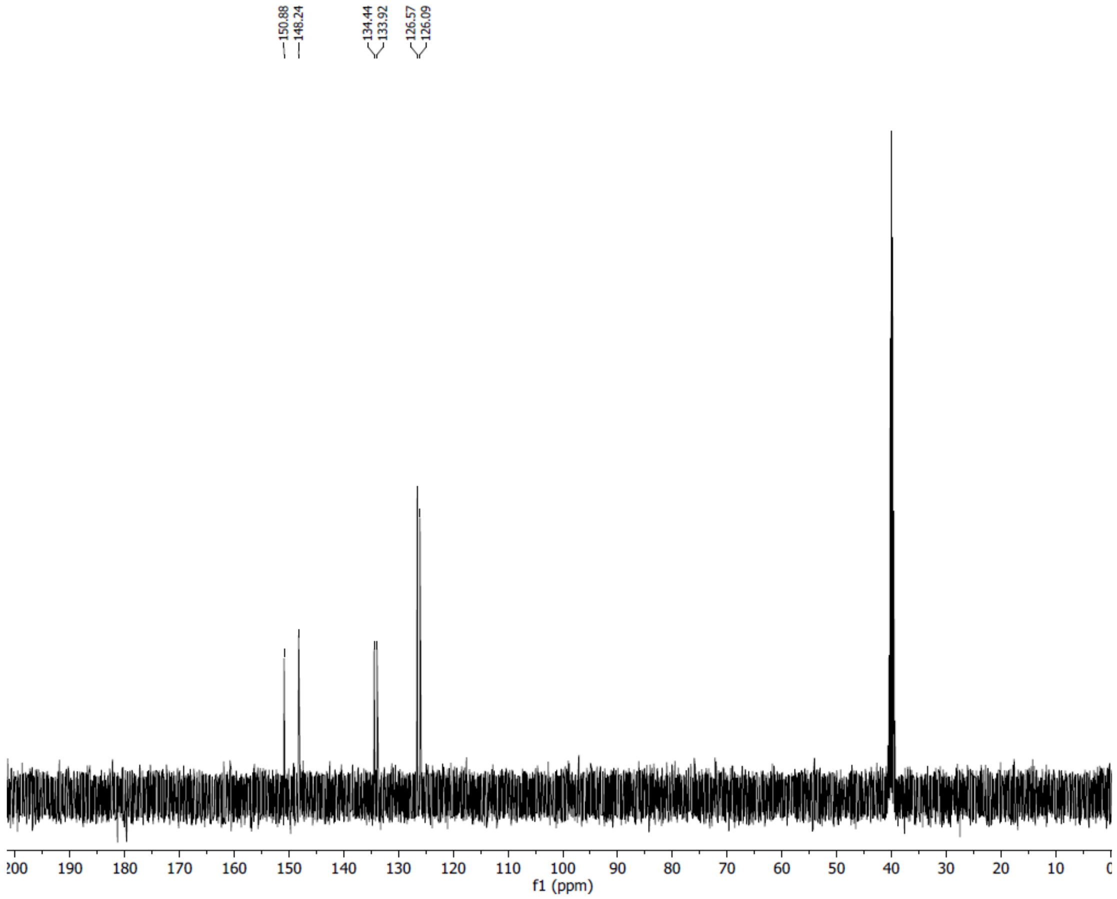

Figure S20. ^{13}C NMR (100 MHz, DMSO-d_6) spectra of *N*'-Hydroxy-4-((hydroxyimino)methyl)benzimidamide (**6**)

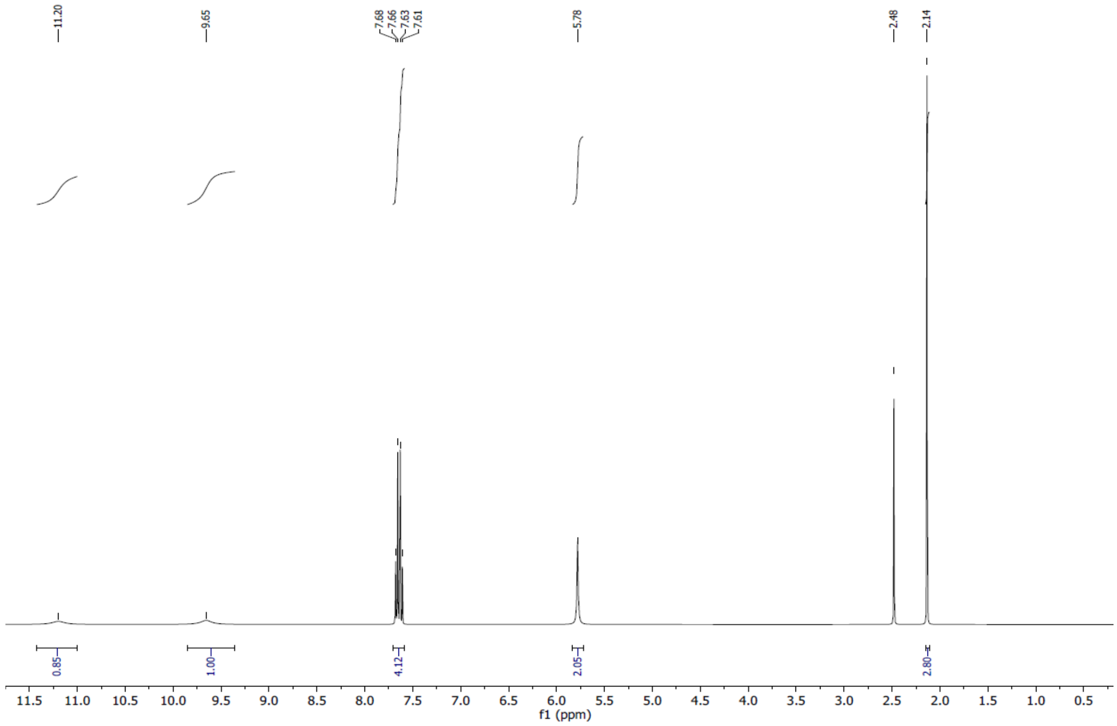

Figure S21. ^{1}H NMR (400 MHz, DMSO-d_6) spectra of *N*'-Hydroxy-4-[*N*-hydroxyethanimidoyl]benzene-1-carboximidamide (**7**)

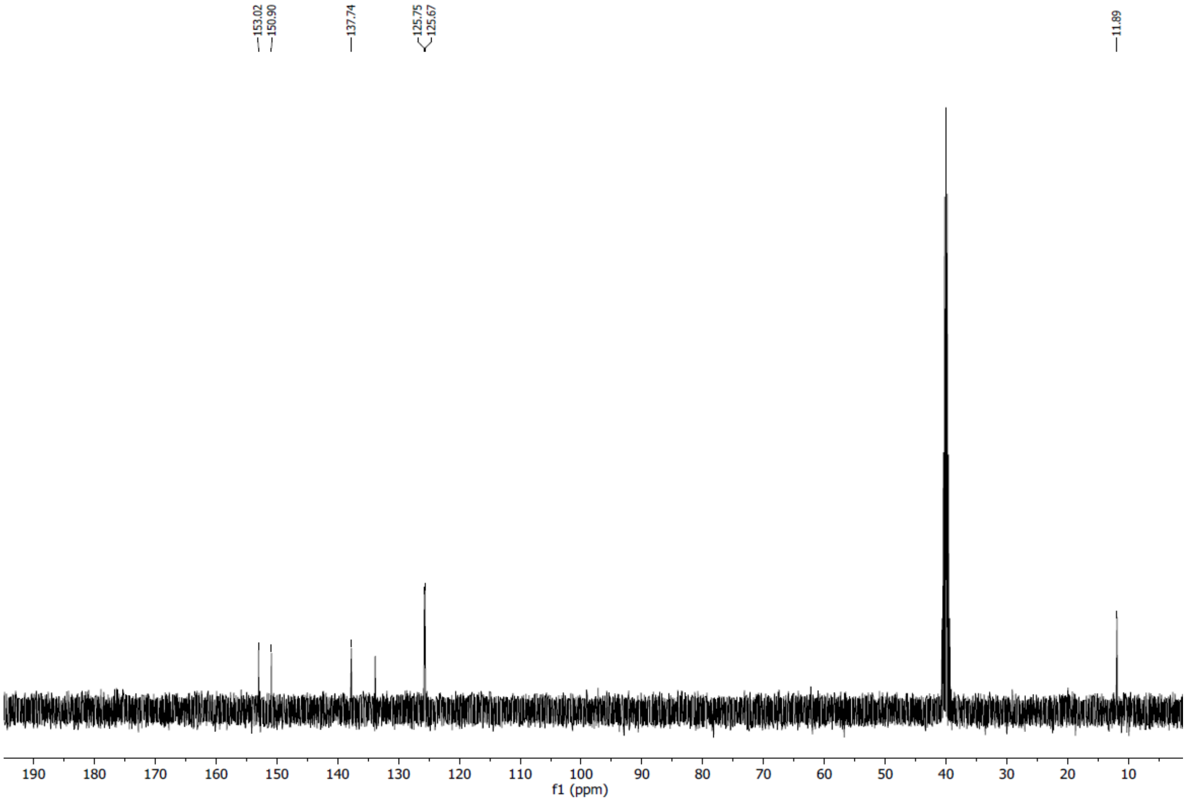

Figure S22. ^{13}C NMR (100 MHz, DMSO-d_6) spectra of *N*'-Hydroxy-4-[*N*-hydroxyethanimidoyl]benzene-1-carboximidamide (**7**)

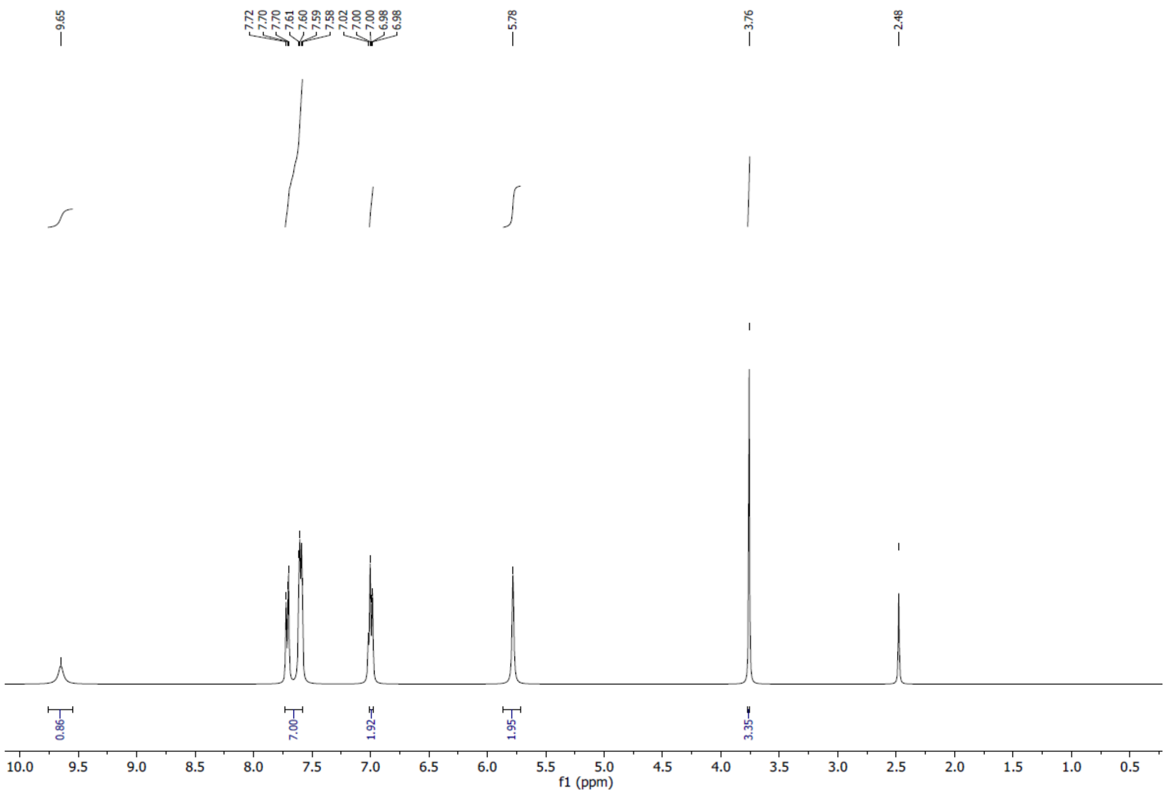

Figure S23. ^{1}H NMR (400 MHz, DMSO-d$_6$) spectra of 4-(4-Methoxyphenyl)phenyl]-nitrosomethanamine (**8**)

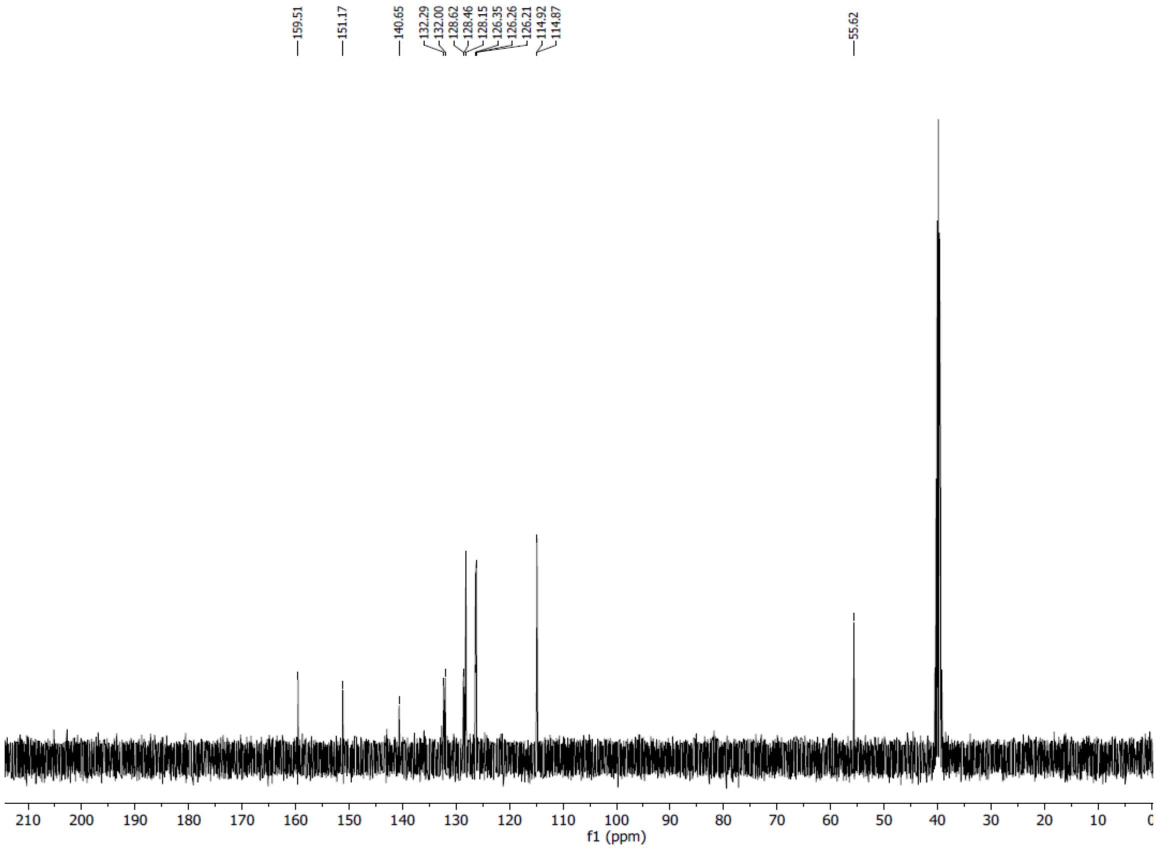

Figure S24. ^{1}H NMR (400 MHz, DMSO-d$_6$) spectra of 4-(4-Methoxyphenyl)phenyl]-nitrosomethanamine (**8**)

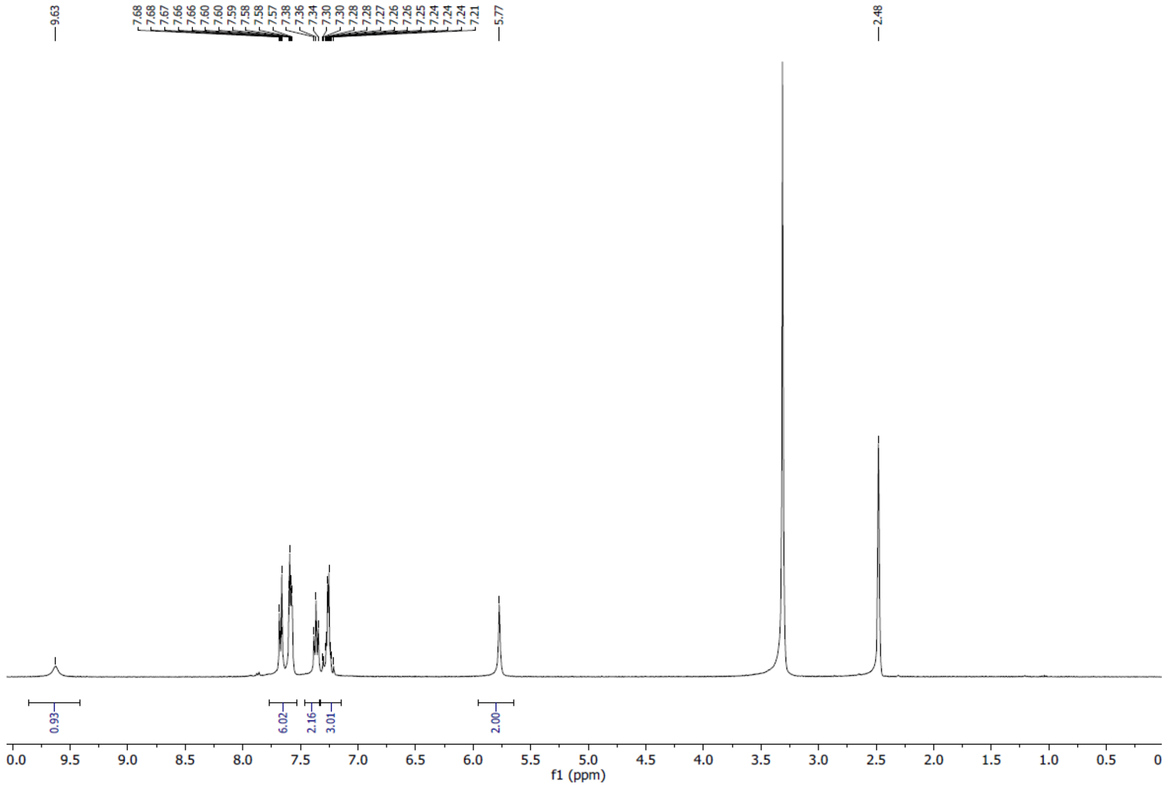

Figure S25. ^1H NMR (400 MHz, DMSO-d_6) spectra of *N′*-hydroxy-4-[(*E*)-2-phenylethenyl]benzene-1-carboximidamide (**9**)

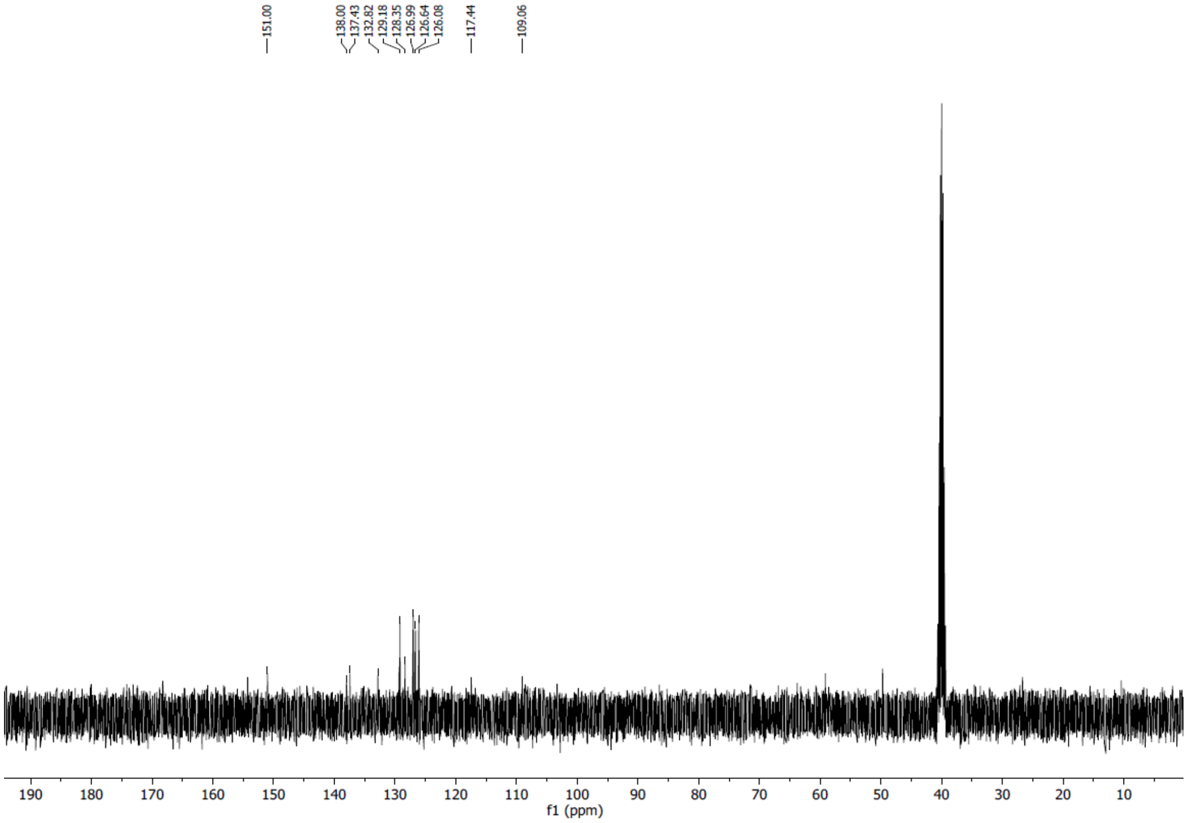

Figure S26. ^{13}C NMR (100 MHz, DMSO-d_6) spectra of *N′*-Hydroxy-4-[(*E*)-2-phenylethenyl]benzene-1-carboximidamide (**9**)

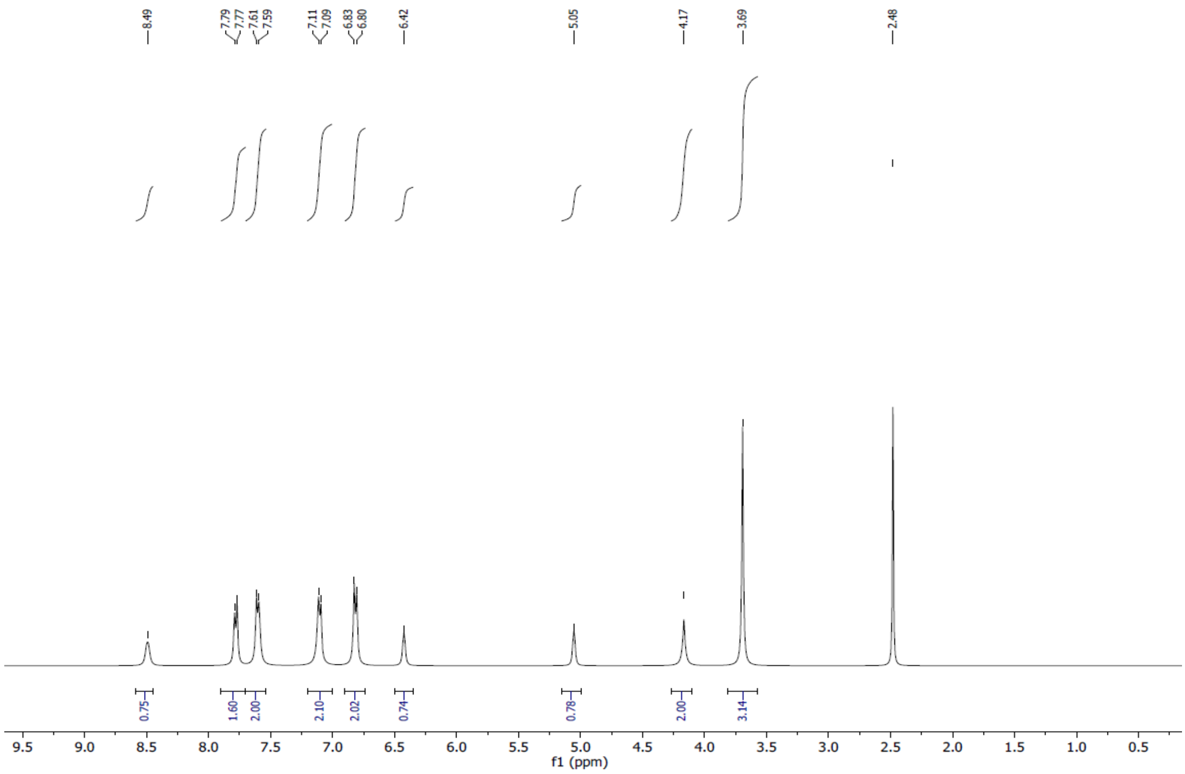

Figure S27. ^{1}H NMR (400 MHz, DMSO-d6) spectra of 2-{4-[1-amino-2-hydroxyethenyl]phenyl}-2-hydroxy-*N*-[(4-methoxyphenyl)methyl]acetamide (**10**)

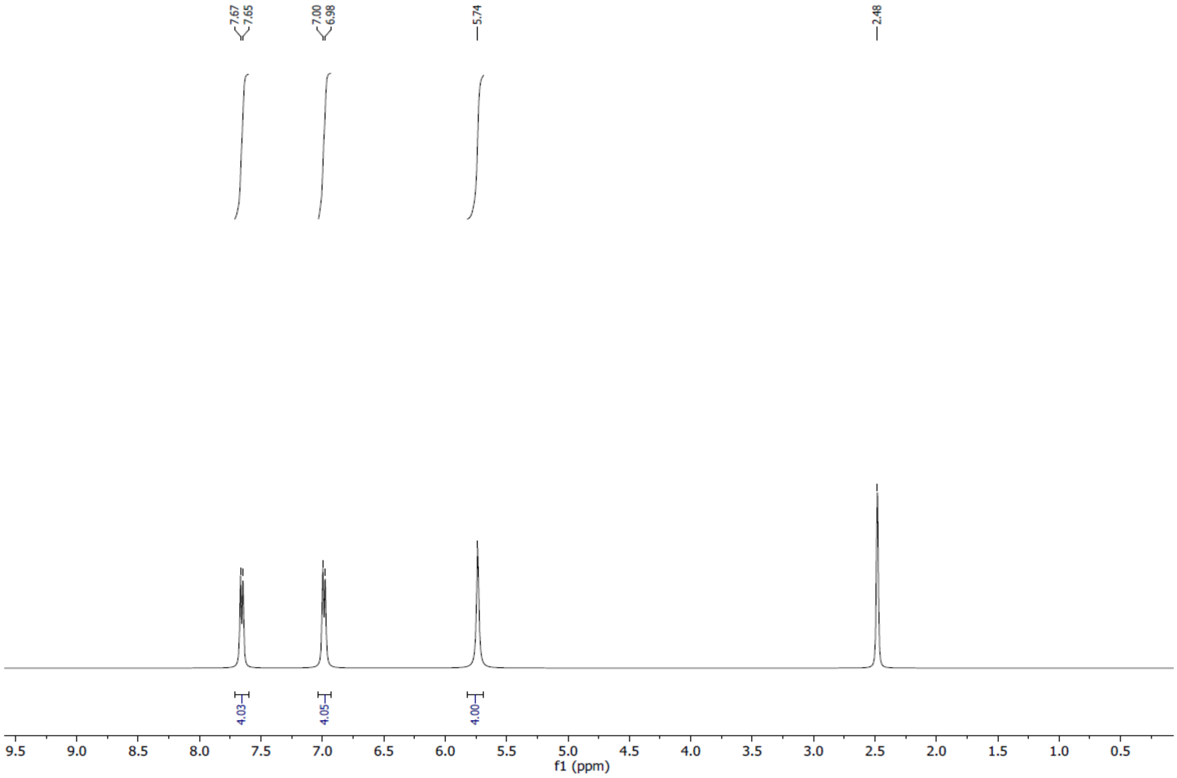

Figure S28. ^{1}H NMR (400 MHz, DMSO-d6) spectra of 4,4'-Oxy-bis-benzamide oxime (**11**)

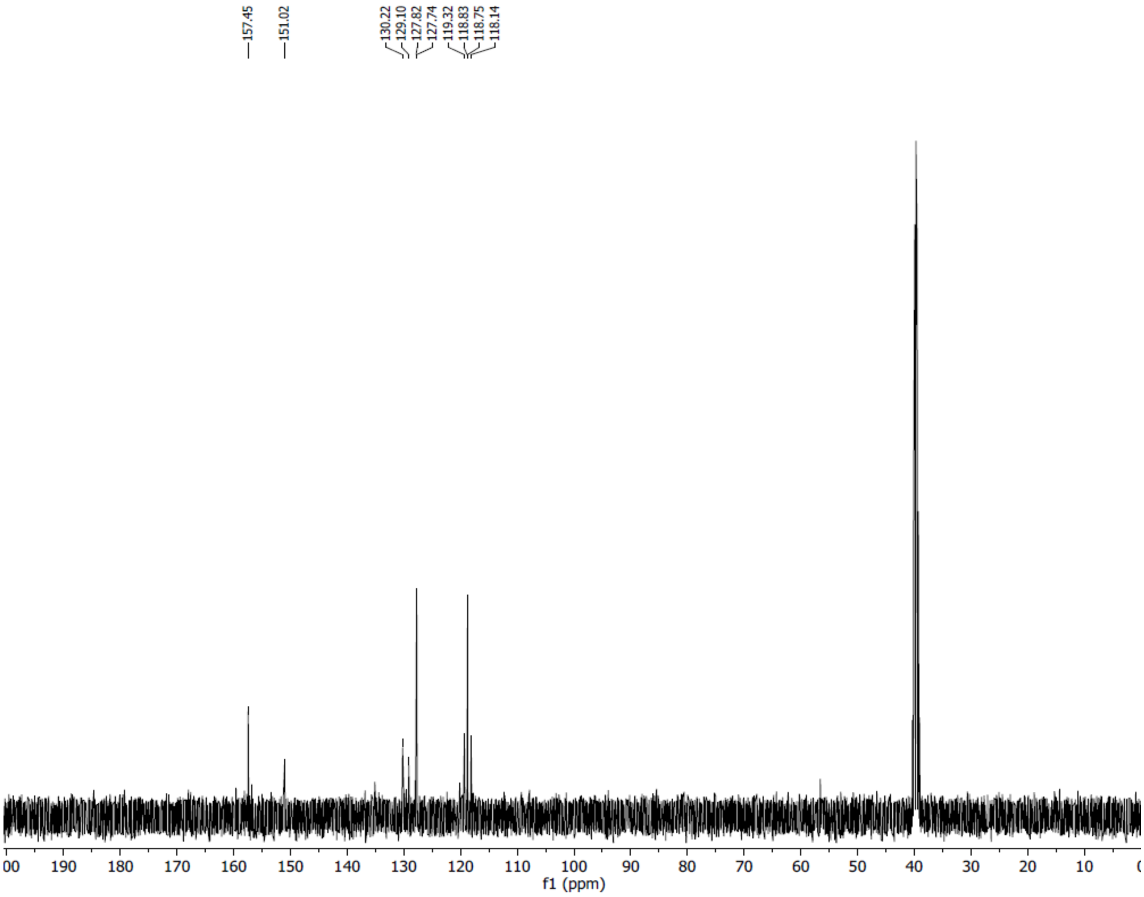

Figure S29. ^{13}C NMR (100 MHz, DMSO-d_6) spectra of 4,4'-oxy-bis-benzamide oxime (**11**)

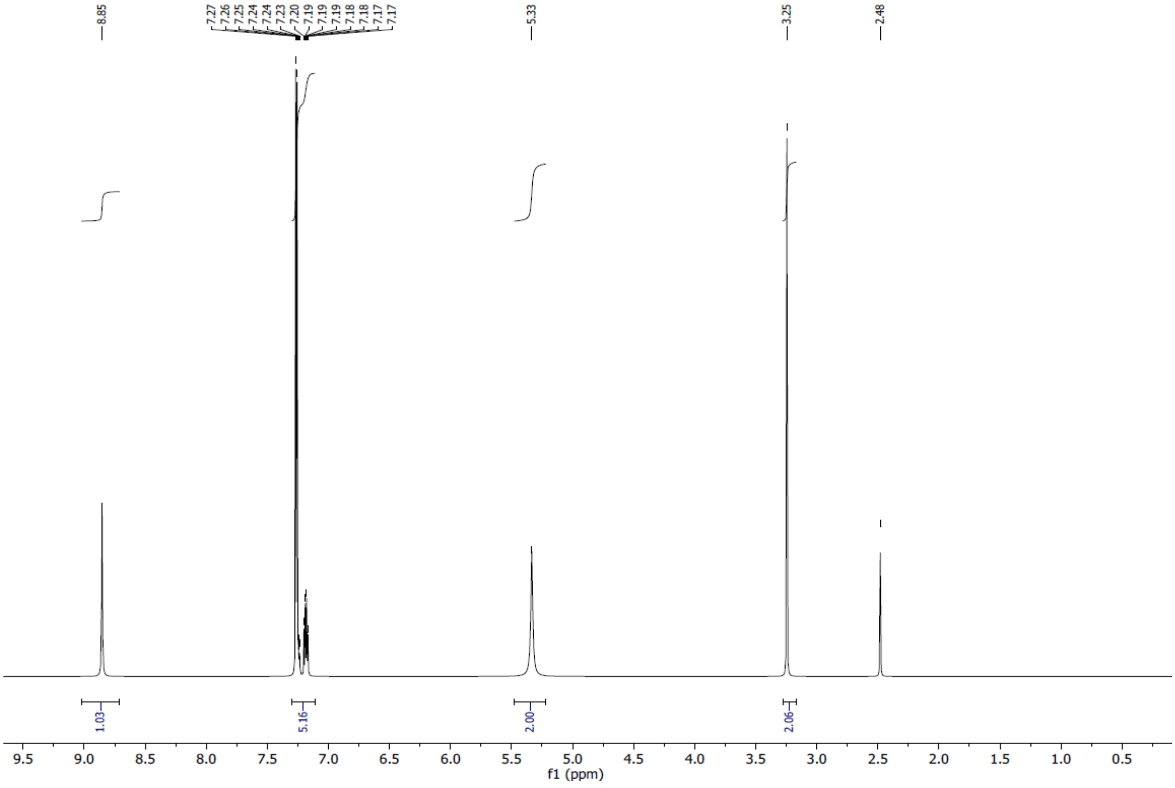

Figure S30. ^{1}H NMR (400 MHz, DMSO-d_6) spectra of benzylamidoxime (**12**)

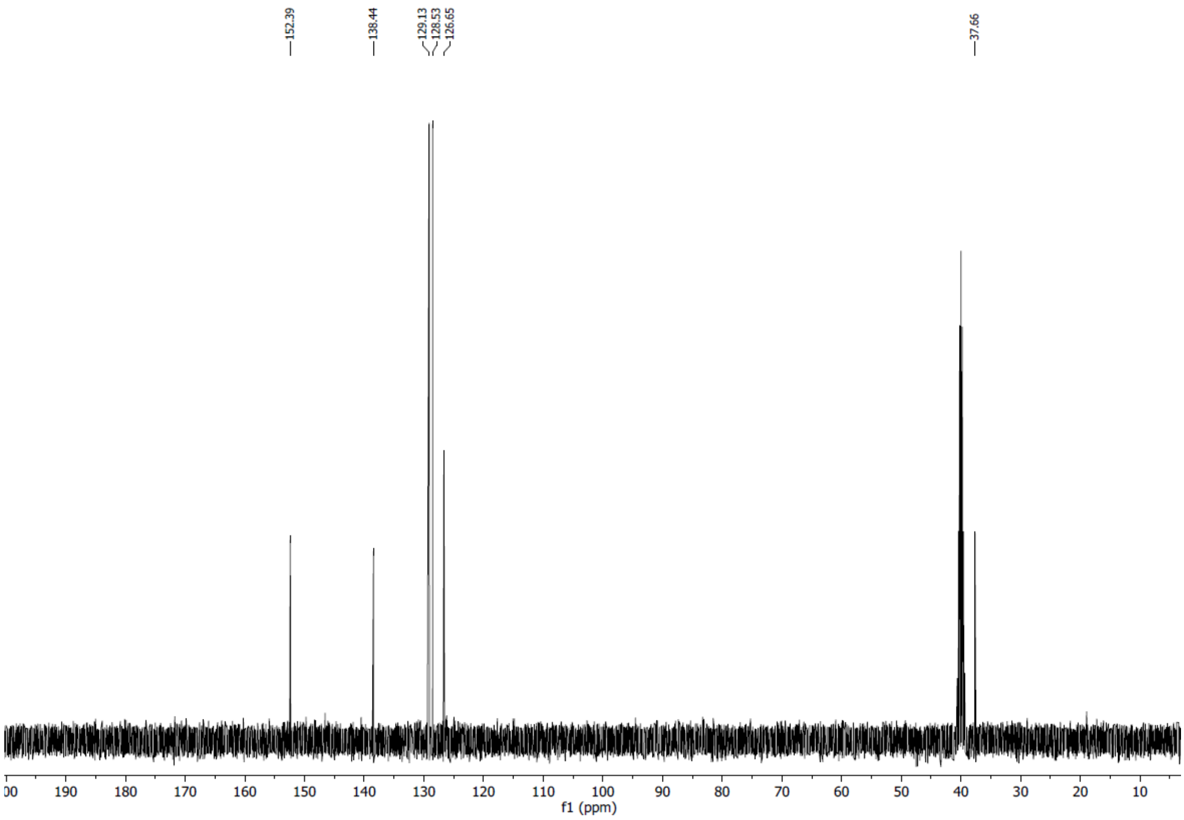

Figure S31. ^{13}C NMR (100 MHz, DMSO-d_6) spectra of benzylamidoxime (**12**)

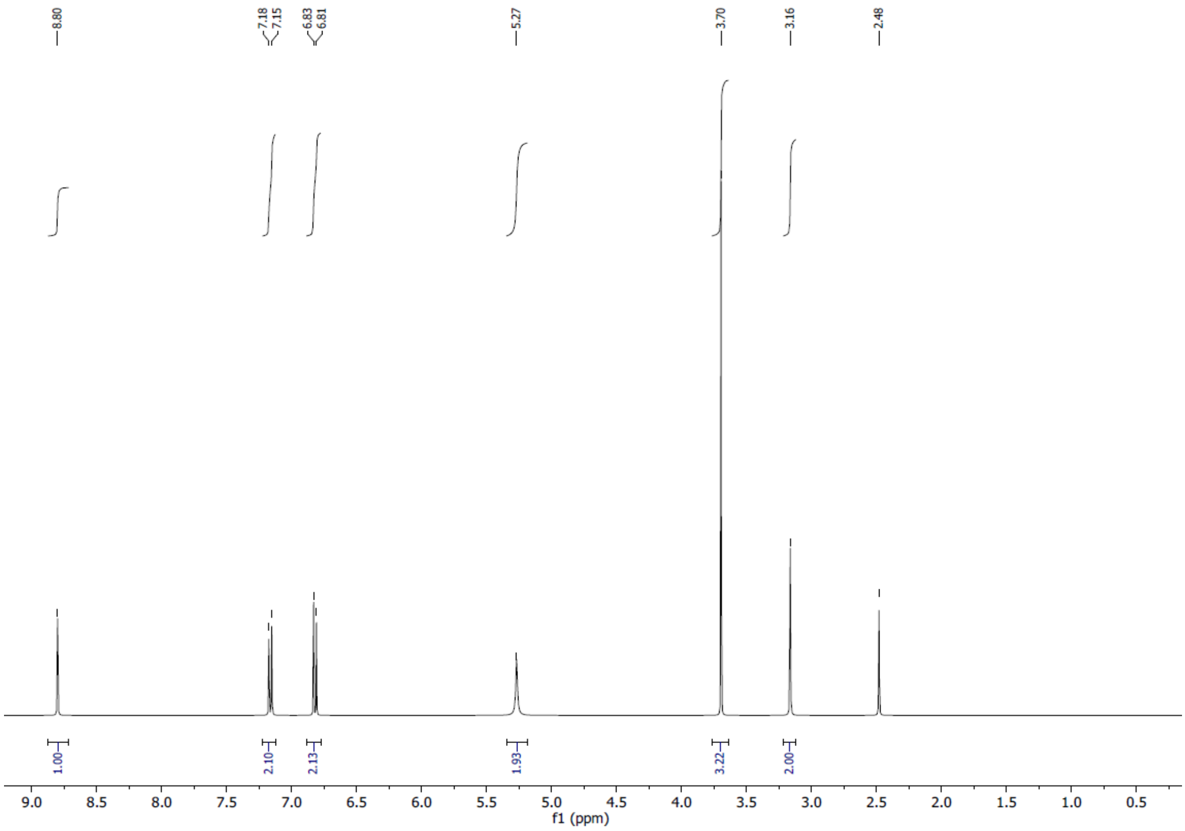

Figure S32. ^{1}H NMR (400 MHz, DMSO-d_6) spectra of *N′*-hydroxy-2-(4-methoxyphenyl)ethanimidamide (**13**)

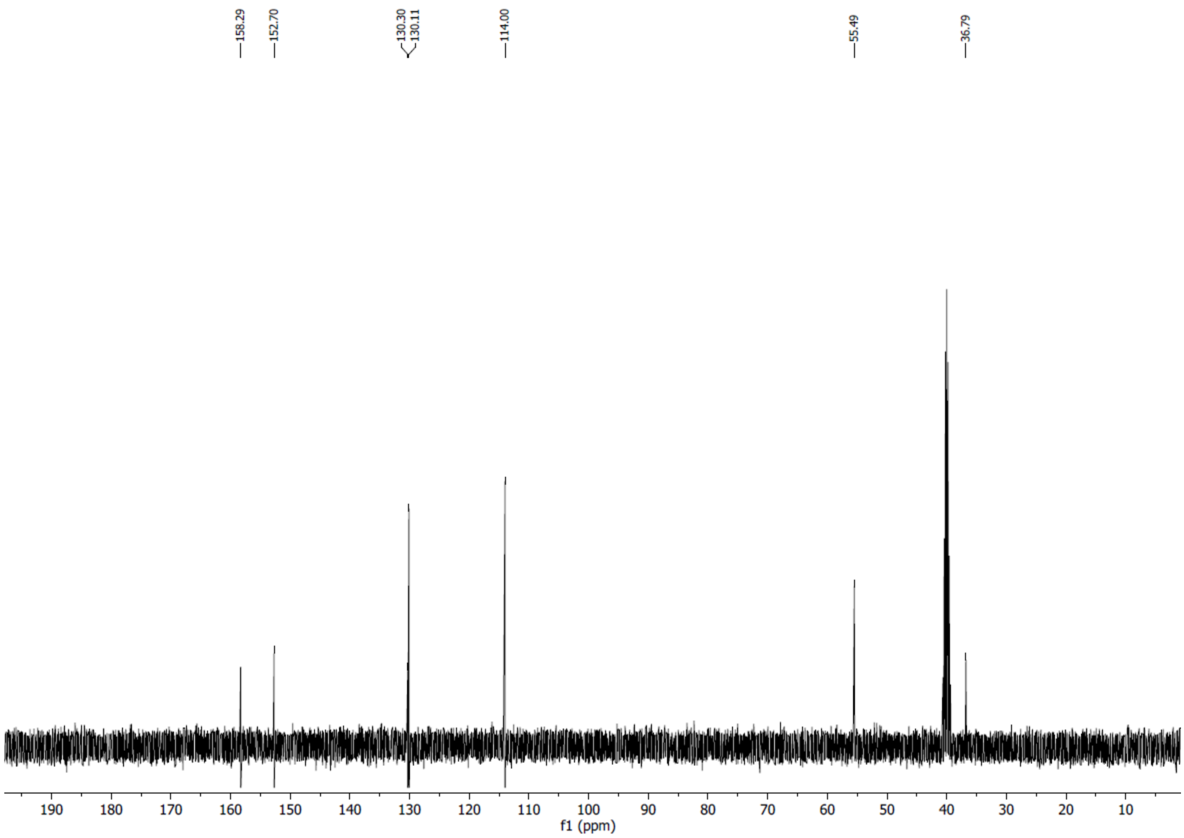

Figure S33. ^{13}C NMR (100 MHz, DMSO-d_6) spectra of *N′*-hydroxy-2-(4-methoxyphenyl)ethanimidamide (**13**)

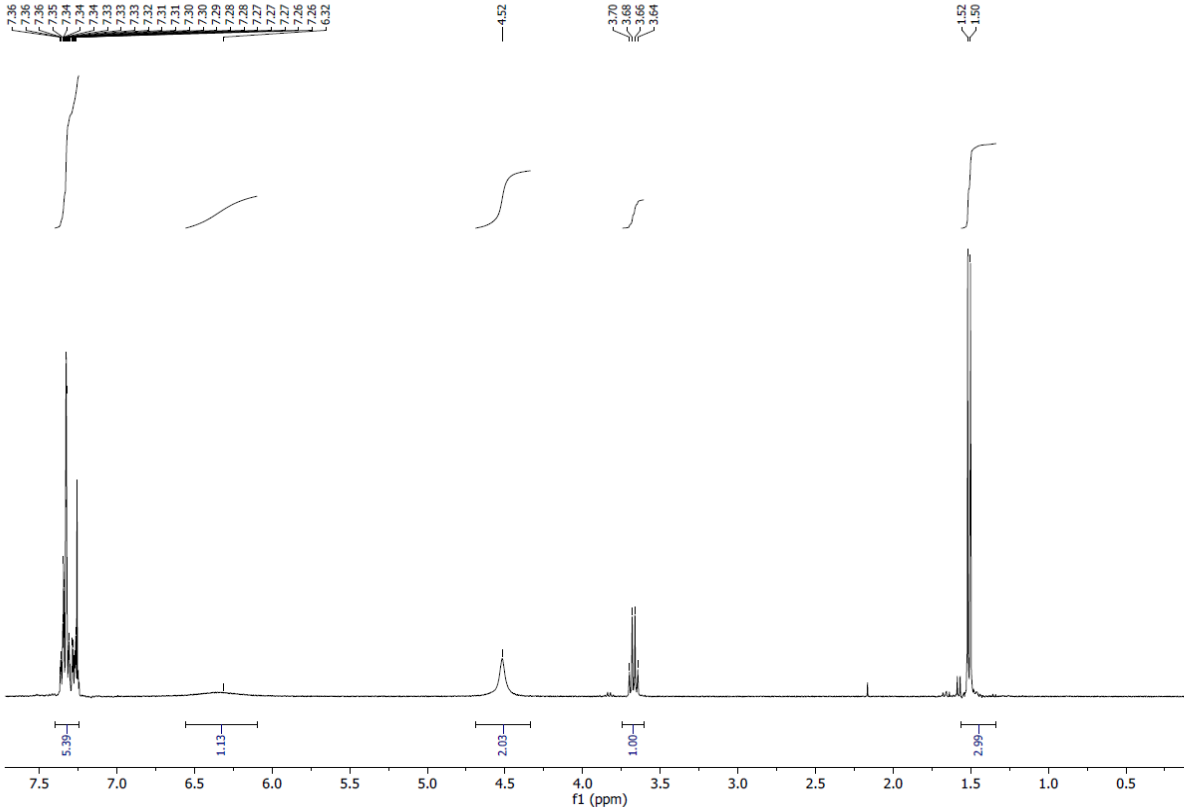

Figure S34. ^{1}H NMR (400 MHz, $CDCl_3$) spectra of *N′*-hydroxy-2-phenylpropanimidamide (**14**)

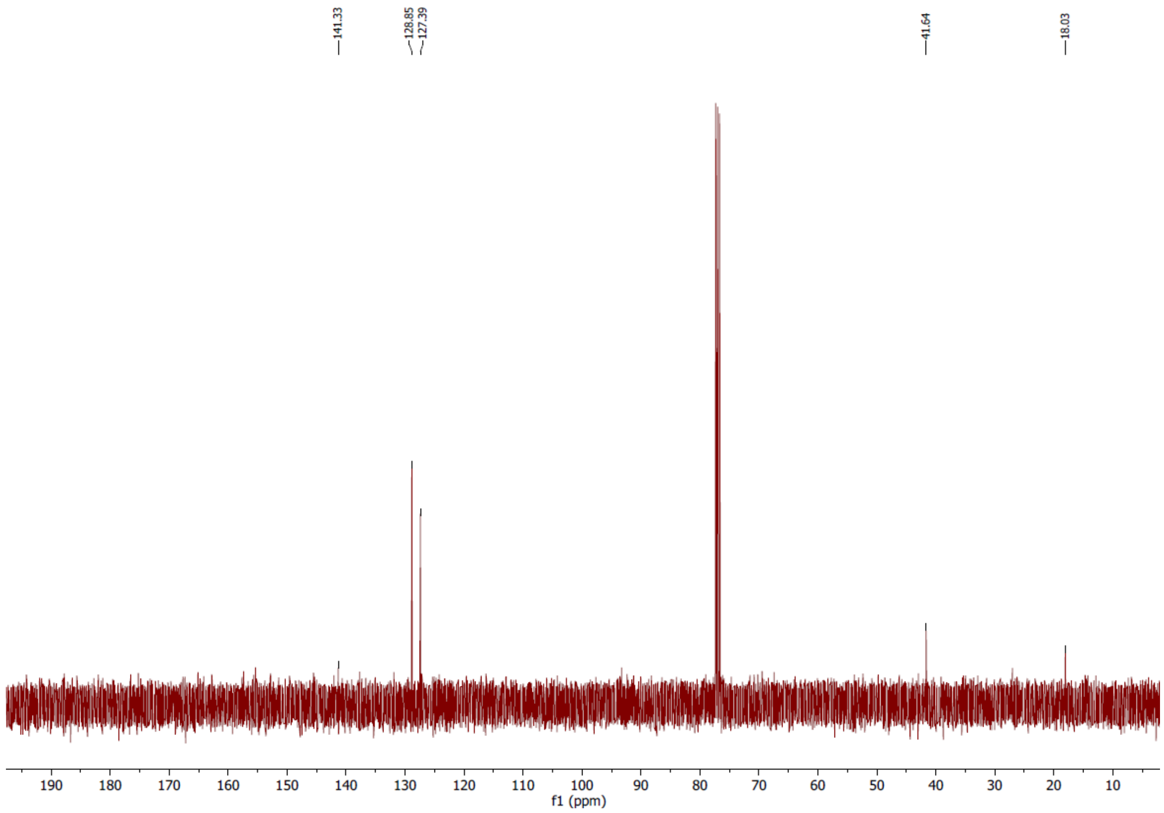

Figure S35. ^{13}C NMR (100 MHz, $CDCl_3$) spectra of *N′*-hydroxy-2-phenylpropanimidamide (**14**)

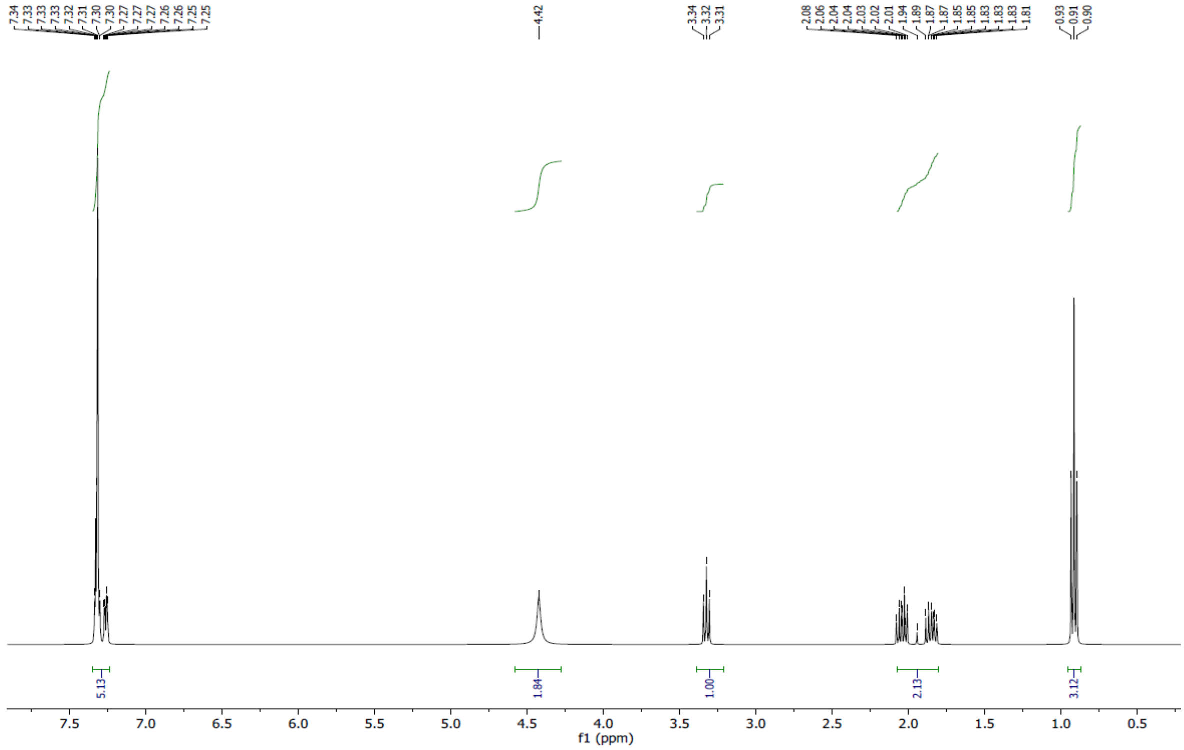

Figure S36. ^{1}H NMR (400 MHz, $CDCl_3$) spectra of *N*-hydroxy-2-phenyl-butyrimidamide (**15**)

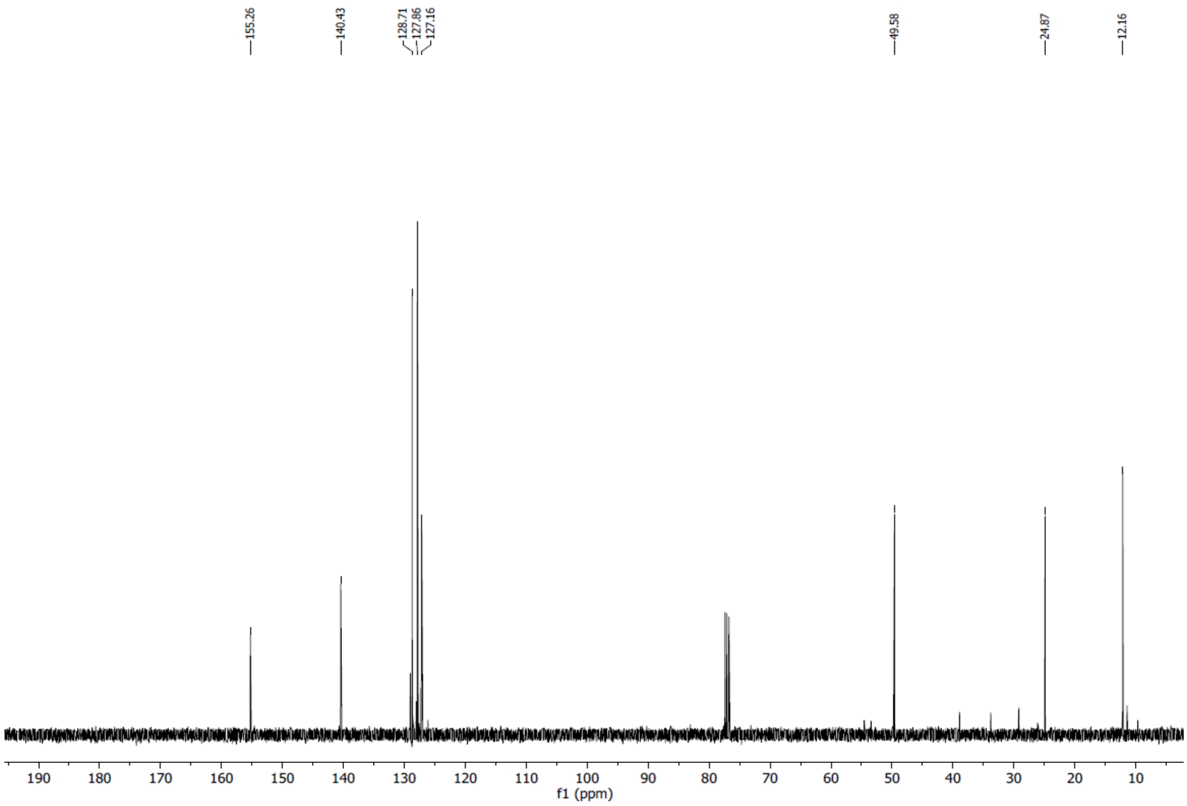

Figure S37. ^{13}C NMR (100 MHz, $CDCl_3$) spectra of *N*-hydroxy-2-phenyl-butyrimidamide (**15**)

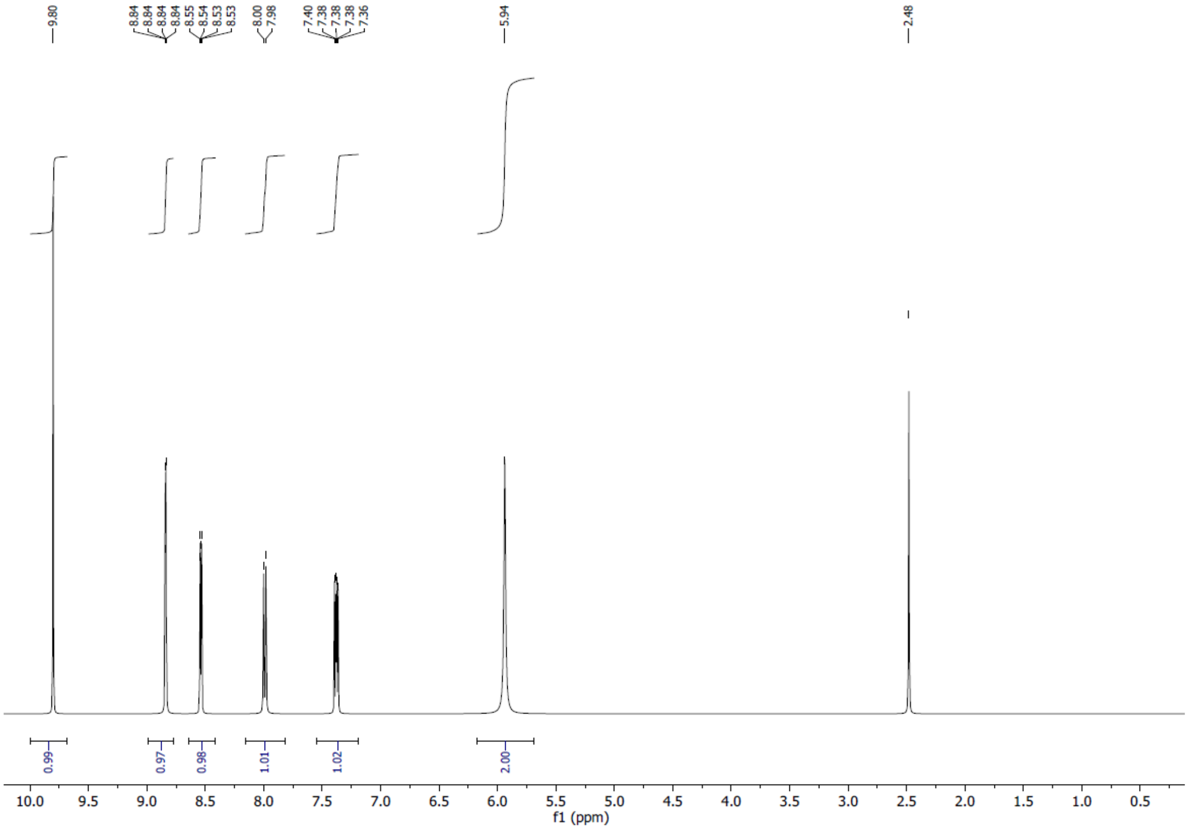

Figure S38. ^{1}H NMR (400 MHz, DMSO-d_6) spectra of nicotinamide oxime (**16**)

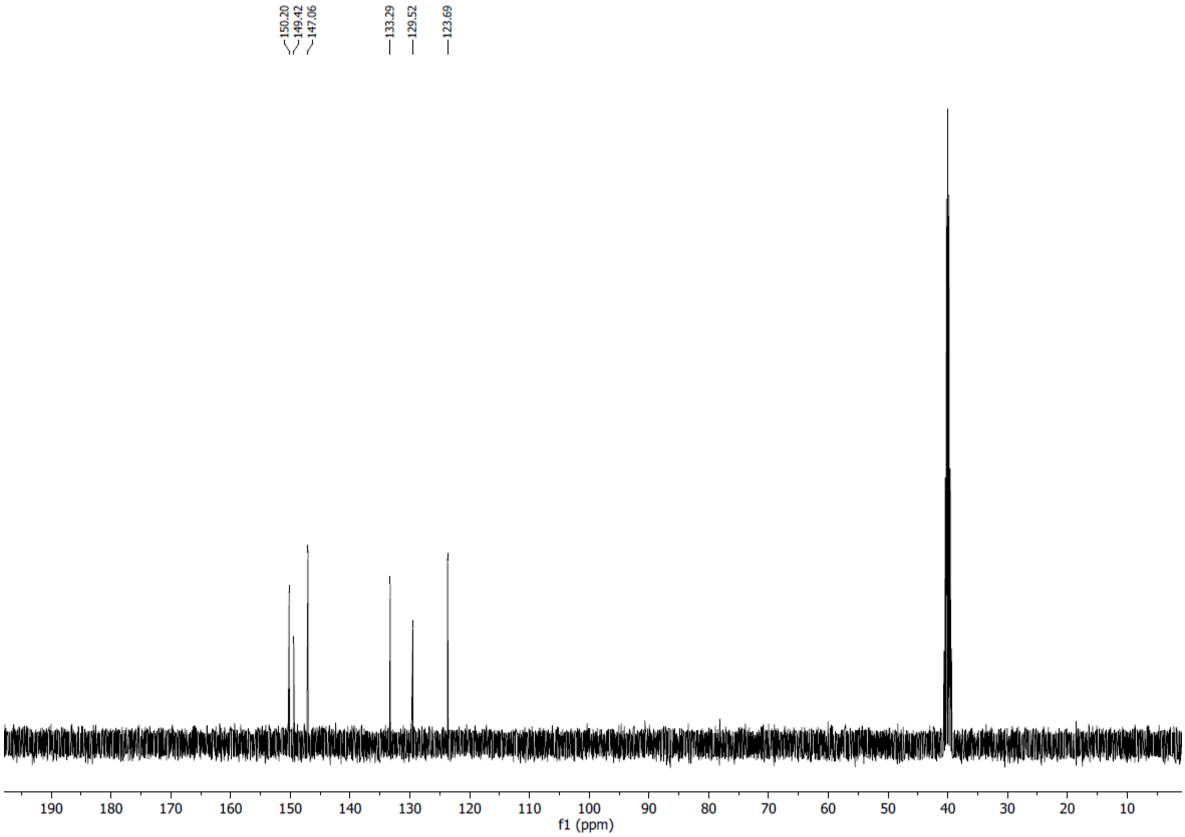

Figure S39. ^{13}C NMR (100 MHz, DMSO-d_6) spectra of nicotinamide oxime (**16**)

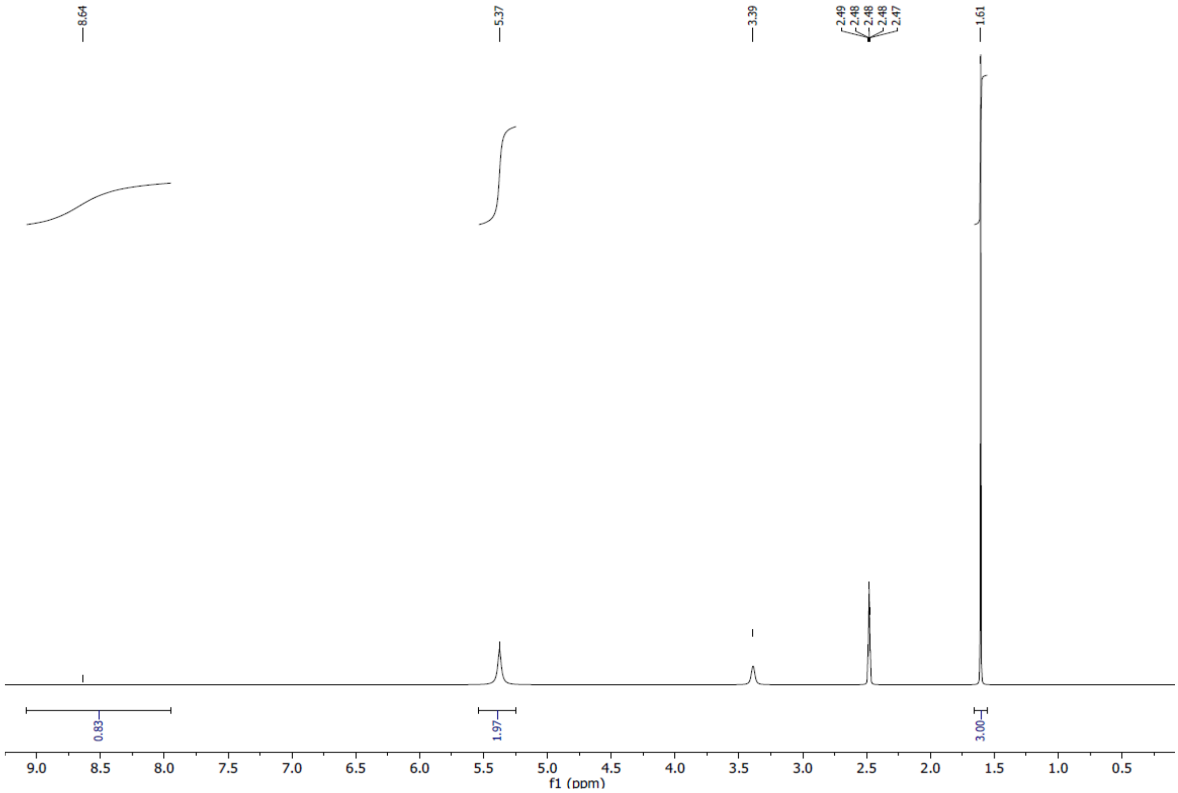

Figure S40. ^{1}H NMR (400 MHz, DMSO-d_6) spectra of acetamide oxime (**17**)

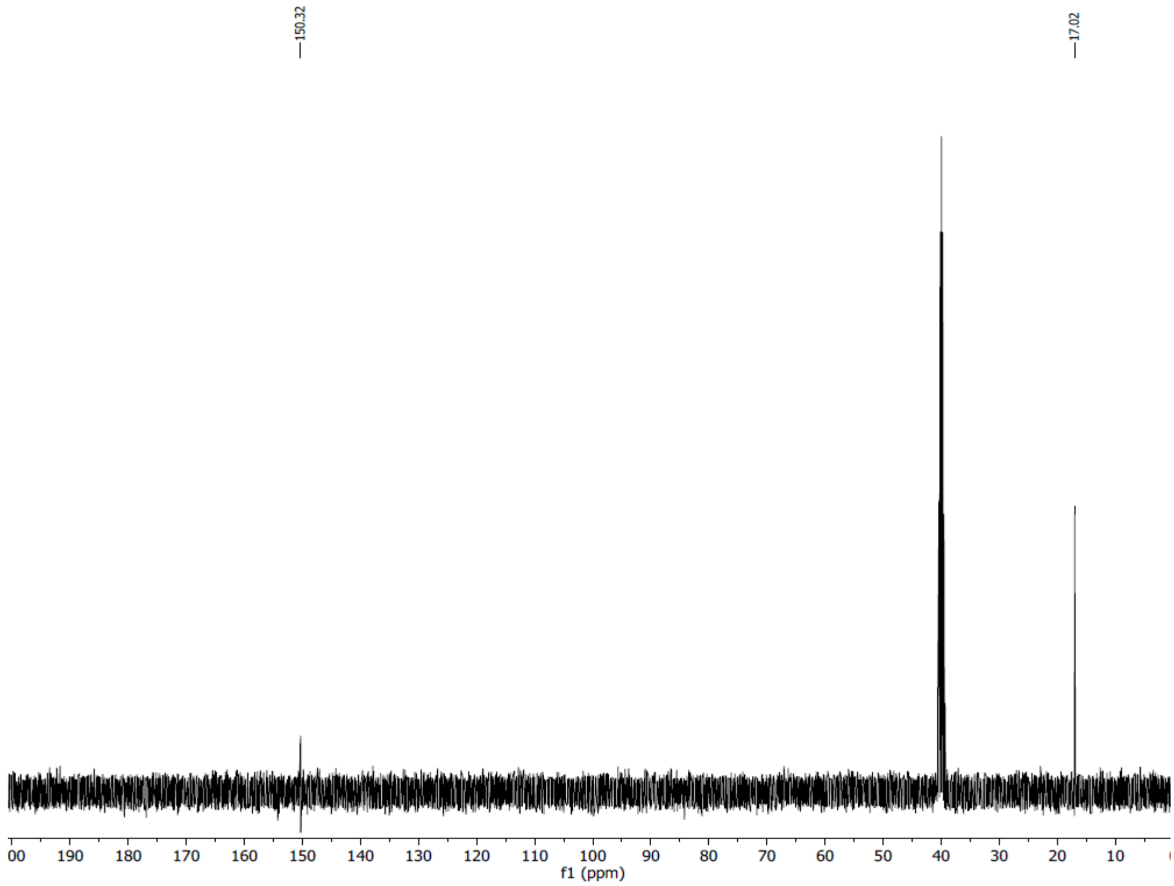

Figure S41. ^{13}C NMR (100 MHz, DMSO-d_6) spectra of acetamide oxime (**17**)

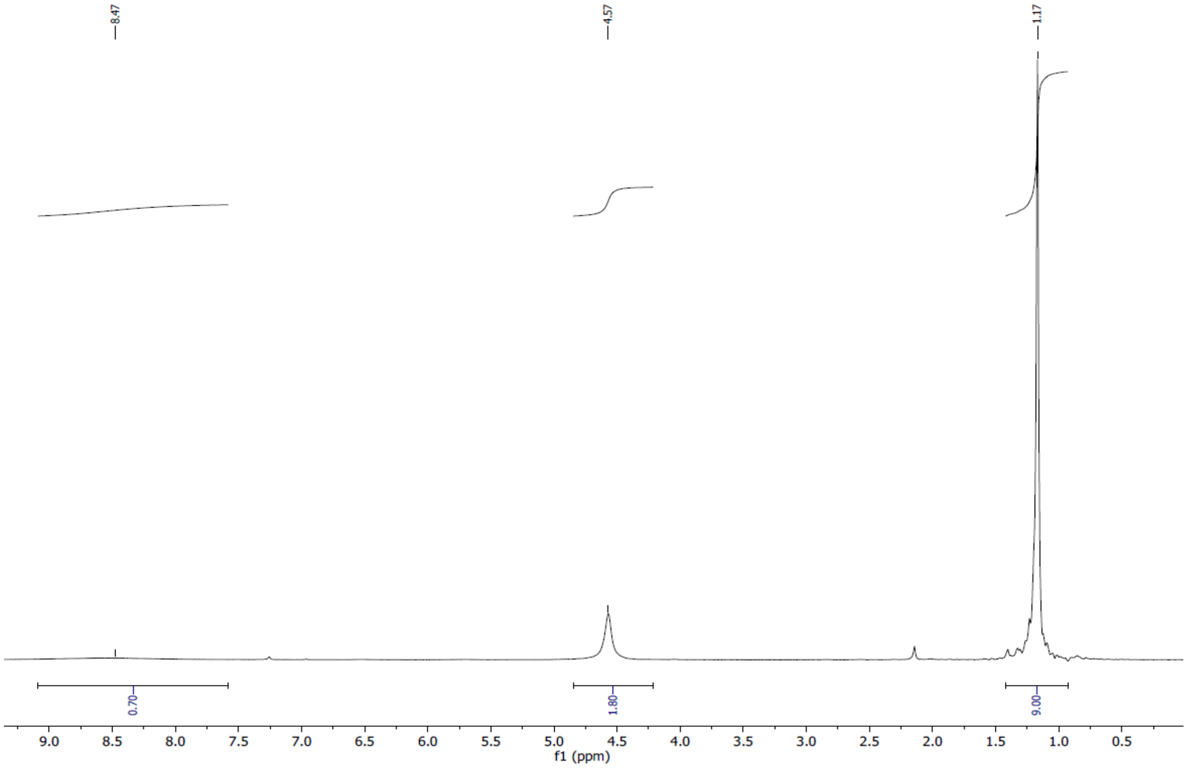

Figure S42. ^{1}H NMR (400 MHz, $CDCl_3$) spectra of *N′*-hydroxypivalimidamide (**18**)

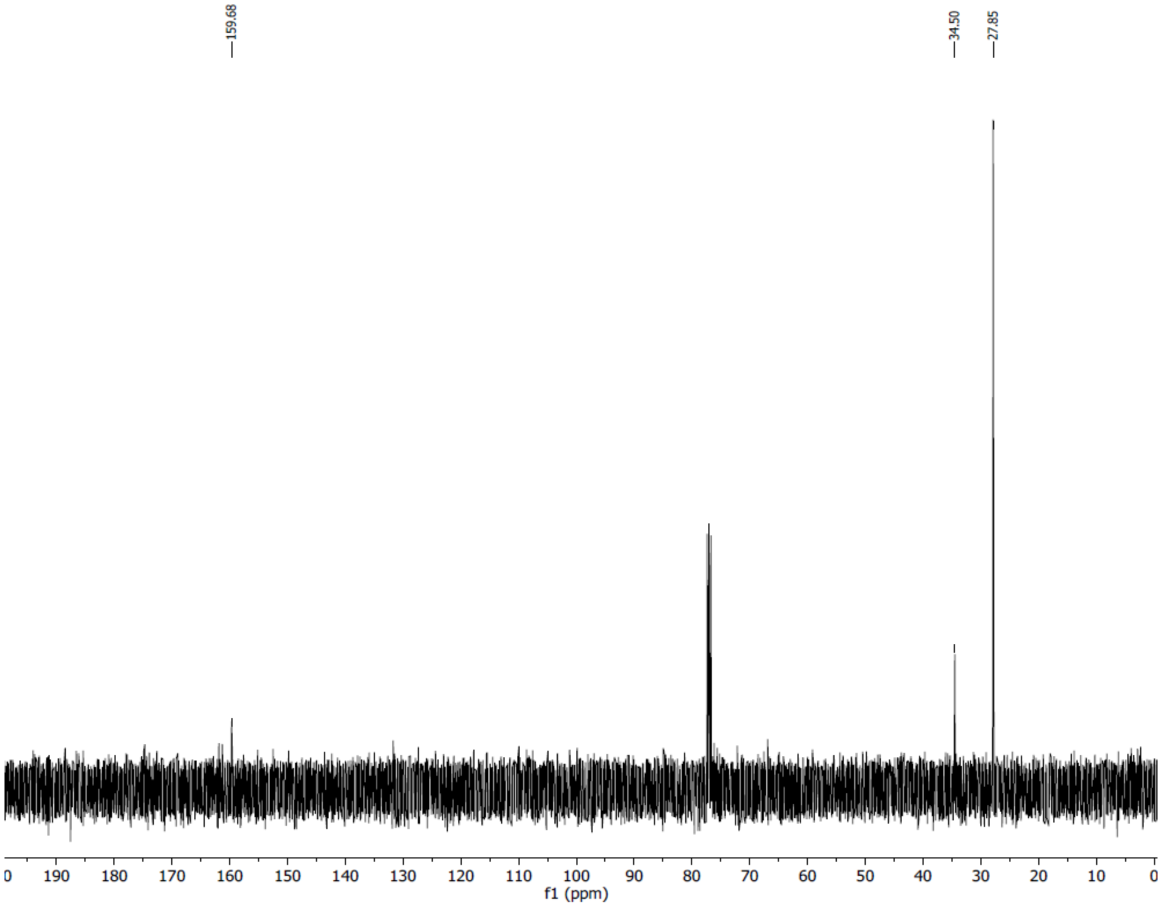

Figure S43. ^{13}C NMR (100 MHz, $CDCl_3$) spectra of *N*′-hydroxypivalimidamide (**18**)

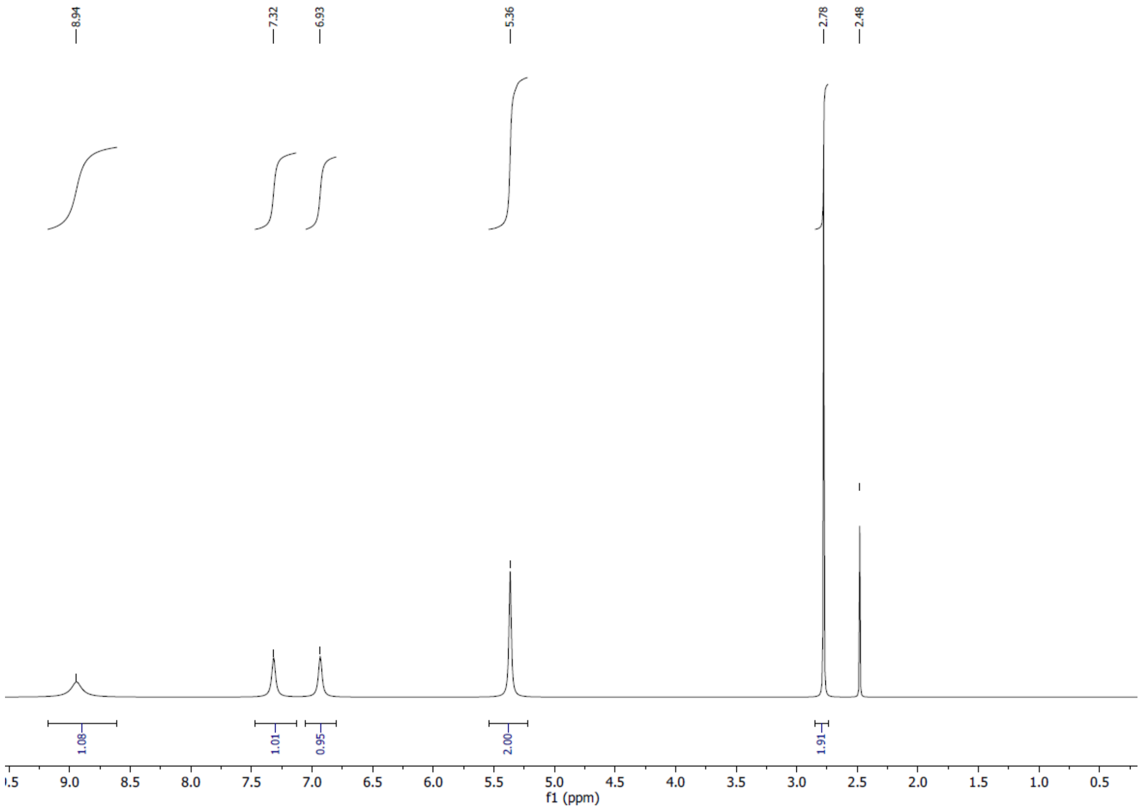

Figure S44. ^1H NMR (400 MHz, DMSO-d_6) spectra of β-amino-β-oximinopropioamide (**19**)

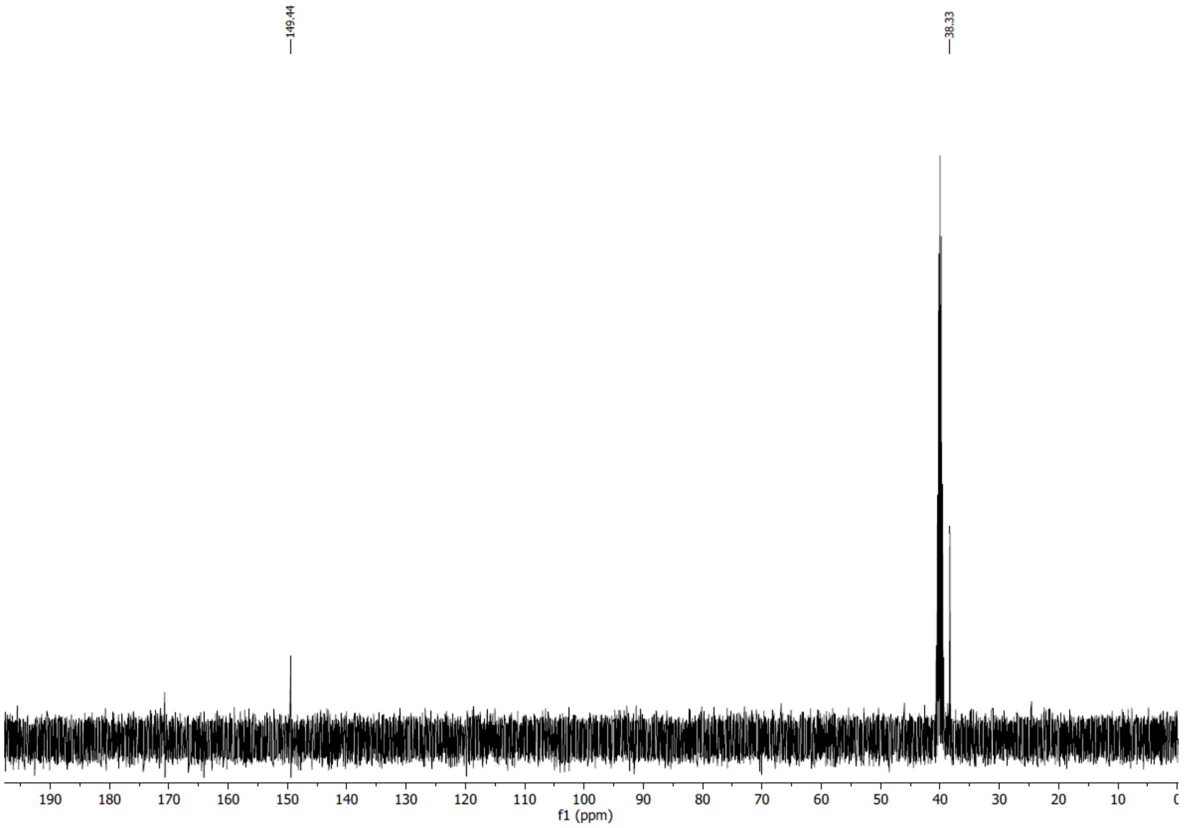

Figure S45. ^{13}C NMR (100 MHz, DMSO-d_6) spectra of β-amino-β-oximinopropioamide (**19**)

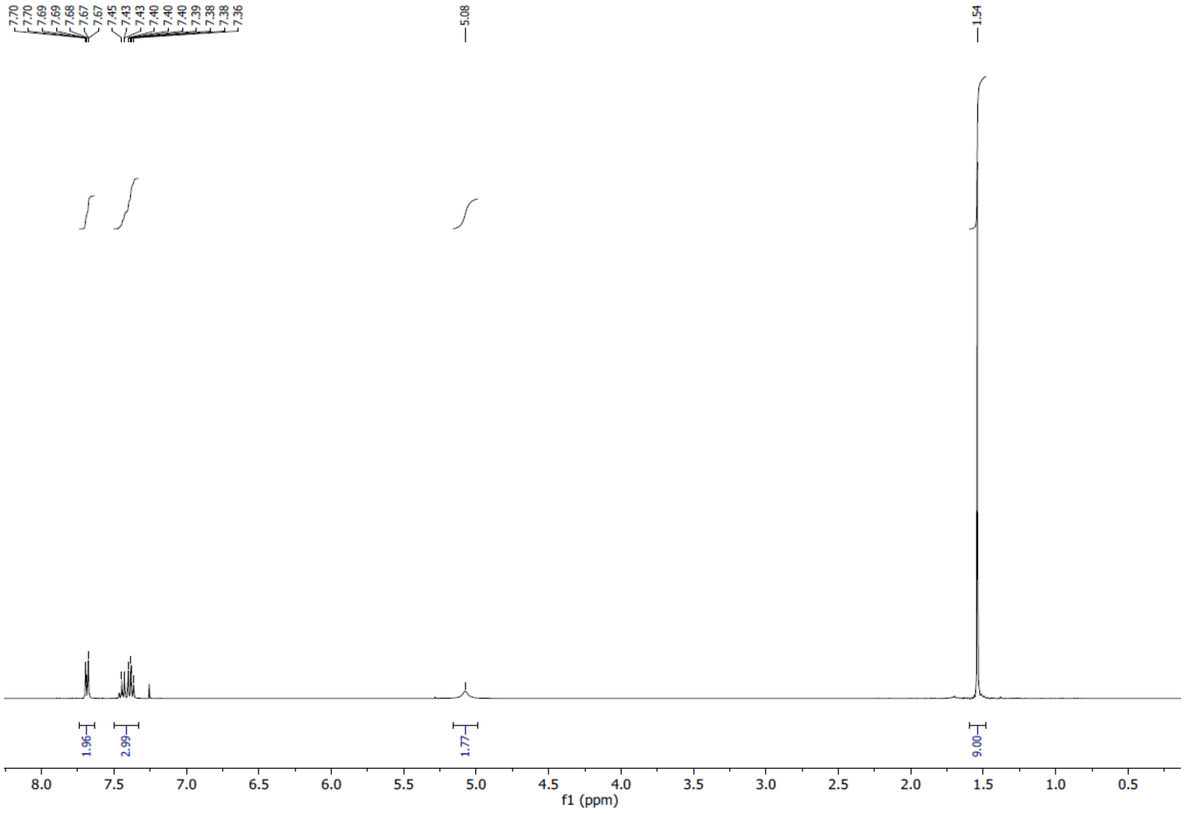

Figure S46. ^1H NMR (400 MHz, $CDCl_3$) spectra of *tert*-butyl [(Z)-(hydroxyimino)(phenyl)methyl]carbamate (**20**)

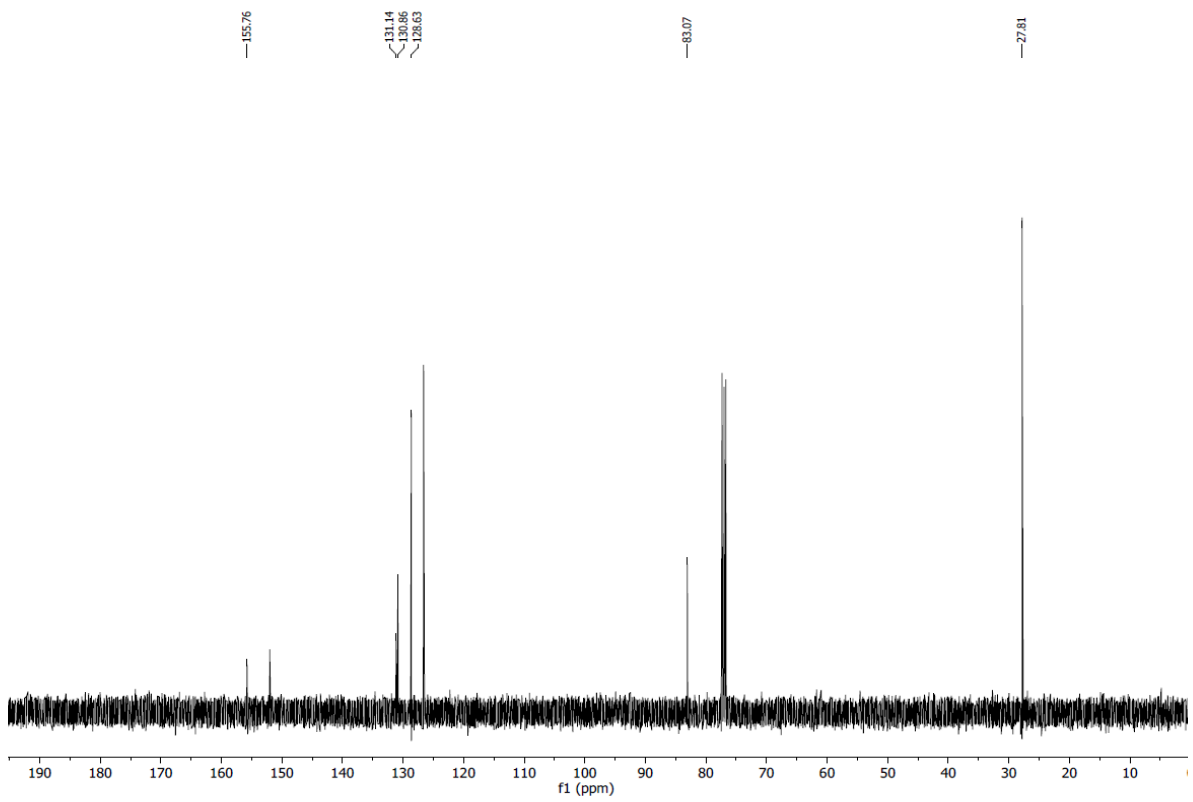

Figure S47. ^{13}C NMR (100 MHz, $CDCl_3$) spectra of *tert*-butyl [(*Z*)-(hydroxyimino)(phenyl)methyl]carbamate (**20**)

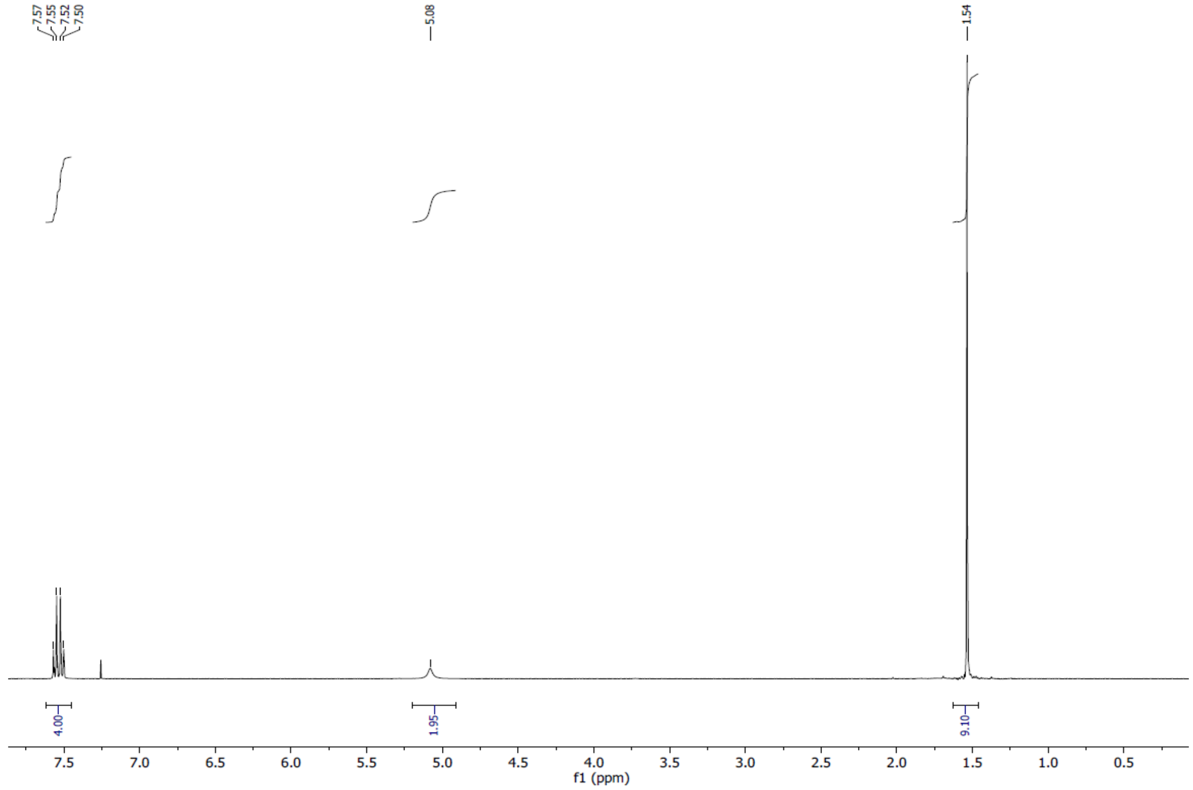

Figure S48. ^{1}H NMR (400 MHz, $CDCl_3$) spectra of *tert*-butyl [(4-bromophenyl)(imino)methyl]carbamate (**21**)

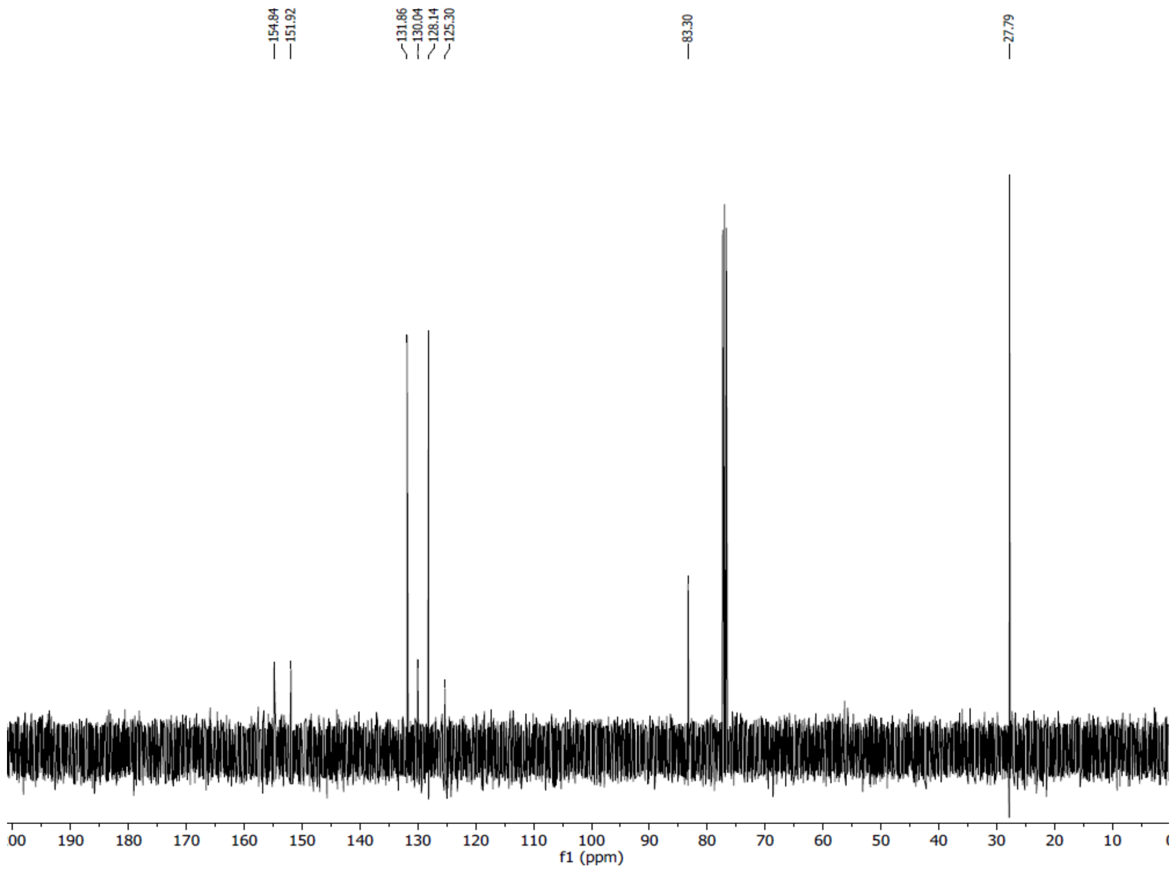

Figure S49. ^{13}C NMR (100 MHz, $CDCl_3$) spectra of *tert*-butyl [(4-bromophenyl)(imino)methyl]carbamate (**21**)

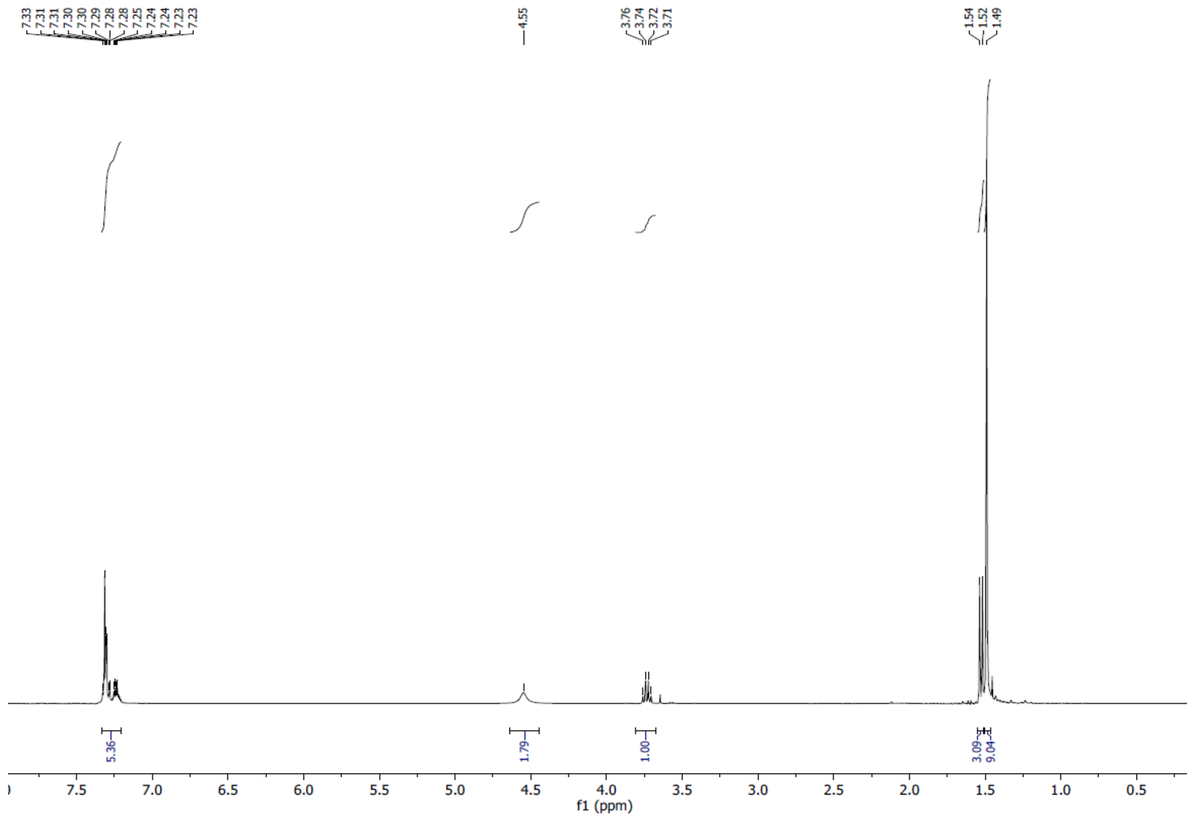

Figure S50. ^{1}H NMR (400 MHz, $CDCl_3$) spectra of *tert*-butyl [*N*-hydroxy-2-phenylpropanimidoyl]carbamate (**22**)

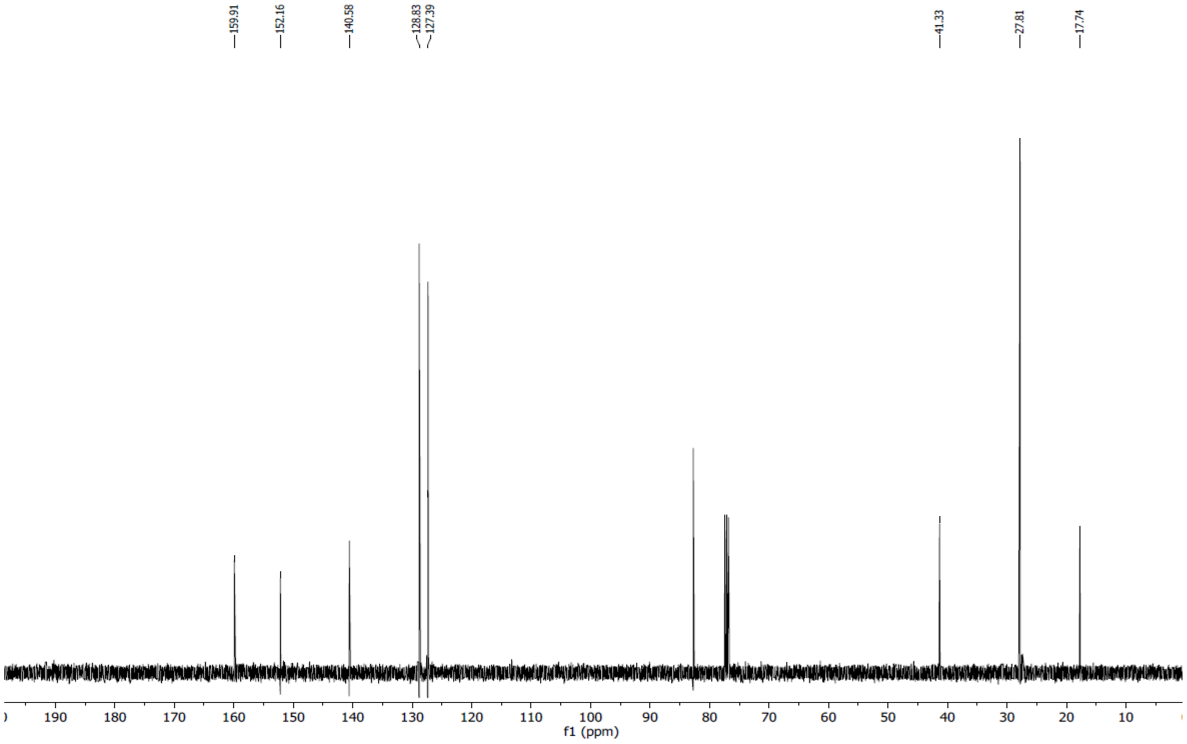

Figure S51. ^{13}C NMR (100 MHz, $CDCl_3$) spectra of *tert*-butyl [*N*-hydroxy-2-phenylpropanimidoyl]carbamate (**22**)

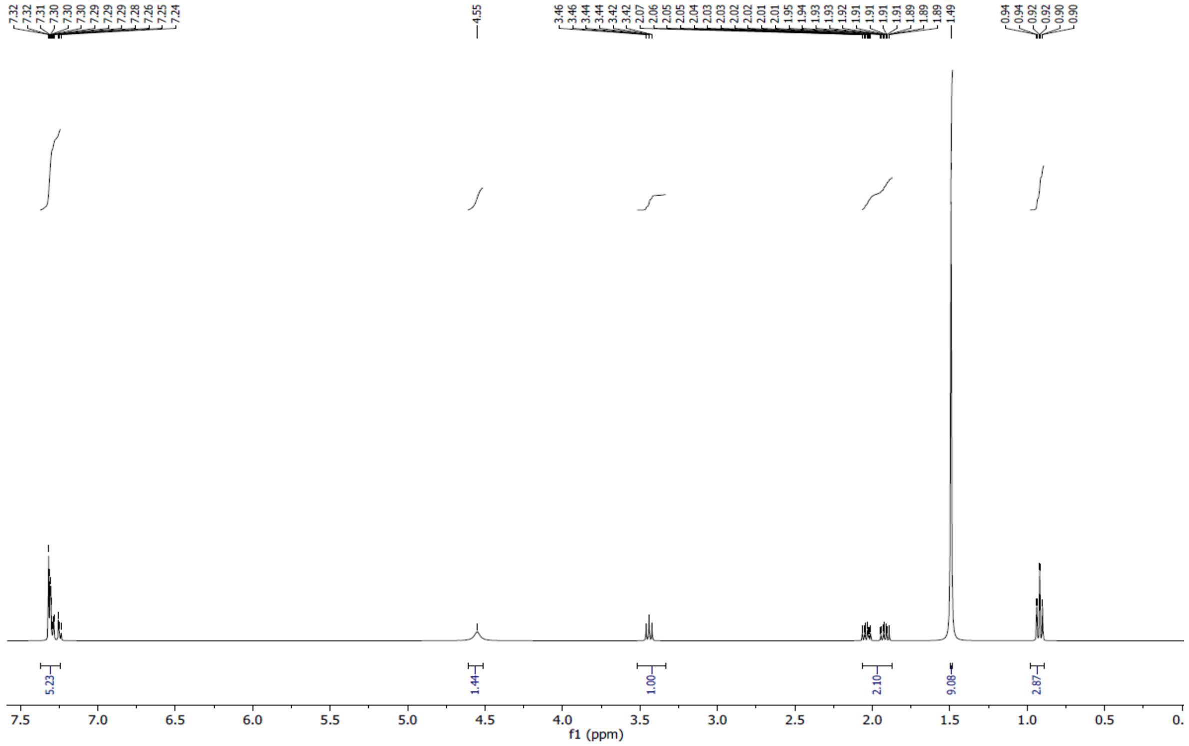

Figure S52. ^{1}H NMR (400 MHz, $CDCl_3$) spectra of *tert*-butyl [*N*-hydroxy-2-phenylbutanimidoyl]carbamate (**23**)

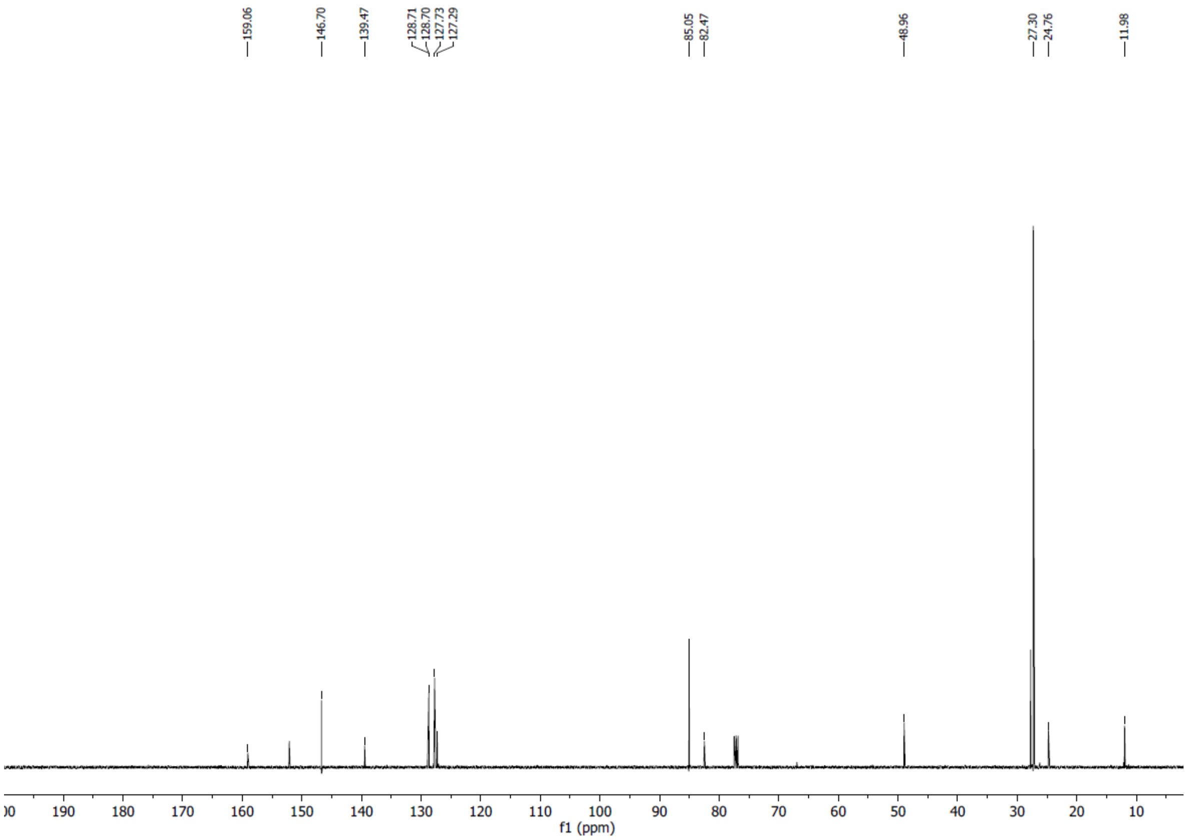

Figure S53. ^{1}H NMR (400 MHz, $CDCl_3$) spectra of *tert*-butyl [*N*-hydroxy-2-phenylbutanimidoyl]carbamate (**23**)

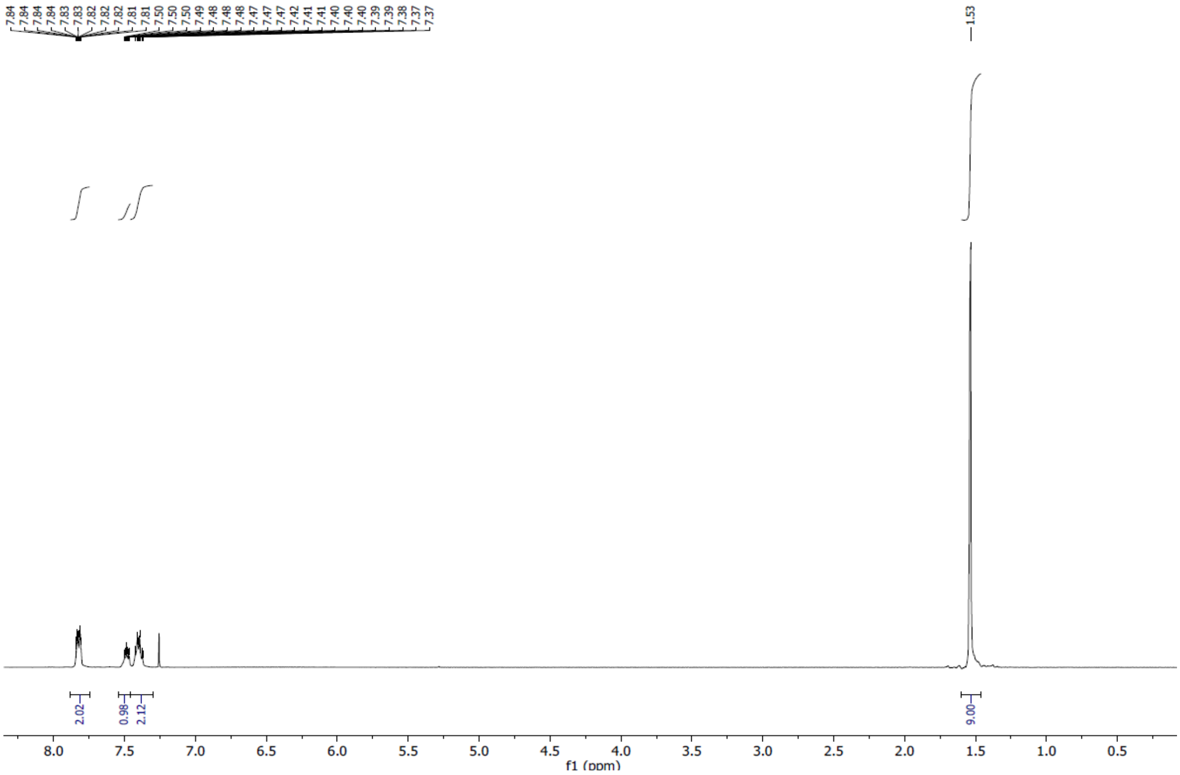

Figure S54. ^{1}H NMR (400 MHz, $CDCl_3$) spectra of *tert*-butyl [imino(phenyl)methyl]carbamate (**24**)

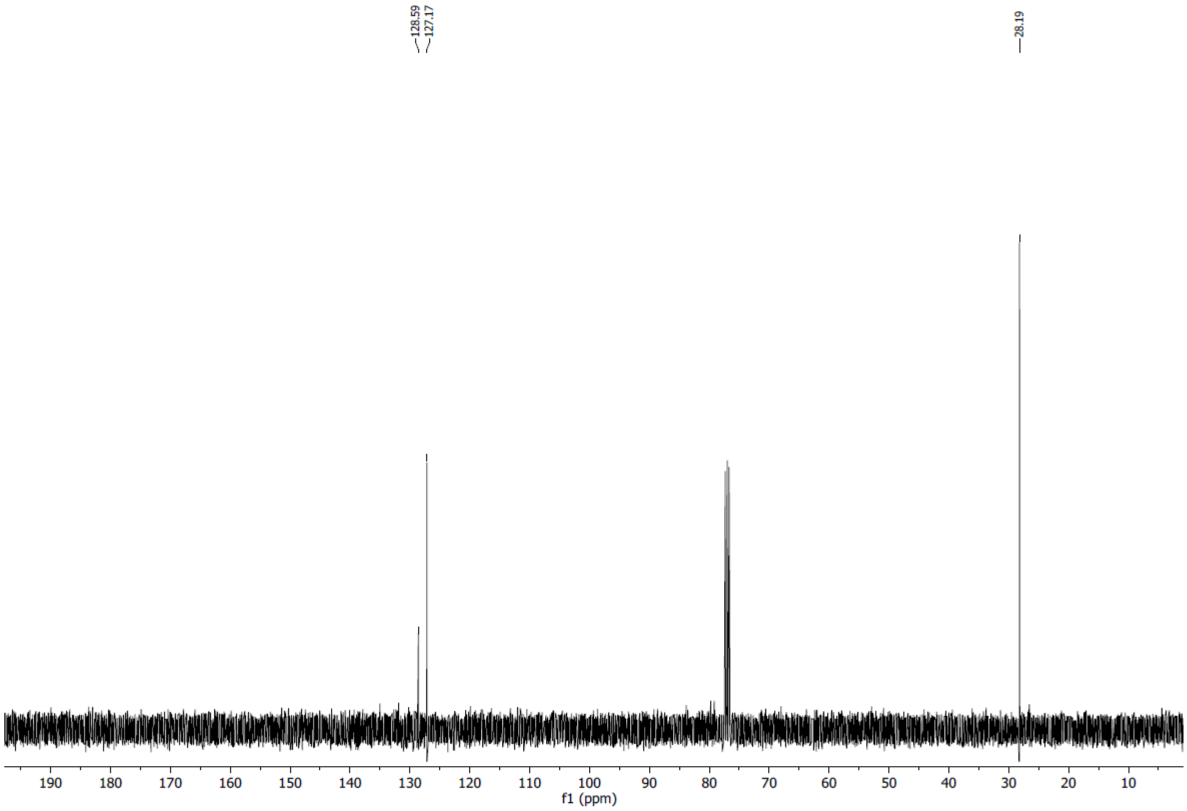

Figure S55. ^{13}C NMR (100 MHz, $CDCl_3$) spectra of *tert*-butyl [imino(phenyl)methyl]carbamate (**24**)

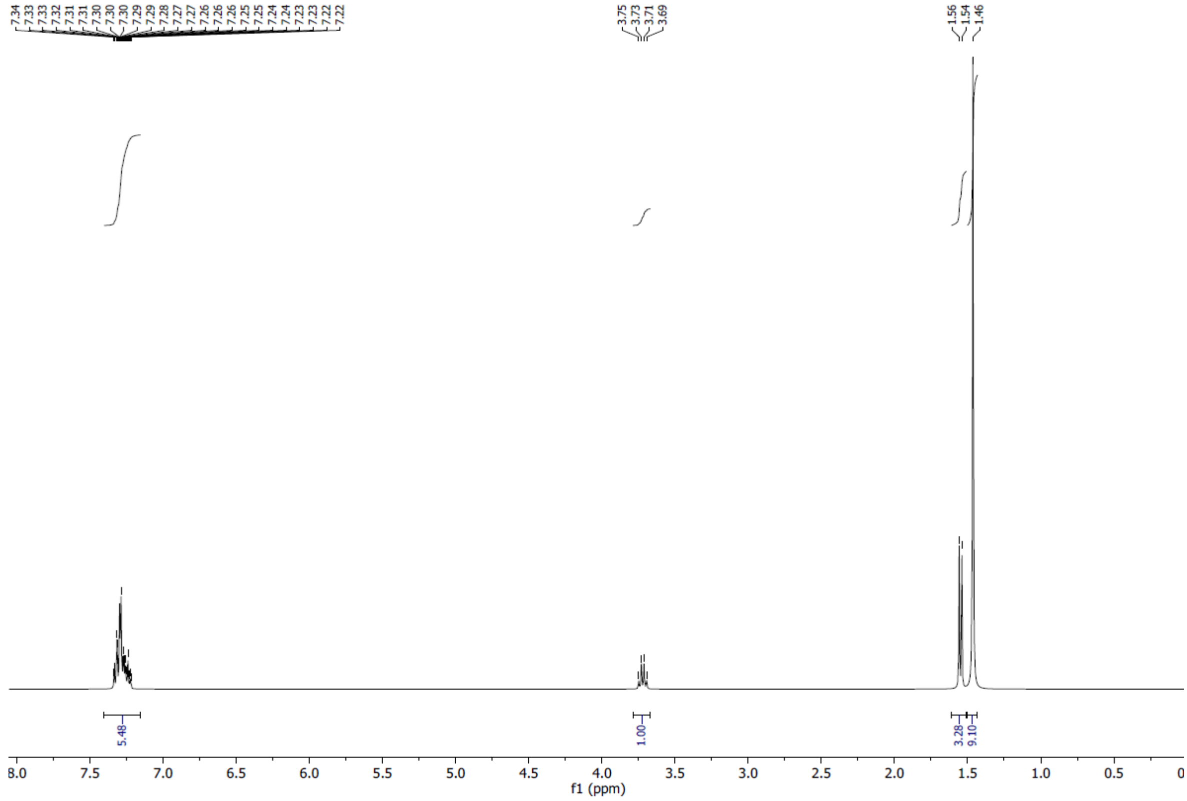

Figure S56. ^{1}H NMR (400 MHz, $CDCl_3$) spectra of *tert*-butyl (2-phenylpropanimidoyl)carbamate (**25**)

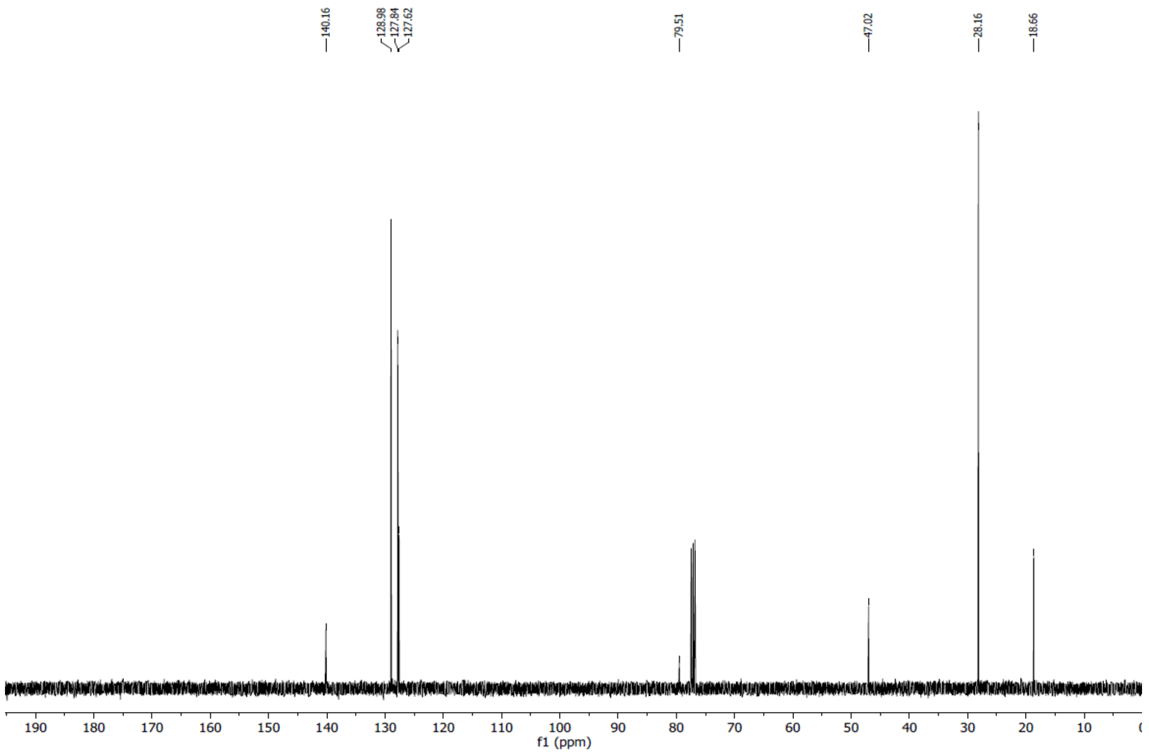

Figure S57. ^{13}C NMR (100 MHz, $CDCl_3$) spectra of *tert*-butyl (2-phenylpropanimidoyl)carbamate (**25**)

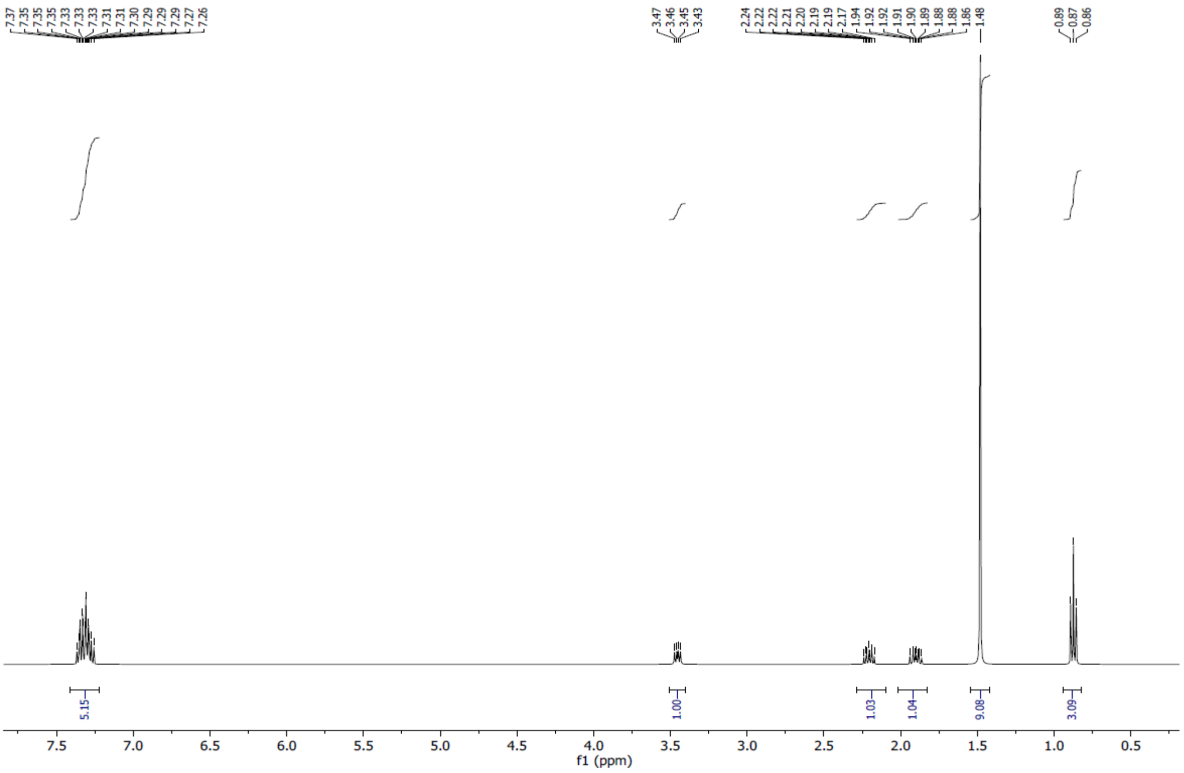

Figure S58. ^{1}H NMR (400 MHz, $CDCl_3$) spectra of *tert*-butyl (2-phenylbutanimidoyl)carbamate (**26**)

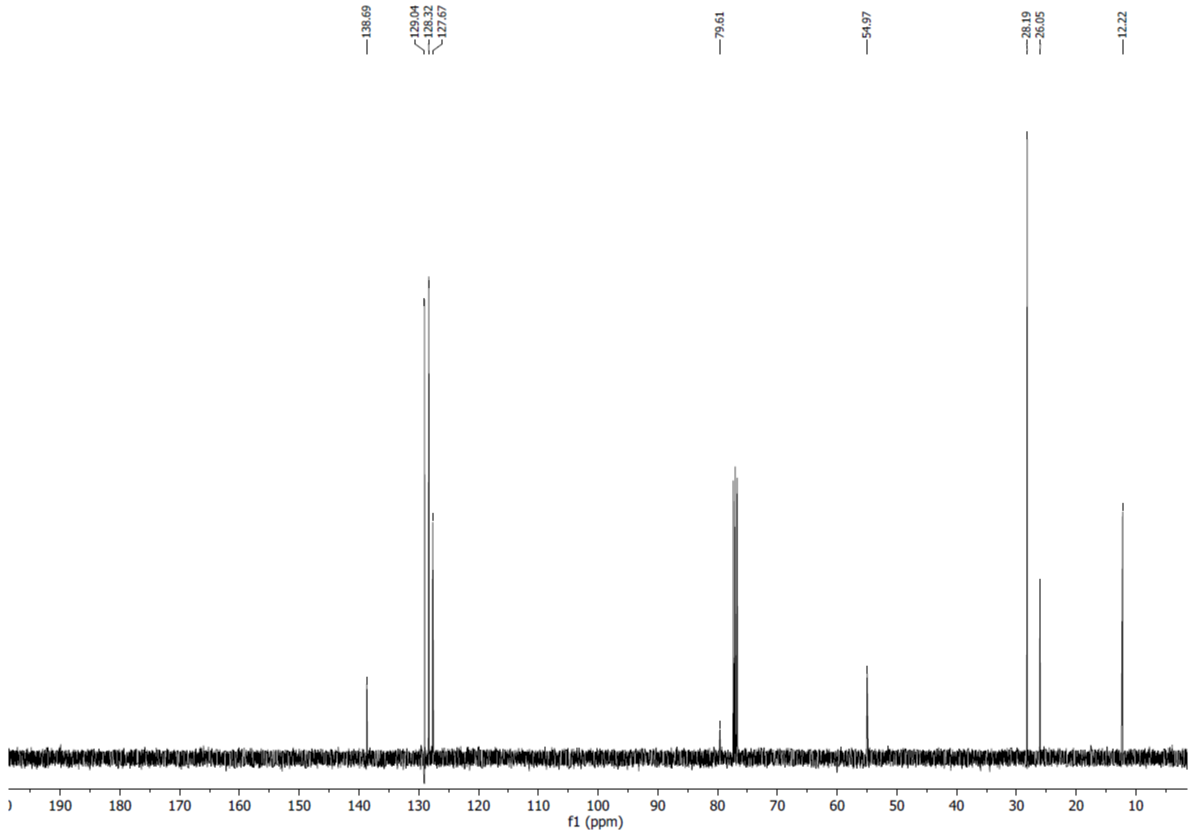

Figure S59. ^{13}C NMR (100 MHz, $CDCl_3$) spectra of *tert*-butyl (2-phenylbutanimidoyl)carbamate (**26**)
